# Hierarchical regulation of oxygen-sensing through coupled transducers in *Arabidopsis thaliana*

**DOI:** 10.1038/s41467-026-77710-x

**Published:** 2026-09-16

**Authors:** Isabel Manrique-Gil, Charlene Dambire, Susana Ubeda-Tomás, Gunjan Sharma, Mohamad Abbas, Cristina Sousa Correia, Andrea Bombana, Ranjan Swarup, Nicholas J. Mitchell, Neil J. Oldham, Michael J. Holdsworth

**Affiliations:** 1 School of Biosciences, University of Nottingham, Loughborough, UK; 2 School of Chemistry, University of Nottingham, University Park, Nottingham, UK

**Keywords:** Flooding, Ubiquitylation, Plant signalling

## Abstract

The sensing of molecular oxygen is an essential component of development and environmental response in both animals and plants. Few substrates of the plant oxygen-sensing PLANT CYSTEINE OXIDASE (PCO) N-degron pathway have been identified. Here we show that HYPOXIA TRANSDUCER1 (HYT1, previously named HYPOXIA-RESPONSIVE UNKNOWN PROTEIN, HUP40), an angiosperm-specific tail anchored-domain protein, is a substrate of this pathway through a Cys-2 dependent N-degron. HYT1 is stabilised in response to acute hypoxia in root tissues and is associated with the endoplasmic reticulum and nucleus. Genetic evidence shows that *HYT1* is an important component in the transduction of oxygen-sensing, required for tolerance to acute hypoxia and other stress responses. Expression of *HYT1* is directly activated by group VII ETHYLENE RESPONSE FACTOR transcription factors (ERFVIIs), that are also pathway substrates. The relationship between ERFVIIs and HYT1 has properties of a coherent feed-forward loop initiated and maintained by hypoxia, providing a mechanism to ensure robust response to reduced oxygen.

## Abstract (web summary)

The PLANT CYSTEINE OXIDASE (PCO) N-degron pathway contributes to oxygen sensing in plants. Here the authors identify HYPOXIA TRANSDUCER1 (HYT1) as a substrate of PCO and show it forms a coherent feed-forward loop with ERFVII transcription factors to ensure a robust response to reduced oxygen.

## Introduction

**Fig. 1:**
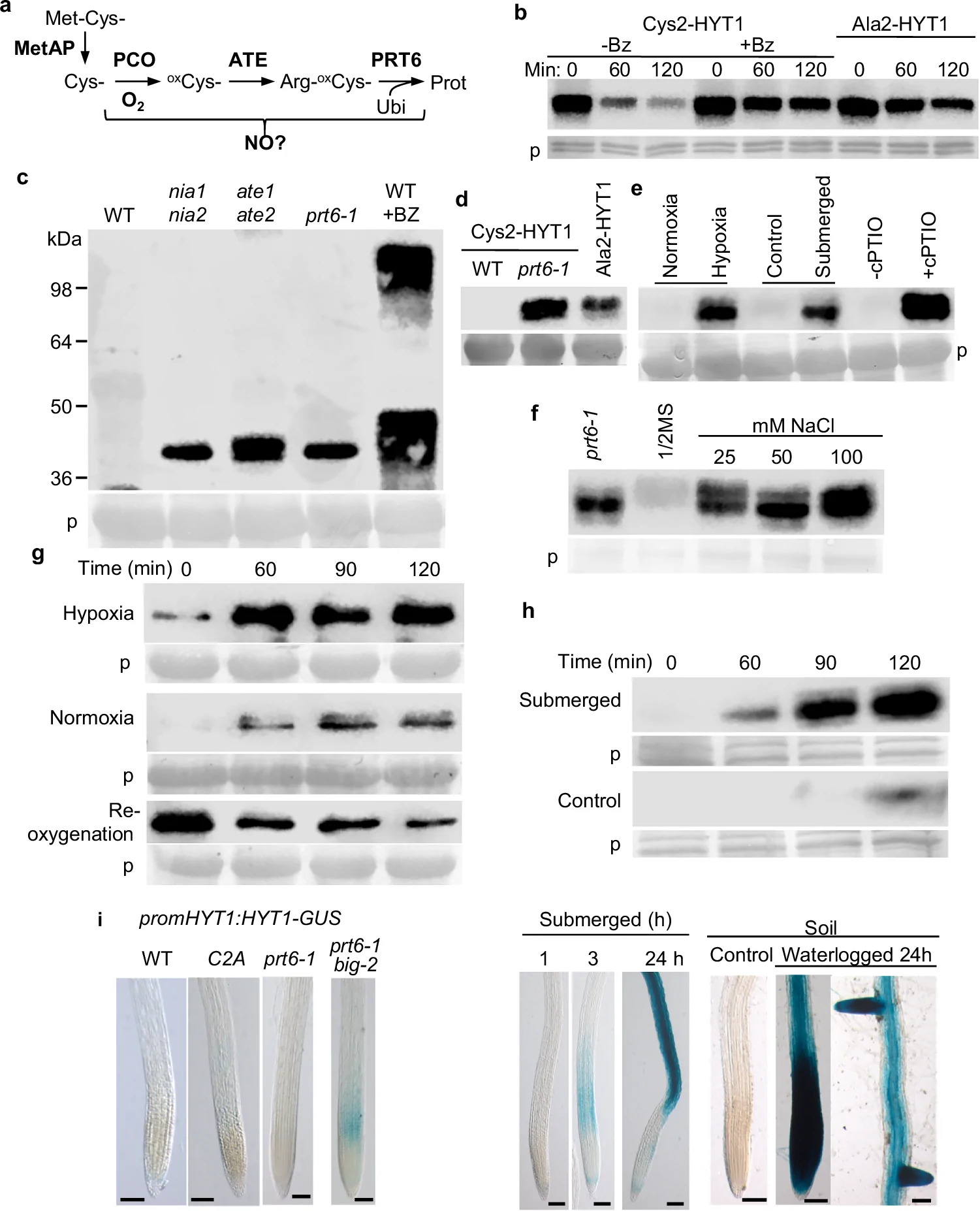
HYT1 is a substrate of the ATE/PRT6 N-degron pathway through Cys2. **a** Schematic of the PCO branch of the PRT6 N-degron pathway. MetAP, methionine aminopeptidase; ATE, arginyl transferase; PCO, PLANT CYSTEINE OXIDASE; ox, oxidised cysteine; Ubi, ubiquitin; Prot, proteasome. Three letter amino-acid abbreviations are used. The position of NO action in the pathway is not known (?). **b** Western blot showing HYT1-3xHA accumulation in rabbit reticulocyte lysate with or without proteasome inhibitor bortezomib (BZ). Time after cycloheximide addition (mins). **c** HYT1-3xHA accumulation in WT +/- bortezomib, *prt6-1*, *ate1 ate2* and *nia1 nia2* mutant seedlings from the transgene *35S:UFT-Cys2-HYT1-3xHA*. **d** Cys2-HYT1-3xHA accumulation in WT or *prt6-1*, and Cys2Ala-HYT1-3xHA accumulation in WT, seedlings. **e** Western blot analysis of environmental control of HYT1-3xHA accumulation in seedlings. Hypoxia <0.5 % O_2_, 2 h; Submerged 1 h, cPTIO 100 µM 2 h. **f** HYT1-3xHA accumulation in WT seedlings grown for 7 days on media supplemented with NaCl. **g** Time-course analysis of HYT1-3xHA (from the transgene *35S:UFT-Cys2-HYT1-3xHA*) accumulation in WT seedlings in response to ambient dark hypoxia ( < 0.5 % O_2_) and re-oxygenation. Times are from beginning of hypoxia treatment or release from the hypoxia chamber. **h** Time-course analysis of HYT1-3xHA (from the transgene *35S:UFT-Cys2-HYT1-3xHA*) accumulation in WT seedlings in response to dark submergence. Seedlings were submerged in the dark, or left only in the dark (control), for the indicated times. **i** Histochemical staining to reveal GUS accumulation from the *promHYT1:HYT1-GUS* translational reporter in WT and mutant 7-day old seedlings and in WT in response to submergence, or waterlogged mature plants grown in mixed sandy-clay loam soil. Bar, 0.1 mm. Ponceau staining of Western blots is shown.

Oxygen is a required molecule for mitochondrial aerobic respiration and many biochemical pathways. Reduced oxygen is encountered by organisms either externally by environmental change (acute hypoxia) or internally due to developmental or physiological change (chronic hypoxia)^1^. Therefore, sensing of cellular oxygen levels is an important component of growth and development in aerobic organisms, including land plants and metazoan animals^2,3^. Major known mechanisms in both groups link the oxygen-dependent proteasomal degradation of specific transcription factors to downstream biochemical and physiological responses. In metazoans, the Hypoxia Inducible Factor alpha (HIFα) transcription factor, as part of a heterodimeric HIF-complex, activates gene expression in hypoxia (for example, of the Erythropoietin (*EPO*) gene) but is destroyed through the Ubiquitin Proteasome system (UPS) following HIF prolyl hydroxylase domain protein (PHD)-mediated hydroxylation of internal proline residues in the presence of oxygen^4^. The only known oxygen-sensing mechanism in plants (studied predominantly in the angiosperm *Arabidopsis thaliana*) requires the action of PCO oxygen sensors to oxidise amino-terminal Cys of substrates of the PROTEOLYSIS6 (PRT6) N-degron pathway (Fig. 1a)^3,5^. Known substrate transducer proteins all initiate Met1-Cys2, and co-translational removal of Met1 exposes Cys2 as the amino-terminal (Nt) residue. In oxygen-replete conditions (normoxia) PCO-dependent oxidation of Nt-Cys leads to amino-terminal addition of Arg via ARGINYL TRANSFERASE (ATE) activity and subsequent recognition by the E3 ligase PRT6 (and ancillary activity of BIG^6^), ubiquitylation and destruction via the UPS^7,8^. Recently, it was postulated that other monooxygenase enzyme activity in addition to PCOs may be required to oxidise Nt-Cys to sulphonic acid, as this form is found in both plant extracts and animal cell cultures^8,9^. This pathway, which also requires nitric oxide (NO) for substrate destabilisation^10,11^, is highly conserved in eukaryotes^3^, and the mammalian counterpart of PCO, cysteamine (2-aminoethanethiol) dioxygenase (ADO), was recently identified^12^. In mammalian proteomes, only three groups of ADO substrates (initiating Met1-Cys2) have been identified: REGULATOR OF G PROTEIN SIGNALLING (RGS) 4,5,16, acyl-CoA dehydrogenase family, member 10 (ACAD10) and the cytokine Interleukin (IL)−32; none are transcription associated^10,12–14^. In contrast, all three identified substrates of the plant PCO N-degron pathway are associated with transcription, including five group VII ETHYLENE RESPONSE FACTOR (ERFVII)^15,16^ and one LITTLE ZIPPER2 (ZPR2)^17^ transcription factors, and polycomb repressive complex (PRC)2 component VERNALISATION2 (VRN2)^18^. Both ERFVIIs and VRN2 enhance tolerance to acute hypoxia, and ERFVIIs have been shown to activate transcription of many hypoxia-induced genes, including most of a core set of 49 conserved hypoxia-responsive genes^8,19^. ERFVII stability can be regulated via hormonal signalling; ethylene accumulation leads to the induction of PHYTOGLOBIN (PGB)1 that reduces endogenous NO levels, thereby stabilising ERFVIIs^20^. All three plant substrates are stabilised in response to chronic developmental hypoxia in shoot apical meristems^17,20,21^. Hypoxia can also be an important component of the pathology of infection, for example, ERFVII stabilisation is employed by *Plasmodiophora brassicae* as part of clubroot disease^22^, and during both *Botrytis cinerea*^23^ and *Sclerotinia sclerotiorum* infection^24^.

Eukaryotic organisms contain a diverse, restricted group of proteins that initiate Met1-Cys2, including approximately 250 proteins in *A. thaliana*^25^. In this paper, we identified the Cys2 protein HYPOXIA TRANSDUCER (HYT)1, which is also a conserved hypoxia-induced gene, as a substrate of the N-degron pathway. HYT1 half-life is dependent on ambient oxygen levels, protein expression is root-associated, and transcription is regulated by the ERFVIIs. HYT1 is an important component of tolerance to acute hypoxia.

## Results

### Identification of HYT1 as a Cys2- substrate of the PCO N-degron pathway

To identify potential substrates of the plant oxygen-sensing pathway, we analysed the intersection between *A. thaliana* 49 genes ubiquitously up-regulated at the transcript level in hypoxia^19^ and *A. thaliana* proteins that initiate Met1-Cys2 (Supplementary Data 1). We identified three genes, the ERFVIIs HYPOXIA RESPONSIVE ERF (HRE)1 and 2, and AT4G24110, previously named HYPOXIA-RESPONSIVE UNKNOWN PROTEIN (HUP)40^26^. Because AT4G24110 is identified here as a substrate of the PCO N-degron oxygen-sensing pathway, we renamed it HYPOXIA TRANSDUCER (HYT)1. We analysed the potential for HYT1 to be conditionally stable, dependent on Cys2. Cys2-HYT1 was expressed as part of a Ubiquitin Fusion Technique (UFT^27^) construct using the rabbit reticulocyte lysate as a heterologous system that contains all components of the mammalian Arg/N-degron pathway^15^. In the presence of cycloheximide, Cys2-HYT1-3xHA (from pUFT-Cys2-HYT1-3xHA) was degraded, but less when the proteasome was inhibited with bortezomib, or for a Cys2Ala substitution (Fig. 1b). Encouraged by this result, we analysed the stability of Cys2-HYT1-3xHA in vivo in *A. thaliana* seedlings containing the transgene *35S:UFT-Cys2-HYT1-3xHA*. In transgenic Col-0 accession (wild-type; WT) seedlings HYT1-3xHA accumulated to only very low levels (Fig. 1c), but showed strong accumulation following treatment with bortezomib, and in *prt6-1*, *ate1 ate2* and *nia1 nia2* (that has very low endogenous NO levels^28^) backgrounds. A Cys2Ala substitution in WT (transgene *35S:UFT-Cys2Ala-HYT1-3xHA*) also resulted in constitutive stabilisation (Fig. 1d). These results demonstrate that HYT1 is a bona fide physiological substrate of the PRT6 N-degron pathway through Nt-Cys. We investigated the influence of environmental perturbations and found that reduced oxygen conditions of submergence or hypoxia, or reduced NO levels in the presence of cPTIO, or high NaCl all led to accumulation of HYT1-3xHA (Fig. 1e, f). The time course of accumulation of HYT1-3xHA driven from the 35S promoter in response to submergence and hypoxia was similar to that previously observed for RAP2.3^8^ (Fig. 1g, h) when driven from the 35S promoter.

The developmental stability of HYT1 in seedlings was analysed to define the role of the PRT6 N-degron pathway and environmental stress in controlling the accumulation of HYT1 transcript and protein. Transgenic plants were generated including 2kbp of *HYT1* upstream promoter region and the entire gene region tagged at the C-terminus with β-glucuronidase (GUS), in both the Cys-2 and Cys-2-Ala versions (*promHYT1:HYT1-GUS* or *promHYT1:Ala-2-HYT1-GUS*) in WT, *prt6-1* or *prt6-1 big-2* backgrounds. Under ambient oxygen growth conditions on media, HYT1-GUS accumulation was not observed in WT seedlings (Fig. 1i, Supplementary Fig. 1a). The Ala-2-HYT1-GUS was detected in lateral roots and the shoot apical meristem (Supplementary Fig. 1b). In addition to sites of accumulation observed for Ala-2-HYT1-GUS, in *prt6-1* HYT1-GUS also accumulated in the root vasculature, and the level of accumulation was much greater in *prt6-1 big-2* (Supplementary Fig. 1c, d). HYT1-GUS accumulated in the root elongation and maturation zones from 3 h submergence. Accumulation of HYT1-GUS also occurred in roots of mature plants grown in mixed sandy-clay loam soil after 24 h waterlogging, but not in well-watered control plants grown in soil (Fig. 1i).

### The amino-terminal Cys of HYT1 is oxidised by PCOs in the presence of molecular oxygen

**Fig. 2:**
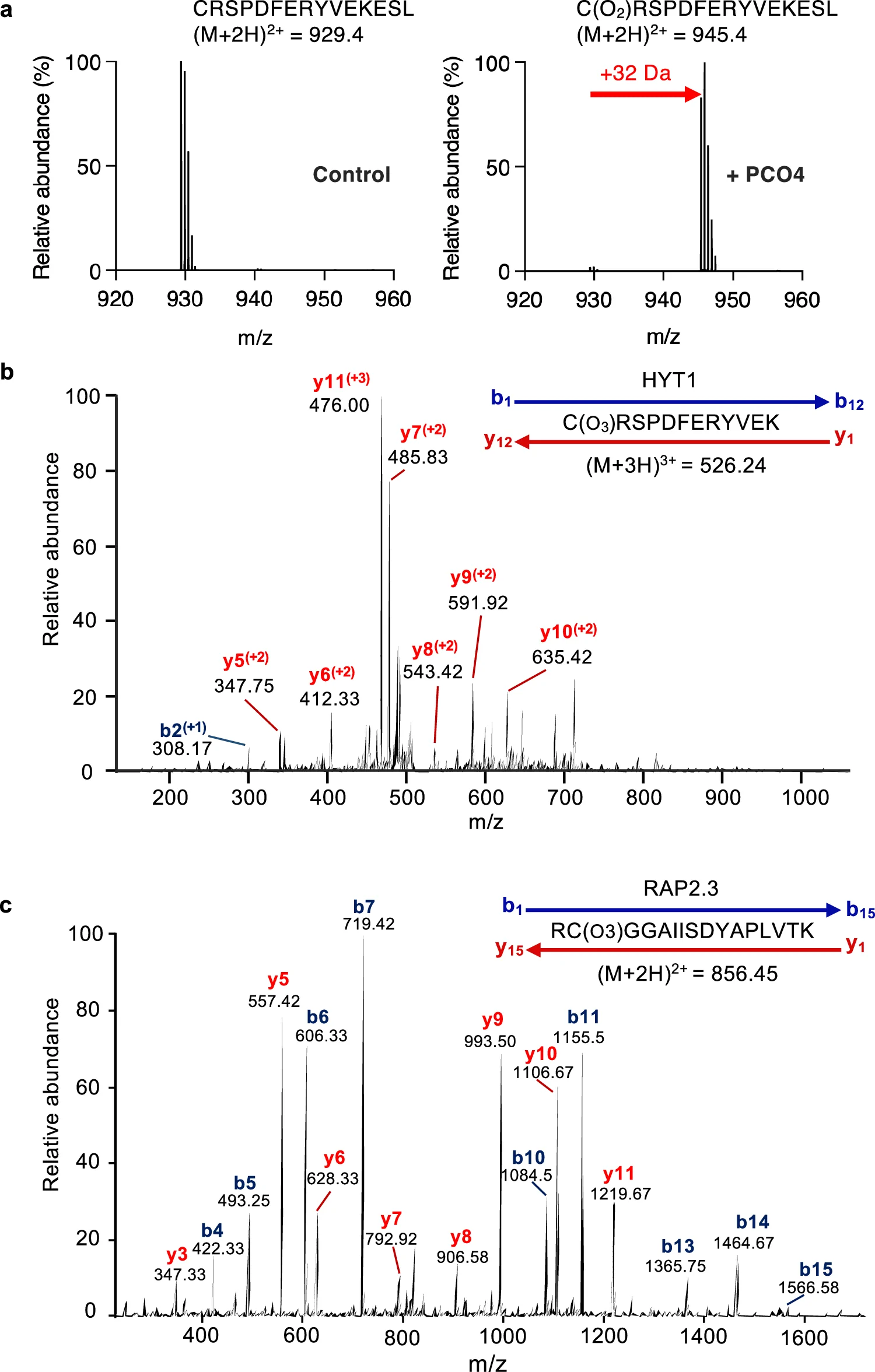
Analysis of the chemical structure at the amino-terminus of HYT1. **a** LC-MS spectra of the HYT1(2-16) peptide in the absence or presence of PCO4 enzyme. A mass increase of +32 Da was observed after incubation with PCO4. **b** MS/MS spectra showing identified b and y-series ions of HYT1 Nt peptide after digestion with LysC ([M + 3H]^3+^ = 526.24) following expression in a wheat germ extract, showing the presence of Cys-sulphonic acid (C3ox) at the amino-terminus. **c** MS/MS spectra of the Nt tryptic peptide of RAP2.3 ([M + 2H]^2+^ = 856.45) following expression in a wheat germ extract, showing the presence of Arginine and Cys-sulphonic acid (R-C3ox) at the amino-terminus, all ions are singly charged.

Because PCO enzymes initiate degradation of substrates by oxidation of Nt-Cys with molecular oxygen, we analysed the capacity of the N-terminus of HYT1 to be enzymatically modified in the presence of molecular oxygen. Firstly, we analysed the activity of PCO4 against a HYT1 2-16 peptide, CRSPDFERYVEKESL (Fig. 2a). After 30 minutes of incubation in aerobic conditions with recombinant PCO4, the synthetic peptide HYT1(2-16) exhibited a mass shift of 32 Da, corresponding to the addition of two oxygen atoms. Comparison of the MS/MS spectra from unoxidised and oxidised peptides confirmed the formation of an N-terminal Cys-sulfinate residue in the latter (Supplementary Fig. 2).

We analysed the status of the N-terminus of the complete protein using the wheat germ-based coupled transcription/translation system, previously shown to contain both PCO and ATE activity but that lacks an active Ubiquitin Proteasome System ^8^. As previously described for RAP2.3 and RAP2.12^8^, affinity purified HYT1 protein showed methionine excision and oxidation of the exposed N-terminal Cys to Cys-sulphonate (SO_3_) at position 2 (Fig. 2b). Interestingly, N-terminal arginylation of HYT1 could not be found. Parallel experiments for RAP2.3 showed both Cys-sulphonate formation and arginylation (Fig. 2c, Supplementary Fig. 2). Additionally, the carbamidomethylated form of the N-terminal Cys was detected, resulting from alkylation of free reduced cysteine by iodoacetamide during sample preparation, indicating that a proportion of the N-terminal Cysteine was still in the reduced and unmodified form in the wheat germ extract (Supplementary Fig. 2).

### The role of HYT1 in development and environmental responses through the PCO N-degron pathway

**Fig. 3:**
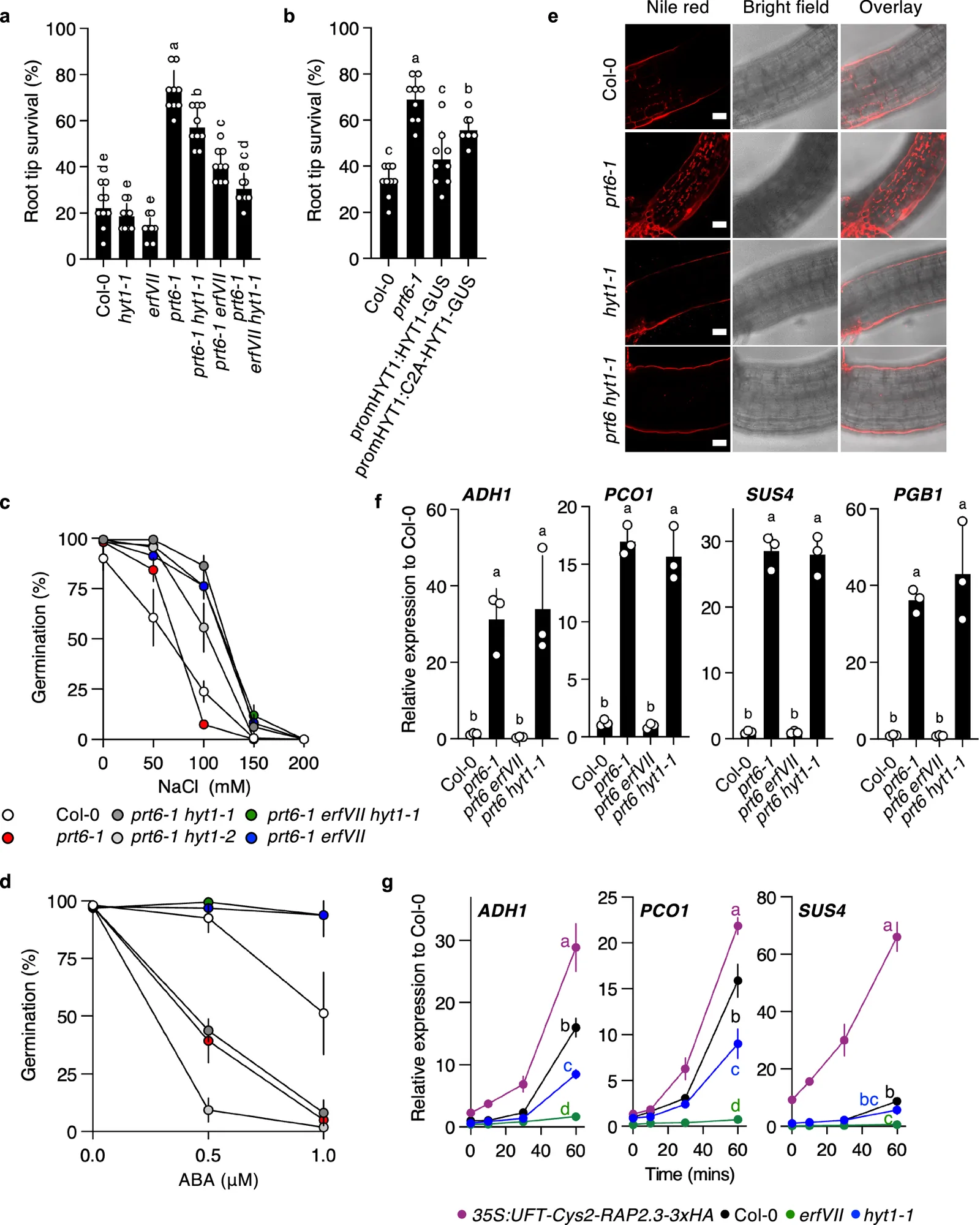
Influence of HYT1 on growth and development and response to environmental stress. **a**,**b** Root tip survival of WT, mutant and transgenic seedlings following 4 h hypoxia ( < 0.5% O_2_) and 3 days recovery. Data are presented as mean ± SD (*n* = 9) significant differences denoted with letters for one-way ANOVA (*p* < 0.05). **c**,**d** Germination of WT and mutant seedlings grown on media containing NaCl or ABA. Data presented as mean ± SD (*n* = 4). **e** Confocal microscopy images of hypocotyl cells (5 days imbibition on media with no added sucrose) stained with Nile Red to reveal red-staining oil bodies. (Scale bar: 50 μm.). **f** Expression of hypoxia-related genes differentially expressed between *prt6-1* and *prt6-1 erfVII* but not between *prt6-1* and *prt6 hyt1-1*. Data are presented as mean ± SD (*n* = 3), significant differences denoted with letters for one-way ANOVA (*p* < 0.05). **g** Expression relative to Col-0 at 0 mins of *PCO1 ADH1* and *SUS4* in response to submergence in the dark (mins), in Col-0, Col-0 (*35SUFT-Cys2-RAP2.3-3xHA*), *hyt1-1* and *erfVII*. Significant differences are denoted with letters for one-way ANOVA (*p* < 0.05) using only the 60 min submerged time point. Data are presented as mean ± SD (*n* = 3).

To determine the genetic importance of *HYT1* as part of the PRT6 N-degron pathway, we analysed phenotypes previously associated with the *prt6-1* loss-of-function allele. For this we utilised two alleles of *HYT1* (SALK_ 052163, *hyt1-1* and GABI_ 809B11, *hyt1-2*, Supplementary Fig. 3a). In both cases expression of *HYT1* was strongly reduced as a result of the T-DNA insertion (Supplementary Fig. 3b). Genetic interactions between *PRT6*, *ERFVII*s and *HYT1* were analysed using *prt6-1 hyt1-1* or *−2* double mutants, and between *ERFVII*s and *HYT1* using *rap2.12 rap2.2 rap2.3 hre1 hre2* (hereafter *erfVII*), *prt6-1 erfVII* and the *prt6-1 erfVII hyt1-1* septuple mutant. Root tip survival has previously been used as an indicator of hypoxia tolerance in response to transient extreme ambient hypoxia (4 h, <0.5% O_2_)^8,18,20^. Whereas *prt6-1* mutants showed strong tolerance to abrupt acute hypoxia, *prt6-1 hyt1-1* showed lower tolerance (Fig. 3a, Supplementary Fig. 4). Hypoxia tolerance of seedling roots of *prt6-1 hyt1-1* was greater than *prt6-1 erfVII*, which was similar to *prt6-1 erfVII hyt1-1*. Whereas *promHYT1:HYT1-GUS* did not increase root tip survival in comparison to Col-0, *promHYT1:Ala2-HYT1-GUS* did (Fig. 3b). Seedling sensitivity to a range of stresses is enhanced by ERFVIIs and repressed through the PRT6 N-degron pathway^11,29,30^. We therefore assessed the role of HYT1 as a component of the PRT6 N-degron pathway in response to seedling stress. Germination of *prt6-1* was sensitive to NaCl, whereas both *prt6-1 hyt1-1* and *−2*, *prt6-1 erfVII* and *prt6-1 erfVII hyt1-1* all showed similar much reduced sensitivity (Fig. 3c). Conversely, ABA repressed *prt6-1* germination, that was similar in *prt6-1 hyt1-1* and more extreme in *prt6-1 hyt1-2* (Fig. 3d). The breakdown of lipid bodies during normal seedling establishment was repressed in *prt6*, but not in *prt6-1 hyt1-1* seedling hypocotyls (Fig. 3e).

We analysed the influence of *HYT1* on seedling global gene expression by comparing transcriptomes of *prt6-1* with *prt6-1 hyt1-1*. All substrates of PRT6 (including HYT1) are stable in *prt6-1* in comparison with *prt6-1 hyt1*, allowing an understanding of *HYT1's* influence on the transcriptome. RNAseq analysis of 7-day old seedlings grown in ambient oxygen with continuous light showed that few genes were strongly differentially expressed (only 25 transcripts are more than 2-fold up-regulated in *prt6-1 hyt1-1* compared to *prt6-1*, and 90 more than 2-fold up-regulated in *prt6-1* compared to *prt6-1 hyt1-1*) (Supplementary Data 2). There was a large overlap in genes both positively and negatively regulated by *ERFVII*s and *HYT1* (Supplementary Fig. 5a) but little overlap between genes regulated by *HYT1* and hypoxia^11^. Comparison of differentially regulated genes with a previously defined conserved set of 49 hypoxia-regulated transcripts^19^ showed that only a small portion were mildly differentially regulated by *HYT1* ( < 2 fold) and most of these were more highly expressed in *prt6-1 hyt1-1* than *prt6-1* (Supplementary Fig. 5b). Analysis of all genes differentially regulated between *prt6-1 hyt1-1* and *prt6-1 erfVII*^8^ revealed very strong up-regulation of hypoxia-regulated transcripts in *prt6-1 hyt1*, indicating that *HYT1* removal does not greatly influence activation of hypoxia genes by ERFVIIs (Fig. 3f, Supplementary Fig. 5b, and Supplementary Data 3), whereas a small set of genes was regulated by *HYT1* but not *ERFVIIs* (Supplementary Fig. 5c and Supplementary Data 2). Whereas there was no difference in expression of hypoxia-related transcripts by 30 min of submergence, following 60 minutes of submergence accumulation of *ADH1* and *PCO1* was reduced in *erfVII* and in *hyt1-1* compared to WT and *35S:UFT-Cys2-RAP2.3-3xHA* (Fig. 3g). Using yeast 2-hybrid analysis, we did not observe direct interactions of any ERFVII with HYT1 (Supplementary Fig. 6).

**Fig. 4:**
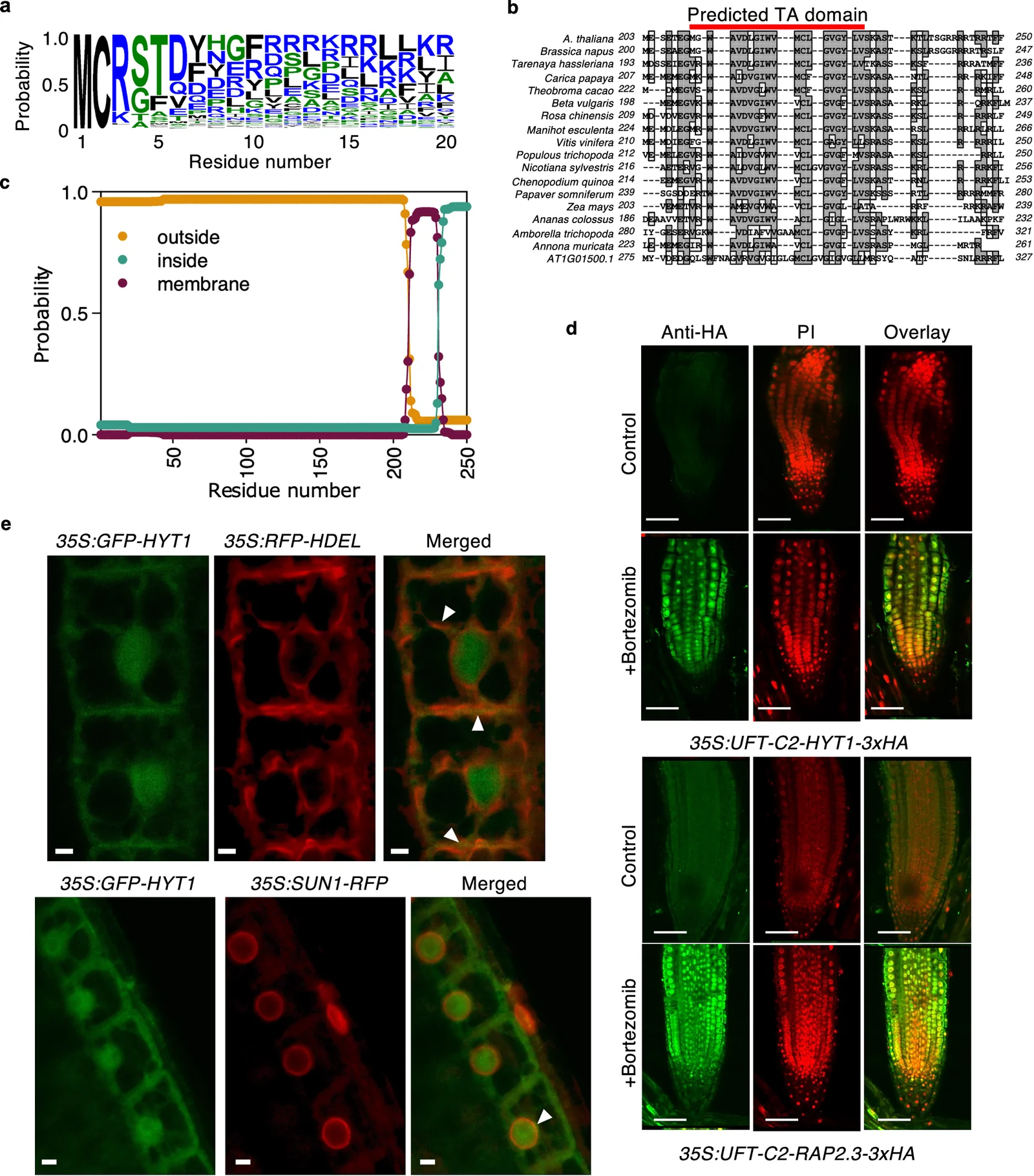
Subcellular localisation of HYT1. **a** Conservation of Cys^2^ in HYT1 similar sequences in angiosperms. The sequence logo represents 147 angiosperm protein sequences (Supplementary Data 2). **b** Comparison of the C-terminal domain of HYT1-like proteins from diverse angiosperm species and AT1G01500.1. The predicted TA domain region is highlighted. Identical residues in dark grey, similar residues in light grey. **c** Prediction of transmembrane helices in HYT1. Transmembrane helices were predicted and visualised using TMHMM − 2.0. The plot shows posterior possibilities of residue location inside, outside or within a membrane. **d** In situ immunolocalisation of HYT1-3xHA and RAP2.3-3xHA in control or bortezomib (50 μM, 3 h) treated roots of 5-day old seedlings, scale bar 50 μm. Counterstaining was done using Propidium Iodide (PI). **e** Colocalization studies with *35S:GFP-HYT1* and *35S:RFP-HDEL* (top row) and *35S:GFP-HYT1* and *35S:SUN1-RFP* (bottom row) for elongating epidermal cells in roots. Merged images show superimposition of GFP and RFP signals, which appear orange for areas of colocalization (arrow). All images shown are single confocal sections. Bars, 4 μm.

To address potential functions and evolution of HYT1, we analysed features of the protein sequence. HYT1 shows strong similarity to a group of proteins conserved only in the angiosperm lineage, where in all cases the N-terminus is completely conserved as Met1-Cys2 (Fig. 4a, Supplementary Fig. 7, Supplementary Data 4). HYT1 is annotated in Araport11 as an NADP + -specific glutamate dehydrogenase, that we conclude is a mis-annotation for the following reasons: It shows only weak similarity to NADP + -specific glutamate dehydrogenases from wide taxonomic groups and does not contain key residues that are part of the Prosite PS00074 consensus active site (Supplementary Fig. 8a, b). Using a C-terminally truncated HYT1 expressed and purified from *E. coli* we could not find either NAD + /NADP+ specific glutamate dehydrogenase or dehydrogenase activity against an extended set of substrates (Supplementary Fig. 8c, d). HYT1 was previously identified as a potential Tail Anchored (TA) protein^31^, and similar sequences from diverse angiosperms all show a clear Tail Anchored C-terminal motif that is highly conserved (Fig. 4b, c Supplementary Fig. 9). Although TA-domain proteins localise to membranes, it was suggested to be localised in the cytoplasm^31^, and subcellular localisation predictor NLStradamus^32^ indicates a conserved nuclear association motif (Supplementary Fig. 10). We analysed the localisation of HYT1 in two ways, firstly by in situ immunolocalization of HYT1-3xHA driven from *35S:UFT-Cys2-HYT1-3xHA* in *A. thaliana* seedling roots. Here HYT1-3xHA was undetectable in control root samples but associated with both cytoplasm and nuclei in the presence of bortezomib, similar to RAP2.3-3xHA (Fig. 4d). Secondly we analysed the sub-cellular location of HYT1 in elongating epidermal cells from 7 d old Arabidopsis roots in *35S:GFP-HYT1* and performed co-localisation analyses using an endoplasmic reticulum (ER) marker RFP-HDEL (*35S:RFP-HDEL*^33^), or nuclear membrane marker SUN1-RFP (*35S:SUN1-RFP*^34^) crossed to *35S:GFP-HYT1* in F1 seedlings (Fig. 4e and Supplementary Fig. 11). GFP-HYT1 (stable due to the presence of N-terminal GFP) was present throughout the ER and nucleus. Co-occurrence of GFP-HYT1 with RFP-HDEL was observed outside the nucleus, and with SUN1-RFP in the nuclear envelope.

### *HYT1* gene expression is activated by ERFVIIs through the PRT6 N-degron pathway

**Fig. 5:**
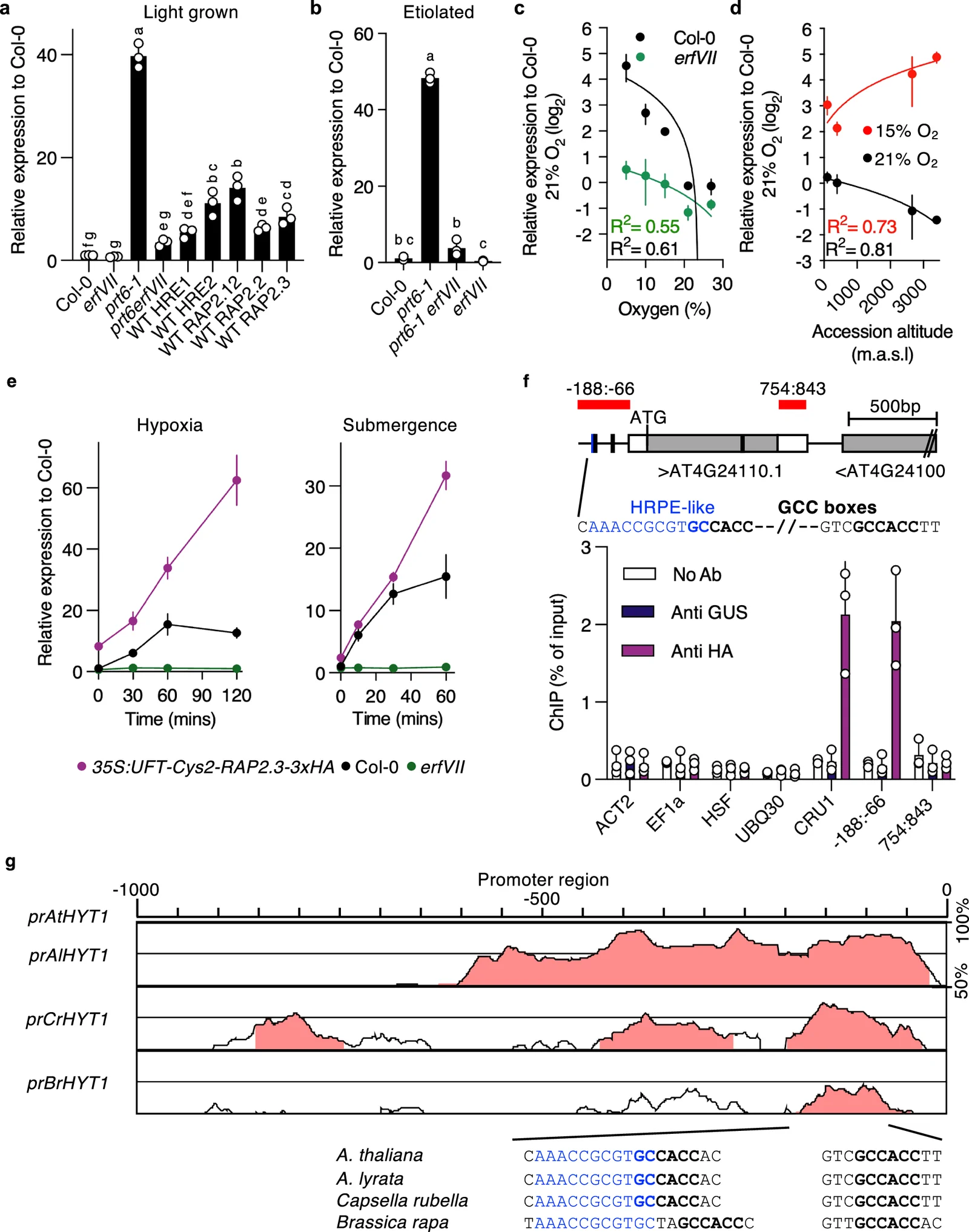
Regulation of HYT1 expression by ERFVIIs through oxygen-sensing. **a** *HYT1* RNA level in WT (Col-0) and mutant seedlings grown in continuous light for 7 days. In each case WT refers to the individual WT ERFVII in the *prt6* background with all other ERFVIIs absent, e.g., WT HRE1 is *prt6-1 HRE1 hre2 rap2.12 rap2.2 rap2.3*. **b** *HYT1* RNA level in WT (Col-0) and mutant 5 day old etiolated seedlings. **c** Influence of ambient O_2_ level on *HYT1* RNA accumulation in WT and *erfVII* seedlings. **d** *HYT1* RNA accumulation in *A. thaliana* accessions collected at different altitudes measured at 48 m a.s.l. (*pO*_*2*_ 21.2) or at 15% ambient O_2_. Accessions used and altitudes are: Col-0 100; Bay-0 400; Sne 2662; Sha 3400 m a.s.l. **e** *HYT1* RNA accumulation in seedlings grown in continuous light for 7 days following imposition of hypoxia or submergence treatments. **f** Schematic of the *HYT1* gene showing potential GCC (bold) and HRPE (blue) like *cis*-elements, white boxes 5’ and 3’ UTRs, grey box protein coding region. Direction of genes are indicated by ><. ChIP analysis of HRE2-3xHA occupancy of *HYT1* gene regions (and known positive and negative sequences^40,54^) using anti-HA, and no or GUS antibodies. **g** Peaks and valleys identity plot (50–100%) showing pairwise alignments of −1000 region of HYT1 like genes to promoter (*pr*)*AtHYT1* from *A. lyrata* (*prAlHYT1*), *C. rubella* (*prCrHYT1*) and *B. rapa* (*prBrHYT1*). Conserved non-coding sequences are indicated in pink. All experiments were carried out using 5 day old etiolated seedlings at *pO*_*2*_ 21.2 (48 m a.s.l.) unless otherwise stated. Means are plotted, ( ± SD), significant differences denoted with letters for one-way ANOVA (*p* < 0.05), coefficient of determination (R^2^) is given. For each *n* = 3 biologically independent experiments.

*HYT1* transcript abundance was previously shown to increase in response to hypoxia^26^. This suggested that *HYT1* gene expression may be controlled by ERFVIIs. In light-grown seedlings, *HYT1* transcript levels were very low and similar in *erfVII* and WT but higher in *prt6-1* than *prt6-1 erfVII* (Fig. 5a), indicating that *HYT1* is regulated by *PRT6* through *ERFVII*s. Individually, *RAP2.12*, *RAP2.3* and *HRE2* showed the greatest capacity to enhance *HYT1* expression in a *prt6-1* background in which all other ERFVIIs are absent, but not as great as in the presence of all stabilised ERFVIIs (*prt6-1*). A similar ERFVII dependent regulation of HYT1 was also observed in etiolated seedlings grown in continuous dark (Fig. 5b). The regulation of *HYT1* by ERFVIIs occurs through oxygen-sensing, as expression in etiolated seedlings increased with decreasing ambient oxygen level (from 27-5%) in WT, but not in the *erfVII* mutant where it remained low irrespective of oxygen level (Fig. 5c). Oxygen sensing was shown to influence adaptation to altitude^35^, so we analysed the relative expression of *HYT1* transcript in accessions of *A. thaliana* collected at increasing altitude (representing *pO*_*2*_ from 21-14 kPa). At 21 kPa ambient O_2_ *HYT1* expression was lower in accessions from higher altitudes, but this difference was reversed at 15% ambient O_2_ (Fig. 5d). *HYT1* expression increased rapidly after the initiation of either ambient hypoxia or submergence treatment (Fig. 5e). This increase was enhanced in the *35S:UFT-Cys2-RAP2.3-3xHA* transgenic background but was abolished in *erfVII*. A search for ERFVII GCC and HRPE like *cis*-elements in the HYT1 gene region revealed the presence of non-canonical sequences just upstream of the ATG (Fig. 5f). We analysed the occupancy of this region in Col-0 by the ERFVII HRE2-3xHA (from *35S:HRE2-3xHA*^15,35^) in comparison to the HYT1 3’UTR that does not contain equivalent sequences, and to known negative and positive (*CRU*) genomic regions^35^ (Fig. 5f). Occupancy was high and at equivalent levels in *CRU* and the *HYT1* upstream region compared to negative controls and the *HYT1* 3’UTR region. Phylogenetic shadowing was used to investigate the evolutionary conservation of these motifs, as has previously been done for HRPE elements in the hypoxia-responsive *LDB41* gene^36^. In comparison to the *A. thaliana HYT1* promoter, scanning of the −1000 regions of *HYT1*-like genes from other Brassicaceae species (*A. lyrata*, *Capsella rubella* and *Brassica rapa*) revealed the region proximal to the ATG, previously identified as containing HRPE and GCC boxes in *AtHYT1*, as being highly conserved (Fig. 5g). Both HRPE-and GCC box-like sequences were conserved in all four genomic sequences.

## Discussion

Oxygen sensing is key to plant environment interactions and to plant growth and development^2,3,37^. Although enzymes of the associated PCO N-degron pathway are highly conserved throughout eukaryotes, substrates are not; instead, they are taxonomically restricted at different levels and rarely found^25^. Here we identify a novel substrate of the pathway, HYT1, previously identified as a hypoxia up-regulated transcript^26^. HYT1 represents a plant substrate of this oxygen-sensing pathway that is not directly associated with gene transcriptional control, and in this way is similar to the identified animal ADO substrates^3^. Like known plant substrates VRN2 and ZPR2, HYT1 is conserved as a Met-Cys initiating protein in angiosperms, indicating that it plays an important role in development and/or responses to the environment. Arg-3 is also highly conserved in HYT1-like proteins, similar to VRN2^18^. HYT1 displays typical characteristics of PCO/ADO substrates, including oxygen-and NO-dependent degradation that requires the E3 ligase PRT6 and ARGINYL TRANSFERASEs, and whose degradation is enhanced by BIG, as was recently also found for ERFVIIs and VRN2^6^. Previously, it was observed that HYT1 can be ubiquitylated in vivo at Lys-73^38^. When constitutively expressed, HYT1 is rapidly stabilised during submergence or ambient acute hypoxia, with similar kinetics to RAP2.3^8^, which reflects the rapid reduction in intracellular oxygen levels in response to these treatments. Return to normoxia resulted in a much slower destabilisation of HYT1 compared to RAP2.3^8^, which may reflect the localisation of HYT1 in cellular regions less accessible to PCO- N-degron pathway enzymology, or association with other cellular components that restrict access to the N-terminus.

PCO4 was able to oxidise the N-terminal Cys of HYT1 to Cys-sulfinic acid (Cys-SO_2_H), indicating that it is an effective substrate, and following incubation in a plant extract, the N-terminus of HYT1 was shown to be modified by oxidation of Cys to Cys-sulfonic acid (Cys-SO_3_H). Cys-oxidation to sulphonic acid was also shown to occur in RAP2.3 and RAP2.12^8^, and also in the mammalian Cys-2 protein RGS4 purified from mouse L cells^9^, indicating that in addition to PCO/ADO action (that adds two oxygen atoms to N-terminal Cys) it is possible that a monooxygenase is also involved in the pathway to add the third oxygen. It is unclear why we could not observe arginylation of the N-terminal Cys of HYT1 by the plant extract, which might suggest that the N-terminus of HYT1 is a weak ATE substrate. This may be due to steric effects of residues closely associated with N-terminal Cys or other structural features of the protein.

Genetic analysis of *HYT1* function in plant growth and development indicated that it participates in a subset of functions that have been ascribed to the ERVIIs. HYT1 activity was required for root tip survival of acute hypoxia, where removal of *HYT1* in the *prt6-1* background (*prt6-1 hyt1-1*) resulted in reduced survival compared to *prt6-1*. Whereas *prt6-1* seedling germination was hypersensitive to high salt (that stabilises HYT1), both *prt6-1 hyt1* and *prt6-1 erfVII* mutants were less sensitive. Similarly, whereas oil body breakdown was inhibited in *prt6-1* seedling establishment, this was not the case in *prt6-1 hyt1*. In contrast, the insensitivity of seedling establishment to ABA shown by *prt6-1 erfVII* was not shown by *prt6-1 hyt1*. In all cases, the *prt6-1 erfVII hyt1-1* mutant behaved in a similar way to *prt6-1 erfVII*, indicating that *HYT1* action is downstream of *ERFVII*s, which is consistent with our discovery that *HYT1* is regulated by ERFVIIs. Because all known PCO N-degron pathway substrates in plants are associated with gene transcription, we investigated the influence of HYT1 on genome expression, and found that few genes were differentially expressed between *prt6-1* and *prt6-1 hyt1-1*, and these did not include hypoxia-regulated transcripts^26^. Although expression of members of the core 49 hypoxia up-regulated genes were not differentially regulated by removal of *HYT1* in a *prt6-1* background, their expression was reduced in *hyt1-1* during seedling response to submergence. This might indicate that HYT1 has a role in regulating the kinetics of their accumulation, perhaps by influencing mRNA transport from the nucleus or other processes related to transcript stability. The fact that HYT1 does not strongly influence hypoxia/ERFVII-regulated gene expression indicates that it does not affect ERFVII function, and we found that HYT1 does not directly interact with ERFVIIs. HYT1 was previously identified as a Tail-Anchored protein, not associated with the chloroplast membrane^31,39^. *A. thaliana* TA-domain proteins are targeted to many intracellular membranes. We could show that HYT1 was co-localised with ER and inner nuclear membrane markers, suggesting that its location is associated with these membranes. In addition, it was also found inside the nucleus, indicating that it also functions away from membrane interfaces. Whereas all known plant PCO N-degron pathway substrates are nuclear localised, in animals they are all cytoplasmic^3^. HYT1 is therefore unique in being found in several compartments. Although annotated as a NADP + -specific glutamate dehydrogenase, we found no evidence of dehydrogenase activity with purified HYT1 and note a lack of any Rossmann fold-like structures (that might indicate NAD + /NADP+ binding) in available structural predictions. Therefore, the exact biochemical function of HYT1 remains to be determined.

*HYT1* is a member of the hypoxia core 49 conserved genes up-regulated in response to hypoxia, and its expression is regulated through *ERVFII*s^8^. Driven by its own promoter HYT1 did not accumulate under normoxic conditions, though accumulation of Ala2-HYT1 demonstrated that *HYT1* RNA is present in shoot and root apical meristem regions. In response to submergence and soil waterlogging, HYT1 accumulated in primary and lateral root tissues. We found that the ERFVII HRE2 associates with conserved cis-elements in the promoter of *HYT1* in vivo, confirming previous ChIPseq data^40^. *HYT1* expression required non-autonomous *ERFVII* activity (as do other hypoxia-regulated transcripts^8^) and was controlled by oxygen-sensing through *ERFVII*s. The abundance of ERFVII proteins was previously also shown to be regulated by altitude adaptation (relatively lower abundance in higher altitude accessions at higher ambient oxygen levels), and consistent with this, here we observed lower expression of HYT1 at high oxygen in accessions from higher altitudes, that was increased at lower oxygen levels, indicating that HYT1 transcripts are controlled by ERFVIIs through altitude adaptation. HYT1 accumulation is strongly associated with the root system, however, unlike ZPR2 and VRN2, HYT1 is only stabilised in root tissues under acute hypoxia, including waterlogging in soil.

**Fig. 6:**
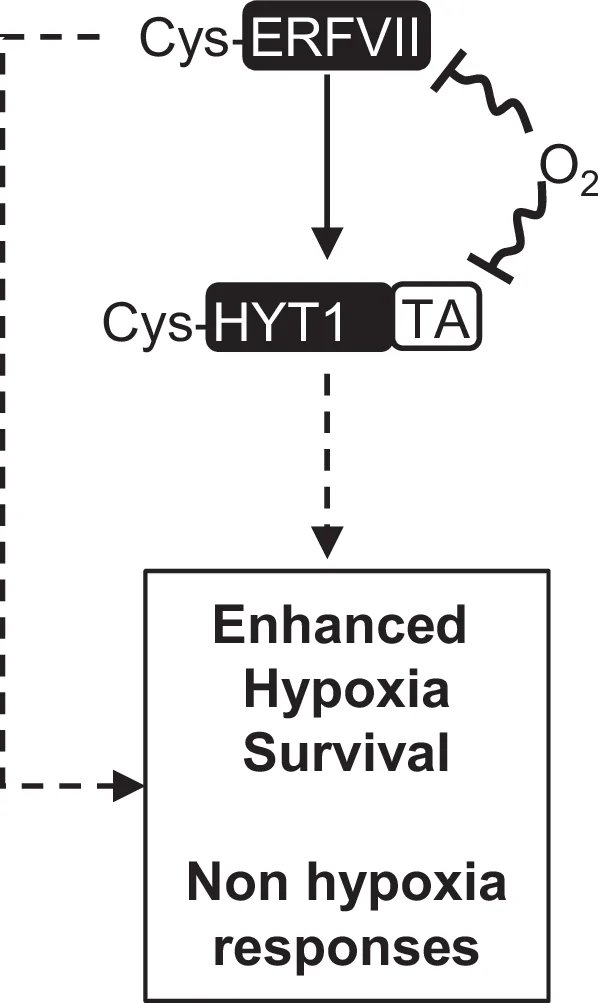
Model showing the relationship between ERFVIIs, HYT1 and oxygen, forming a coherent feed-forward loop. Proteins are indicated as ovals; TA, tail-anchored domain; solid lines indicate direct regulation, broken lines indicate indirect regulation. Inhibition of ERFVIIs and HYT1 by oxygen is indicated by a wavy line with a blocked end; Cys, N-terminal cysteine. Biological model shown in Supplementary Fig. 12.

Why is HYT1 protein stability directly regulated by oxygen and transcriptionally by ERFVIIs? The relationship between ERFVIIs, HYT1 and downstream gene expression and physiological responses has properties of a coherent feed-forward loop^41^, but with a single inducer for both factors (hypoxia) (Fig. 6, Supplementary Fig. 12). This implies that responses are dependent on reduced oxygen as a signal to initiate the loop, and that the response time of the loop is delayed in an inducer (hypoxia)-sensitive manner in comparison to simple regulation solely through ERFVIIs. It also implies that the response only occurs to persistent stimulation and not to short duration hypoxia^41^. Both of these features would be consistent with the idea that short-duration (perhaps stochastic) acute hypoxia experienced by tissues does not trigger a response, which only occurs with persistent low ambient oxygen. This would provide a check to gate the dramatic changes in cellular biology required to endure externally-imposed hypoxia. Accumulation of HYT1 in response to soil waterlogging may corroborate this hypothesis, as no accumulation was observed in roots of control soil samples, where it is very likely that transient hypoxia is encountered as roots grow through the soil.

Only 4 types of PCO substrate transducers have thus far been identified in *A. thaliana* from a potential of around 250 Met1-Cys2 initiating proteins. Only a small subset of these will be substrates because of the constraints of N-degron structure^42^. Defining the complete set of true substrates will greatly enhance understanding of the biochemical diversity of functions associated with transduction of the low oxygen signal as a consequence of acute and chronic low oxygen environments. Here we introduce HYT1 as a conserved root-associated substrate of the oxygen-sensing PCO N-degron pathway and show that its stability is directly regulated by oxygen/NO and transcriptionally through ERFVIIs, contributing to key ERFVII-regulated phenotypes.

## Methods

### Plant material, analysis of plant growth and response to stress

The mutant alleles *big-*2 (SALK_045560), *hyt1-1* (SALK_052163) and *hyt1-2* (GABI_809B11) were obtained from NASC (University of Nottingham, UK). Combination mutants and other transgenics were obtained by crossing with previously described mutants *nia1 nia2*^28^, *prt6-1*^29^, *prt6-1 erfVII*^35^, *ate1 ate2*^29^. Plants were grown in controlled environment conditions as previously described^43^. Seedling root-tip hypoxia ( < 0.5% ambient oxygen) survival assays and submergence assays were carried out as previously described^8^. Seedling establishment assays for ABA and salt tolerance and recovery from high salt were carried out by supplementing half-strength MS media with ABA or NaCl at the indicated concentrations^29,30^. Oil bodies were visualised in 5-day-old seedlings at the root-hypocotyl junction as previously described^29^. Histochemical staining for GUS activity was carried out as previously described^15^. Nineteen-day-old plants (grown under 16 h light; 8 h dark) grown in mixed sandy-clay loam soil were waterlogged by submerging pots up to the base of plant stems in water as described^8^.

### Generation of constructs and plant/leaf transformation

*promHYT1:HYT-GUS* was obtained by cloning 2 kbp of the upstream promoter and the entire gene region excluding the final stop codon into pGWB433^44^. *35S:UFT-HYT1-3xHA* was cloned as previously described using the CDS of HYT1 obtained from seedling cDNA^8^. HYT1 (AT4G24110) CDS was cloned into pDONR™/Zeo using the primers attB1 HYT1 and attB2 HYT1, and transferred into pGWB6 to create a GFP N-terminal fusion protein. The construct was transformed into Arabidopsis using standard protocols^45^.

### Analysis of GFP-HYT1 subcellular localisation

Confocal imaging was performed using an inverted Leica True Confocal Scanning (TCS) SP8 microscope, and images were analysed with Leica Application Suite X (LAS X) software. Roots of 6-7 days old seedlings were imaged with the HC PL APO CS2 20x/0.75 DRY objective. Fluorescence was detected using PMT Detectors with detection bands optimised for GFP (500-531 nm) and RFP (580–650 nm) to minimise crosstalk. In order to quantify the level of co-localisation between GFP and RFP, we use FIJI (ImageJ) of confocal images using BIOP’s (Bioimaging and Optics Platform) version of JACoP (Just Another Colocalization Plugin). Relevant Measurements include Manders’ Colocalization or Overlap Coefficient in the selected ROI (Region Of Interest). Manders’ Colocalization Coefficients (MCC)^46^ measured co-occurrence (or colocalization in this case) of GFP fluorescence and RFP fluorescence independent of signal proportionality. Manual threshold values were chosen for red and green channels, respectively.

### Bioinformatic analyses

Comparison of the N-terminus of angiosperm HYT1-like proteins was carried out following BLAST identification at NCBI of angiosperm (taxid:3398) proteins (refseq_protein) similar (expected threshold 0.0000005) to AtHYT1, which identified 147 Met-Cys initiating sequences representing 115 species (Supplementary Data 2). The first 20 residues of proteins were compared using Weblogo^47^. Full-length proteins were compared using default settings of MUSCLE multiple sequence alignment in MacVector v18 (MacVector Inc, Cambridge UK). Analysis of evolutionary conservation within the −1000 region of HYT1-like genes was carried out as previously described^36^. The −1000 DNA sequence from the initiating ATG was obtained from ENSEMBL plants (https://plants.ensembl.org/index.html). These were compared through pairwise alignments using mVISTA^48^ and the alignment programme LAGAN^49^. Promoter sequence similarities were confirmed using DALIGN^49^. PROSITE documentation PDOC00071 (for PROSITE entry PS00074) Glu / Leu / Phe / Val dehydrogenases active site was obtained from https://prosite.expasy.org/cgi-bin/prosite/nicedoc.pl?PS00074. Prediction and visualisation of transmembrane helices in HYT1 and similar proteins was carried out using TMHMM − 2.0^50^. Prediction of nuclear localisation signal sequence in HU40 protein was carried out using NLStradamus with default parameters (http://www.moseslab.csb.utoronto.ca/NLStradamus/). Four set proportional Venn diagrams were produced using DeepVenn^51^.

### Immunolocalisation, protein extraction, Western blotting and Chromatin Immunoprecipitation

Immunolocalisation, protein extraction, Western blotting (mouse anti-HA primary antibody; Sigma-Aldrich #H3663), and Chromosome Immunoprecipitation (ChIP), hypoxia, submergence and cPTIO treatments were carried out as previously described^8,35^.

### Seedling transcriptome analysis and quantification of transcript expression

RNAseq was carried out as previously described using 7-day old seedlings grown on 1/2 strength MS agar in continuous light^8^ and is a part of the same experiment as GEO Sample GSM7029623. RT-qPCR was carried out as previously described^8^.

### Yeast two-hybrid analysis

Met-Ala versions of coding regions excluding the stop codon of HYT1 and all five ERFVIIs were cloned into pENTR^TM^/D-TOPO^TM^ (Thermo Fisher Scientific) entry vector. HYT1 and ERFVIIs entry vectors were mobilised into Gateway compatible DNA binding domain containing pDEST32 (BD) and activation domain containing pDEST22 (AD) vectors (ProQuestTM Two-Hybrid System, Invitrogen), respectively. These constructs were co-transformed into the Mav203 yeast strain using the EZ-yeast transformation kit (MP Biomedicals). Transactivation activity of the pDEST32:HYT1 construct was assessed by spotting colonies on -HT (SD/-His /-Trp) supplemented with 3-amino-1,2,4-triazole (0, 10, 25, 50, 75 and 100 mM 3-AT) dropout media and incubated at 30 C for 2 days to avoid false positive results. Media supplemented with -HT + 75 mM 3-AT (TDO) was used to assess interactions. For one-to-one interactions, pDEST32:HYT1 was co-transformed with each of the pDEST22:ERFVII constructs, and colonies were spotted onto drop-out media -LW (SD/-Leu/-Trp), - HLW (SD/-His/-Leu/-Trp), -HLW + 50 mM 3-AT and -HLW + 75 mM 3-AT. Co-transformed pEXP32/Krev1 + pEXP22/RalGDS-wt and pEXP32/Krev1 + pEXP22/RalGDS-m1 were used as positive and negative controls, respectively.

### Analysis of the chemical structure of HYT1 N-terminus in vitro

Analysis of the stability of HYT1-3xHA in vitro was carried out using a rabbit reticulocyte lysate as previously described^15^, containing plasmid template pUFT-Cys2-HYT1-3xHA.

PCO4 was purified as previously described^52^ and the HYT1 2-16 peptide, CRSPDFERYVEKESL, was synthesised using a CEM Liberty Blue 2.0 peptide synthesiser. General synthetic procedures for Fmoc deprotection and capping were carried out in accordance with the manufacturer’s specifications. Amide coupling was performed for 2.5 min at 90 °C under microwave irradiation in the presence of amino acid (0.2 M in DMF, 4 eq.), Oxyma Pure (1 M in DMF, 4 eq.) and di*iso*propylcarbodiimide (1 M in DMF, 4 eq.). The peptide was cleaved from the resin using a cocktail of trifluoroacetic acid, thioanisole, tri*iso*propylsilane, and water (90:4:4:2) and precipitated in cold diethyl ether. The crude material was purified on a Waters 1525 binary pump HPLC equipped with a dual wavelength UV detector set to 210 nm and 280 nm using a Waters Sunfire 5 μm 19 ×150 mm (C-18) preparative column operating at a flow rate of 6 mL/min. A mobile phase consisting of 0.1% trifluoroacetic acid in water (Solvent A) and 0.1% trifluoroacetic acid in acetonitrile (Solvent B) was used with an appropriate gradient. Purified material was analysed by high-resolution mass spectrometry using a Bruker micrOTOF Focus II by Electrospray Ionisation (ESI) operating in positive ionisation mode. Peptide purity was quantified using a Thermo Scientific Ultimate 3000 μHPLC system equipped with a photodiode array detector (λ = 210, 254, 280, and 400 nm) using a Waters SunFire 5 µm, 2.1 × 150 mm column (C-18) at a flow rate of 0.6 mL/min on a 10–50% Solvent B gradient over 10 min (using the same solvent systems as preparative HPLC).

Analysis of PCO4 oxidation of HYT1(2-16) was carried out using 200 µM of HYT2-16 peptide and 2 µM PCO4 enzyme at 25˚C under aerobic conditions. Activity buffer consisted of 250 mM NaCl, 50 mM tris-HCl 7.5 pH 10 mM DTT. The reaction was quenched with 1 v HPLC gradient grade methanol, centrifuge 2 min at 5000 g and supernatant transferred to a new tube. For LC-MS analysis, 2 µl of 1:20 dilution of the reaction was used. Analysis of the N-terminus of HYT1 following incubation in a wheat germ extract was carried out as previously described, containing plasmid template pTNT-HYT1-3xHA^8^. Protein-coupled in vitro transcription/translation was carried out using a TnT® SP6 High-Yield Wheat Germ Protein Expression System (HYWGE) (Promega, USA) according to the manufacturer’s recommendations. The template used was pTNT-HYT1-3xHA. Each reaction contained 30 μL HYWGE with 20 μL of 300 ng/μL pTNT-HYT13xHA construct purified by QIAprep Spin MiniprepKit (QIAgen, Crawley, UK). The reaction mixtures were incubated for 2 h at 25 °C. Three reactions of TnT® SP6 High-Yield Wheat Germ Protein Expression System (HYWGE) (Promega, USA) were added separately to 50 μL of Pierce™ Anti-HA Magnetic Beads (Thermo Fisher, UK), and each topped up with 700 μL of freshly filtered (0.2 μm pore size) 1X Tris-Buffered Saline (TBS; 25 mM Tris-HCl pH 7.2, 0.15 M NaCl). Samples were placed on a rotary wheel at 10 rpm. After 30 min samples were placed on a magnetic stand, the supernatant was removed, and beads were washed 3 times with 700 μL TBS with 0.1 % Tween 20 (Fisher Bioreagents, UK). Before elution, beads were washed with autoclaved molecular-grade water. Elution of the beads was achieved by adding 30 μL of Pierce™ Lane Marker Non-Reducing Sample Buffer (2X) (Thermo Fisher, UK) to the tube containing beads from the first reaction, followed by incubation at 96 °C for 10 min. Following precipitation, the supernatant was removed and transferred to the tube containing beads from the second reaction and the heating and precipitation steps were repeated. Finally, the supernatant was transferred to the third tube, and after heating and precipitation, the supernatant was once again recovered and 1.5 μL DTT 1 M was added before loading onto an SDS-PAGE gel. Samples from bead elution were loaded onto a 1.5 mm wide 12% gel SDS-PAGE gel. Gels were run for 20 min at 80 V followed by 100–120 min at 120 V. Staining was carried out in 25 mL of Coomassie InstantBlue™ Protein Stain (Exepedeon, UK) for 1 h on an orbital shaker with rotation at 50 rpm. Gels were washed with Milli-Q water (3x) and left overnight in water for de-staining. The band corresponding to the purified HYT1 was excised with a scalpel and cut into 1mm^3^ pieces.

In gel protein digestion: Gel fragments were destained in 50 μL 50% acetonitrile (MeCN) (Fisher Scientific, UK) AND 50% 100 mM Ammonium Bicarbonate (AmBic) (Sigma Aldrich) for 10 min. Gel pieces were then dehydrated with 450 μL 100% MeCN with vortexing for 3 min (dehydration step). The supernatant was discarded, and gel pieces were reduced with 50 μL 10 mM DTT (Sigma Aldrich) dissolved in 100 mM AmBic, and incubated at 55 °C for 30 min in the dark. The dehydration step was repeated, and gel pieces were incubated with 50 μL of 10 mM iodoacetamide (IAA) (Sigma Aldrich) in 100 mM AmBic for 30 min in the dark followed by another dehydration step. After that, 50 μL containing LysC (10 ng/μL) or Trypsin (10 ng/μl) in 50 mM AmBic was added to the gel pieces and incubated at 37 °C overnight (18 hours). After digestion, the reaction was quenched with 1 μL formic acid (Fisher Scientific, UK). Samples were vortexed, centrifuged and the supernatant was moved to a tapered plastic LC vial (ThermoFisher, UK).

### LC-MS and MS/MS analysis of protein digests

Digests were analysed on a Dionex U3000 nanoLC coupled to a ThermoFisher LTQ FT Ultra mass spectrometer (a hybrid linear ion trap-Fourier transform ion cyclotron resonance (FTICR) instrument equipped with a 7 Tesla magnet). Samples were loaded onto a C18 Pepmap300 cartridge (10 mm, 300 Å, 5 μm particle size) and separated using a Thermo Scientific C18 Pepmap300 column (150 mm×75 μm, 300 Å, 5 μm particle size) attached to a 30 μm Picotip emitter (NewObjective) in the nanoESI MS source. The mobile phases A and B consisted of 95:5 water:MeCN (v/v) and 5:95 water:MeCN (v/v), respectively, and both contained 0.1% formic acid.

Samples (5 µL) were injected in load-trapping mode. Peptides were eluted using a 30 min linear gradient of mobile phase B from 0 to 55% followed by 5 min at 90% B and 15 min column re-equilibration. The LTQ FT Ultra mass spectrometer was equipped with a standard ThermoFisher nanoESI source through which a 1.8 kV voltage was applied to the Picotip emitter. The inlet capillary of the mass spectrometer was held at 275 °C with a tube lens value of 145 V.

For the identification of peptides, the mass spectrometer was operated in data dependent acquisition (DDA) mode on the five most intense ions per survey scan. After identification of the different N-terminal modifications, the mass spectrometer was operated with a targeted run mode. Precursor ions (*m/z* = 526.24 for Cys-sulphonic acid and *m/z* = 529.24 for Cys-alkylation) were selected for MS/MS. Ions were isolated in the linear ion trap within a window of *m/z* = 2 and subjected to a relative collision energy of 35.

For the PCO4 activity assay, the mass spectrometer was operated in data-dependent acquisition (DDA) mode on the five most intense ions per each survey scan.

All data was analysed manually using Xcalibur software (ThermoFischer). Predicted peptides of interest masses and fragmented ion masses were calculated with https://db.systemsbiology.net/proteomicsToolkit/

### Assay for HYT1 potential enzyme activity

Assay for NAD + /NADP+ glutamate dehydrogenase activity was carried out as previously described by assessing increased absorbance at 340 nm due to reduction of NAD + /NADP + ^53^. HYT1 was cloned without the TA domains (HYT1ΔTA) as follows: the first 615 bp of the HYT1 CDS (AT4G24110) were codon optimised and synthesised using Genewiz service (Azenta Life Science) with EcoRV and EcoRI flanking sites. The optimised CDS was digested and ligated into a pMAL6CT vector using EcoRI and EcoRV restriction enzymes. Vector was transformed into *E. coli* DH5alpha cells for sequence confirmation and into Rosetta cells for protein induction (Novagen 70954). HYT1ΔTA was expressed as a His-MBP fusion protein (pMAL6CT-HYT1-ΔTAdomain). Overnight pre-cultures were diluted 1:100 into LB supplemented with 0.2% glucose and 100 µg/ml ampicillin and grown at 37 °C with shaking until OD₆₀₀ 0.6–0.8. Protein expression was induced with 0.3 mM IPTG for 4 h at 28 °C. Cells were harvested by centrifugation (4000 × g, 10 min, 4 °C) and pellets stored at −80 °C. Cell pellets were resuspended in extraction buffer (20 mM sodium phosphate pH 7.4, 500 mM NaCl, 10 mM imidazole, 1 mM PMSF, 0.5 mg/ml lysozyme) and lysed by sonication. Clarified lysate (15,000 × g, 30 min, 4 °C) was loaded onto a HisTrap column (Cytiva) equilibrated in binding buffer (20 mM sodium phosphate, 20 mM imidazole). The column was washed with 40 mM imidazole and the fusion protein eluted with 500 mM imidazole. After buffer exchange into TEV cleavage buffer (50 mM Tris-HCl pH 7.5, 150 mM NaCl, 1 mM DTT), the His-MBP tag was removed by overnight incubation with TEV protease (1:100 molar ratio, 4 °C rotary mixer). Cleaved tag and TEV protease were removed by subtractive Ni-NTA chromatography, collecting the unbound flowthrough. Purified HYT1 was buffer-exchanged into storage buffer (50 mM Tris-HCl pH 7.5, 150 mM NaCl, 10% glycerol, 1 mM DTT) using PD-10 desalting columns (Cytiva), snap-frozen in liquid nitrogen and stored at −80 °C. To investigate a possible GDH function for HYT1 we performed spectrophotometric assays where the activity was determined by measuring the increase in Absorbance at 340 nm, corresponding to the production of NAD + /NADP + . We used as a positive control the commercially available L-Glutamic Dehydrogenase from bovine liver Type II (Sigma Aldrich G2626). Enzymatic assays were performed in a total volume of 150 ul in a 96 well plate. The buffer contained 0.05 M sodium pyrophosphate (pH 9.2), 4.3 mM NAD+ or NADP+ as cofactors and 130 mM glutamic acid as substrate. Absorbance at 340 nm was measured using the Microplate Reader FluorStar Omega (BMG LABTECH) at a constant temperature of 25 C. An appropriate amount of protein/enzyme was added before the start of the reaction, and Abs 340 nm was measured every minute for 30 min.

### Statistics and reproducibility

No statistical methods were used in predetermining sample size, and experiments were not randomised. Investigators were not blinded to allocation during experiments and outcome assessment. For experimental analyses, at least three independent replicates with different original biological material are reported for each experiment, and each experiment was repeated at least twice unless otherwise stated. All graphs were produced using Prism GraphPad software (Version 11), which was also used to calculate mean, standard deviation and one-way ANOVAs.

### Reporting summary

Further information on research design is available in the Nature Portfolio Reporting Summary linked to this article.

## Supplementary information

Supplementary Information

Description of Additional Supplementary Files

Supplementary Data 1

Supplementary Data 2

Supplementary Data 3

Supplementary Data 4

Supplementary Data 5

Reporting Summary

Transparent Peer Review file

## Source data

Source Data

## Data Availability

The RNAseq data generated in this study have been deposited in the GEO database under accession code GSE261364. The mass spectrometry proteomics data used in this study are available at PRIDE under accession code PXD083373 and at the University of Nottingham Repository at https://repository.nottingham.ac.uk/handle/123456789/77206. Source data are provided with this paper.
