# Supplementary material for: Hierarchical regulation of oxygen-sensing through coupled transducers in Arabidopsis thaliana
Source: Nat Commun. 2026 Sep 16;17:10861. doi: 10.1038/s41467-026-77710-x (PMC13672779; doi:10.1038/s41467-026-77710-x)
Supplement: Supplementary file 1 [file 41467_2026_77710_MOESM1_ESM.pdf]

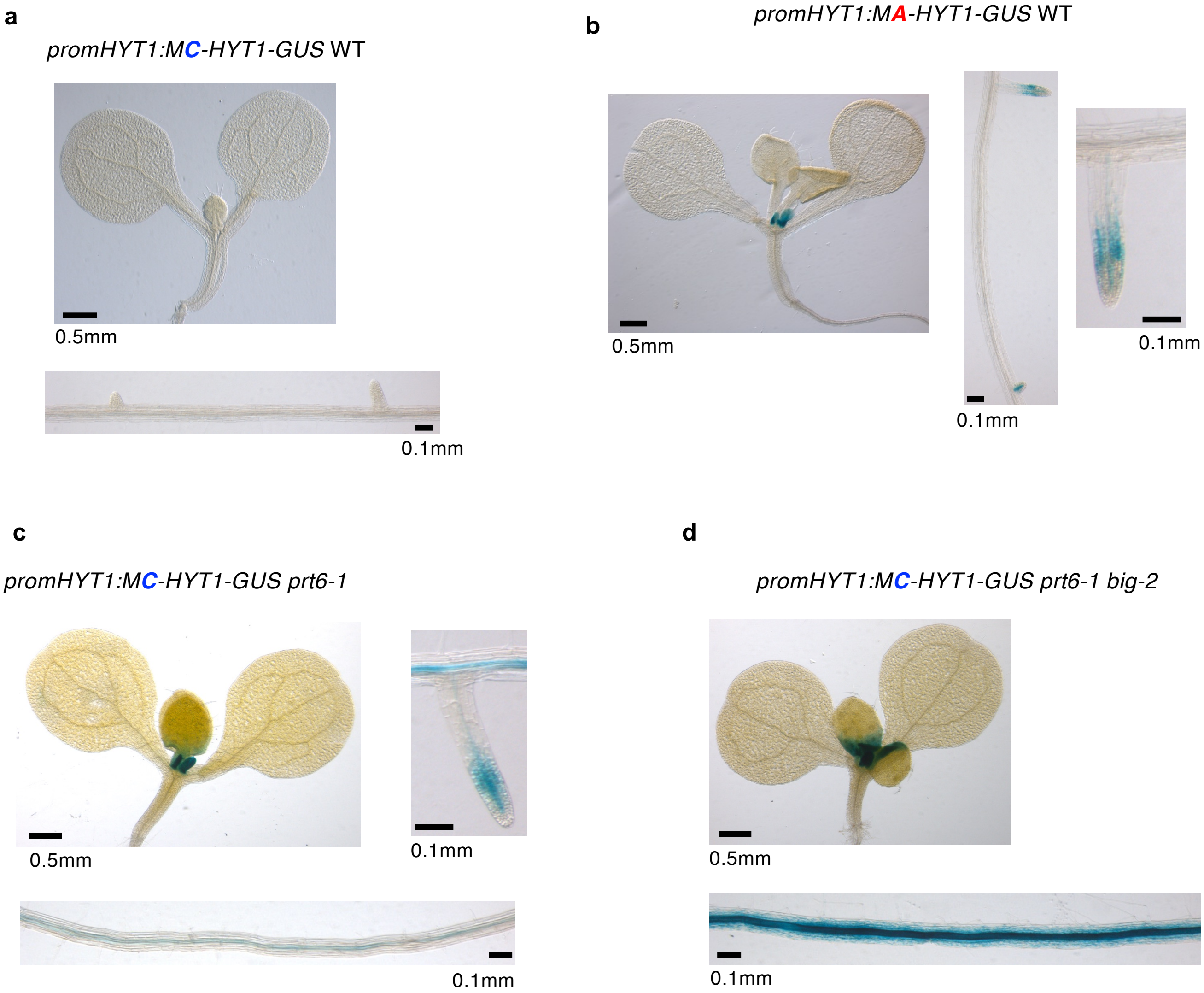

Supplementary Fig. 1 l HYT1-GUS accumulation pattern in seedlings
Accumulation of Cys2 or Cys2Ala-HYT1-GUS driven by the HYT1 promoter in Col-0 (a, b), *prt6-1* (c), or *prt6-1 big-2* (d) in 7 day old seedlings. The experiment was repeated three times with similar results.

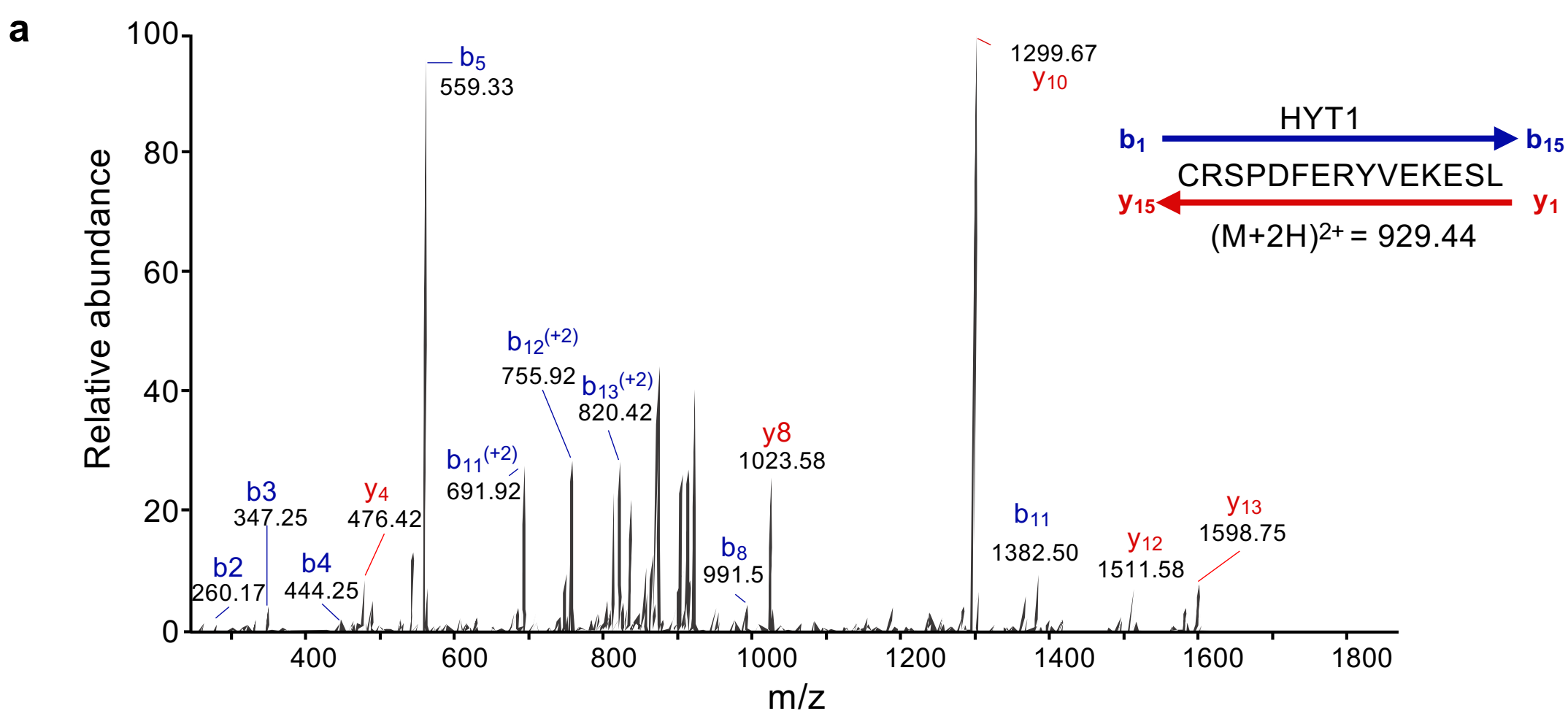

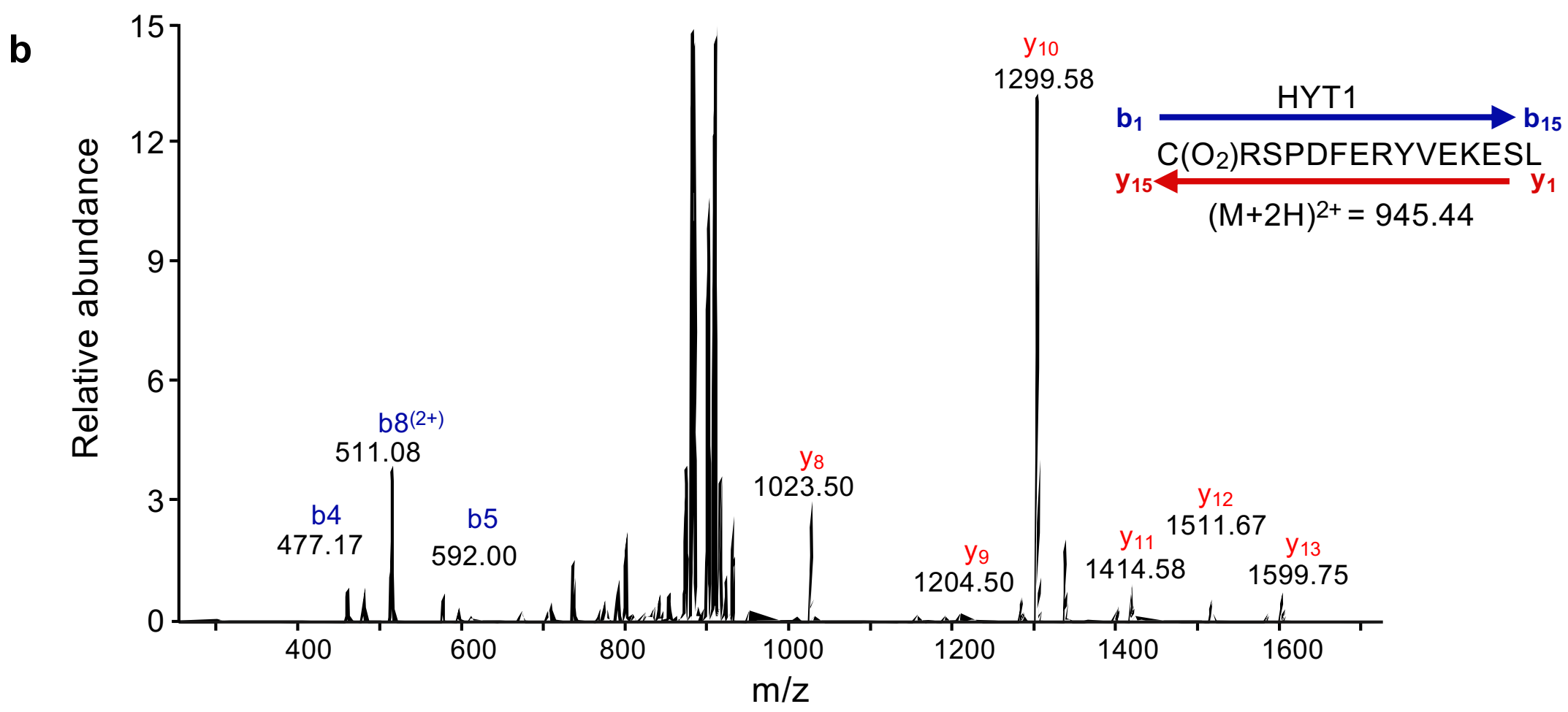

Supplementary Fig. 2. Analysis of the chemical structure at the amino-termini of HYT1

a. MS/MS spectra of the HYT(2-16) peptide in the absence of PCO4 enzyme, showing the reduced Cysteine ($[M+2H]^{2+}$ = 924.44)

b. MS/MS spectra of the HYT(2-16) peptide after incubation of PCO4 enzyme, showing the oxidised Cysteine (Cys-sulfinate) ($[M+2H]^{2+}$ = 945.44)

c

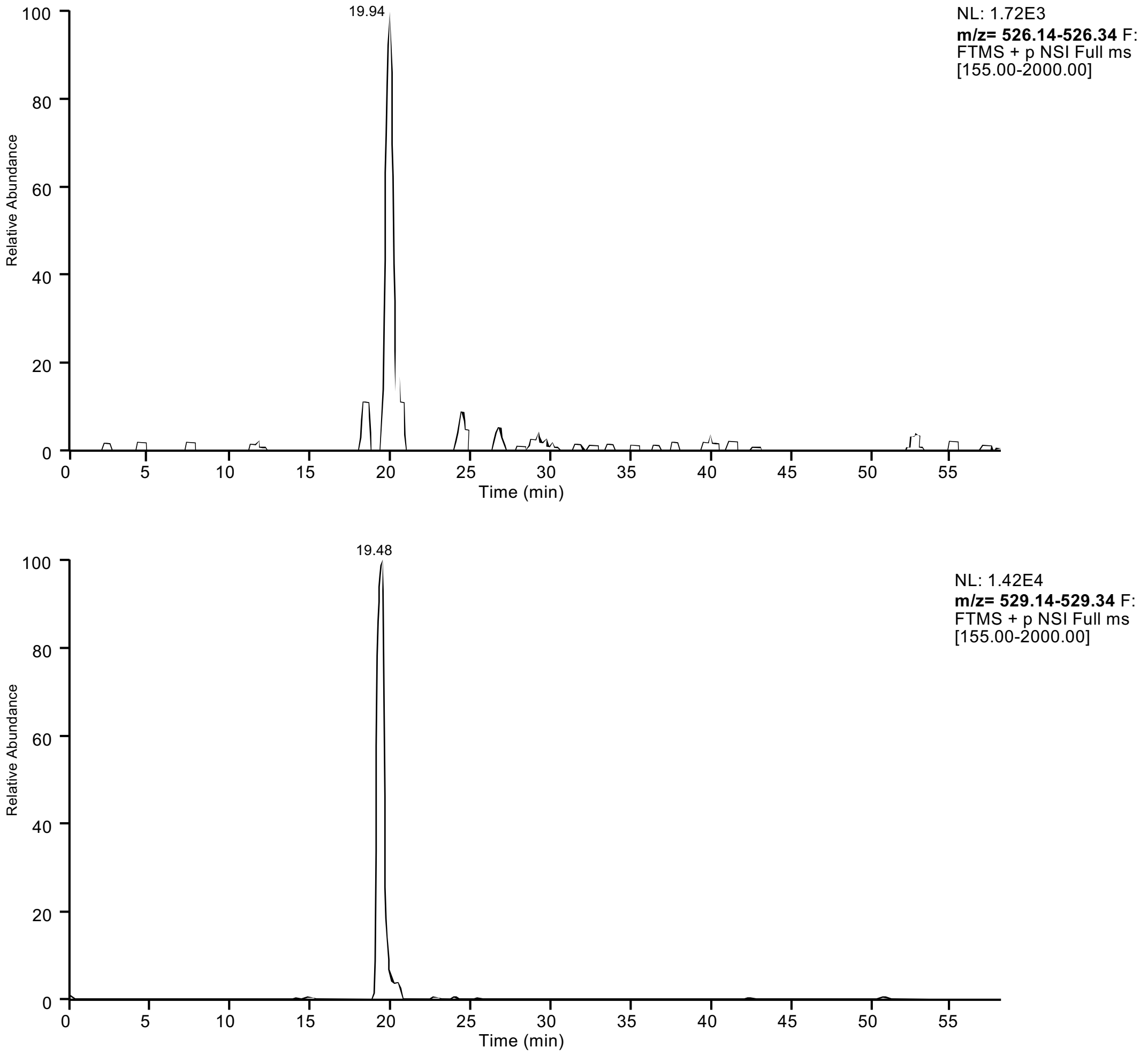

d

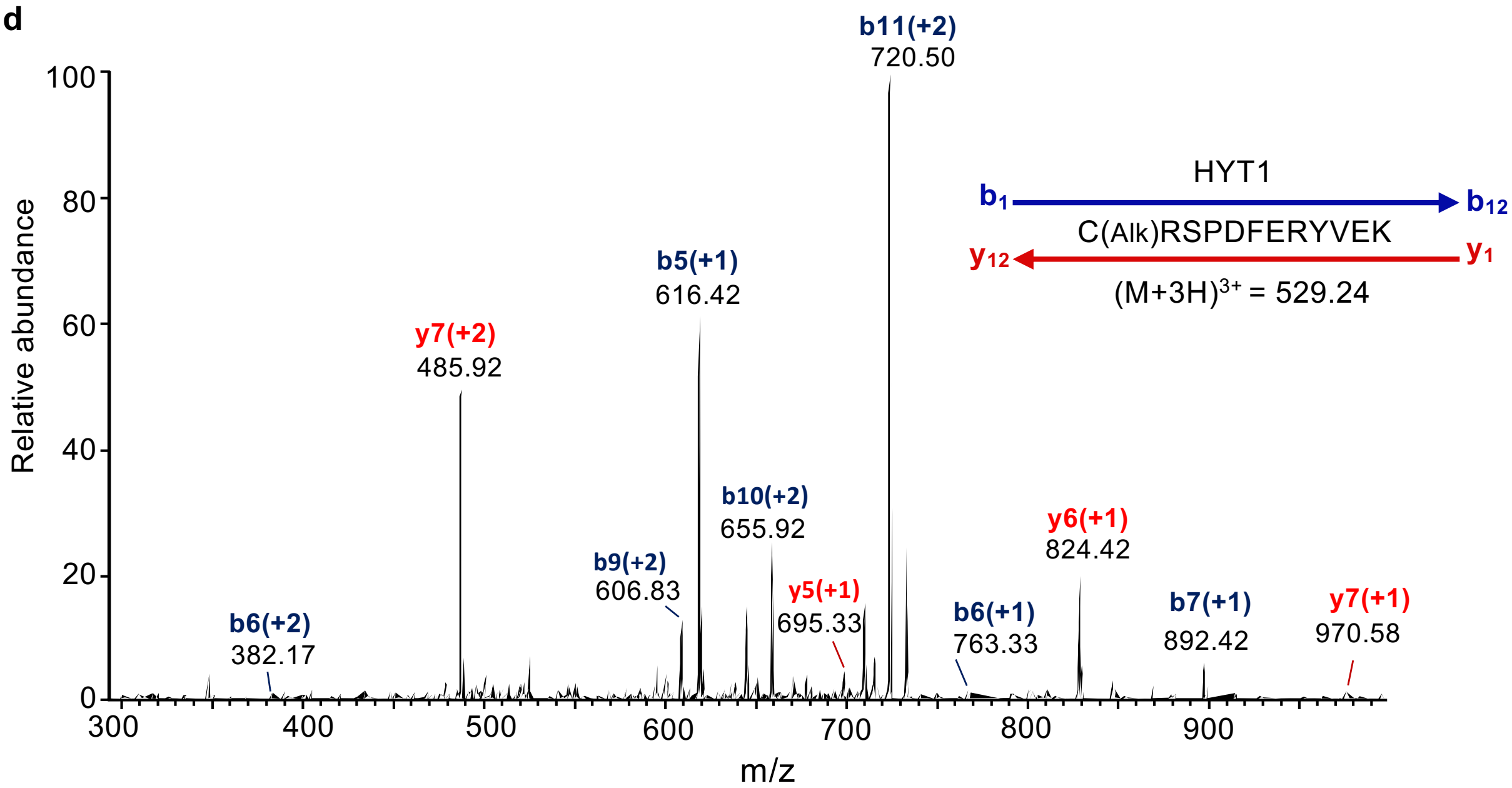

Supplementary Fig. 2. Analysis of the chemical structure at the amino-termini of HYT1 (continued)
c. Extracted ion chromatograms (EIC) of Cys sulfonate-HYT1 $[M+3H]^{3+}$ = 526.24 (top) and HYT1 with Alkylated Cys $[M+3H]^{3+}$ = 529.24 (bottom). EIC was obtained from full MS scans.
d. MS/MS spectra showing identified b and y-series ions of HYT1 Nt peptide after digestion with LysC ($[M+3H]^{3+}$ = 529.24) following expression in a wheat germ extract, showing the presence of alkylated cysteine at the amino-terminus.

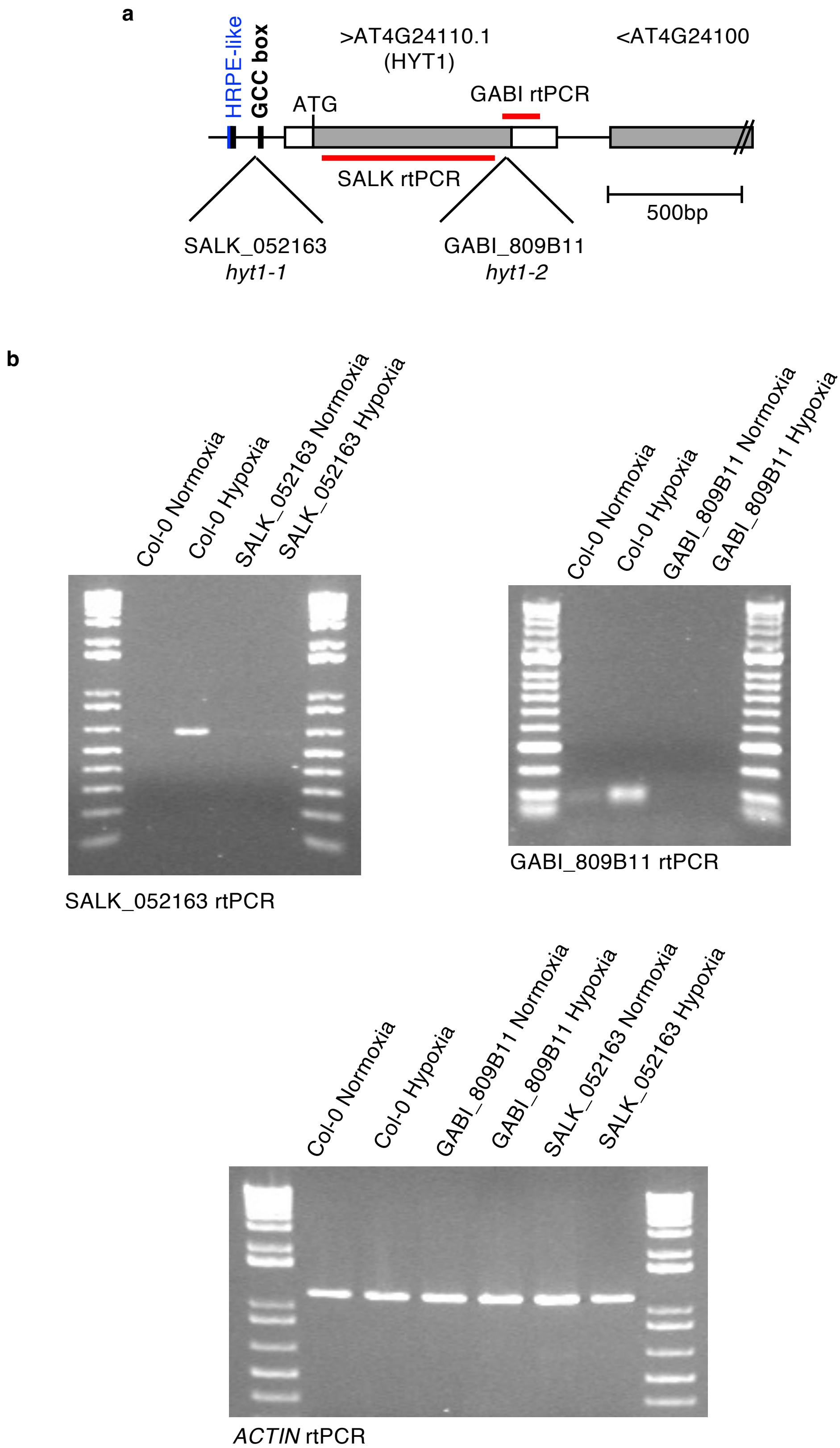

Supplementary Fig. 3 l Characterisation of *HYT1* mutant alleles.

a. Schematic representation of *HYT1* gene region showing relative positions of insertion lines SALK_052163 and GABI_809B11. Blue/black bars are the positions of HRPE and GCC elements respectively, white boxes 5' and 3' UTRs, grey box protein coding region. Direction of genes are indicated by > or <.

b. RT-PCR analysis of *HYT1* expression in SALK_052163 (*hyt1-1*) and GABI_809B11 (*hyt1-2*) TDNA insertion mutants. The region amplified is indicated in a. Actin was used as a positive control for RNA in all samples (bottom panel).

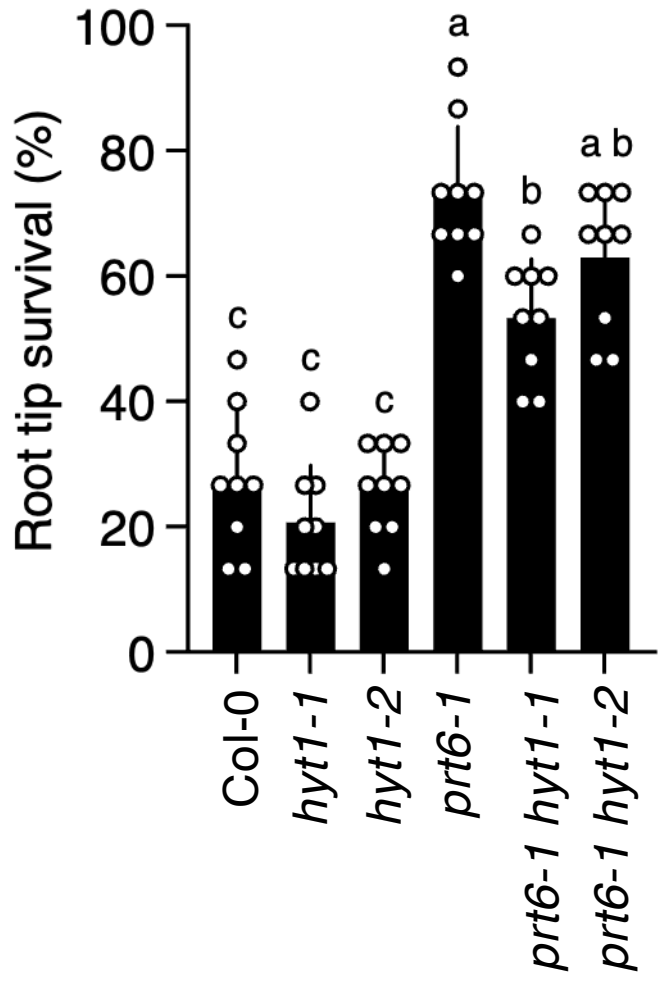

Supplementary Fig. 4 | Phenotypic influence of *hyt1* mutant alleles.
Root tip survival response to transient extreme ambient hypoxia (4h, < 0.5% O_2), measured after 3 days recovery in normoxia.
Data are presented as mean ± SD (n = 9) significant differences denoted with letters for one-way ANOVA ($p<0.05$).

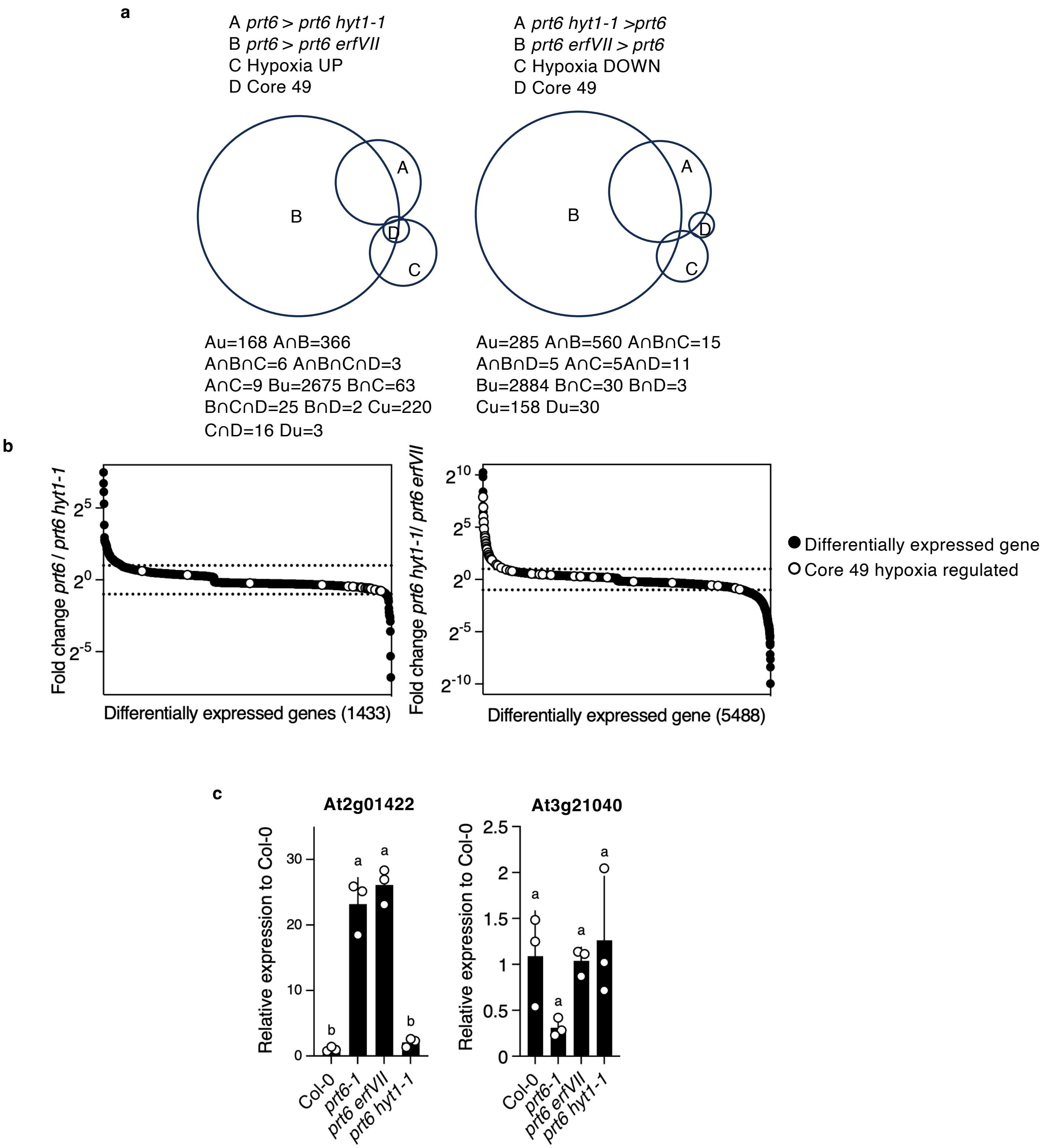

Supplementary Fig. 5 I Expression of transcripts in WT and mutant seedlings.

a. Proportional Venn diagram analysis of overlapping genes comparing regulation by *ERFVII*s, *HYT1* and hypoxia. All transcripts up-regulated in the described comparisons were included in the analysis. Hypoxia up-regulated transcripts were obtained from Gibbs *et al* (2014) Molecular Cell; 53 369-379. Core 49 hypoxia up-regulated genes were obtained from Mustroph et al. (2009) PNAS (USA); 106 18843-18848.

b. Differentially expressed genes in transcriptome comparisons *prt6-1* vs. *prt6-1 hyt1-1* and *prt6-1 hyt1-1* vs. *prt6-1 erfVII*, sorted by fold difference. Positions of the core 49 hypoxia genes are shown.

c. Expression of genes specifically regulated by *HYT1*. Identified as either up-regulated in *prt6-1* compared to *prt6 hyt1-1* (At2g01422) or up-regulated in *prt6 hyt1-1* compared to *prt6-1* (At3g21040). Data are presented as mean ± SD (n = 3) significant differences denoted with letters for one-way ANOVA ($p<0.05$).

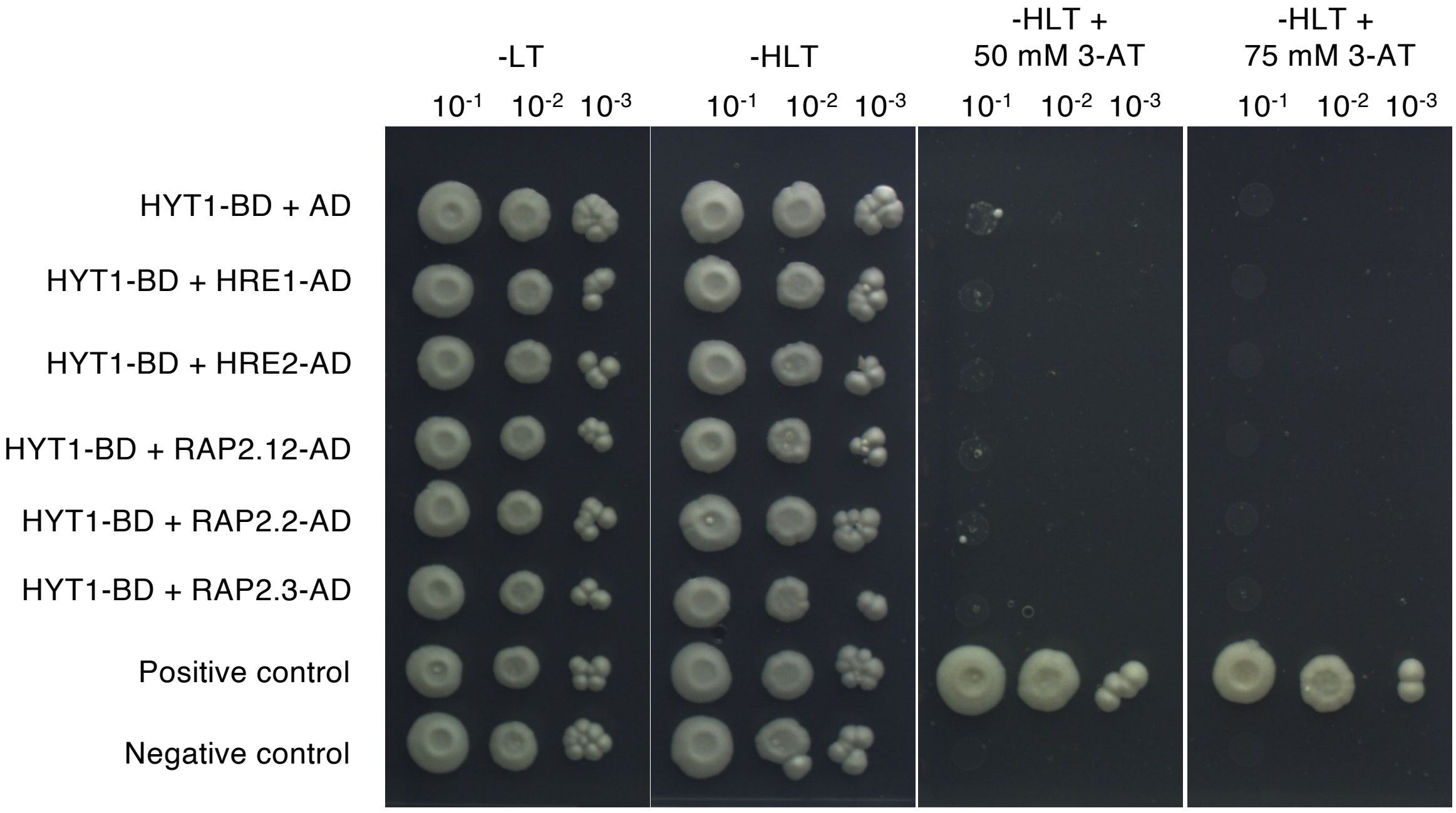

Supplementary Fig. 6 l Yeast two hybrid analysis of HYT1 interaction with ERFVIIs. HYT1 was used as the bait and ERFVIIs as the prey. Yeast cells co-transformed with bait and prey constructs were grown on LW (SD/ -Leu – Trp) and HLW (SD/ -Leu – Trp -His) media supplemented with 3-AT. Co-transformed pEXP32/Krev1 + pEXP22/RALGDS-wt and pEXP32/Krev1 + pEXP22/RAL/GDS-m1 were used as positive and negative controls respectively.

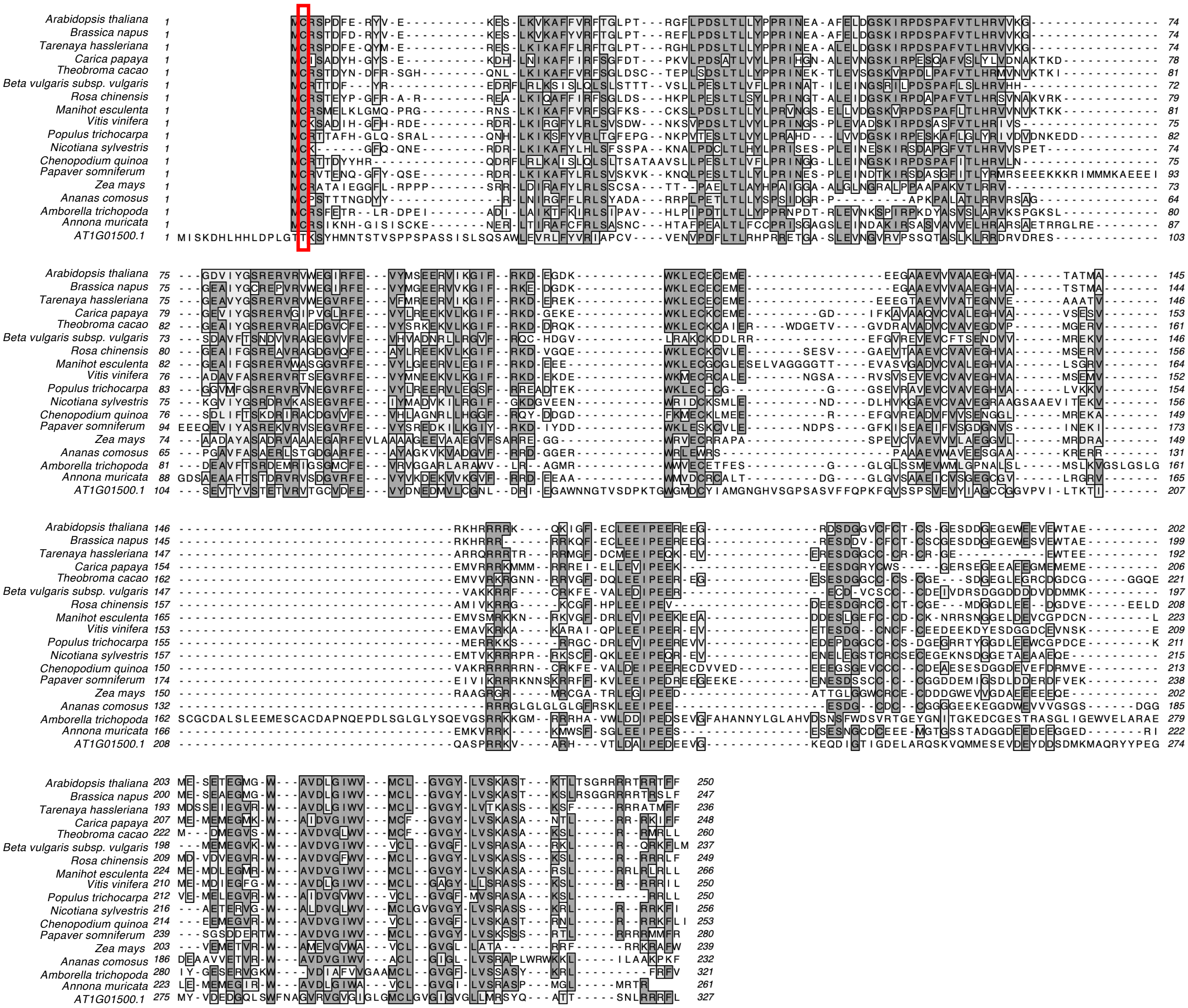

Supplementary Fig. 7 | HYT1 related sequences are found in diverse angiosperm species, and all contain Cys-2 | MUSCLE alignment of HYT1 with similar sequences identified through BLASTp search at NCBI or 1kp *(Annona muricata)*. The closest low homology sequence from *Arabidopsis thaliana* is included (AT1G01500.1). Identical residues in dark grey, similar residues in light grey. Sequences are presented in Supplementary Data 4. Cys-2 is highlighted in red.

a

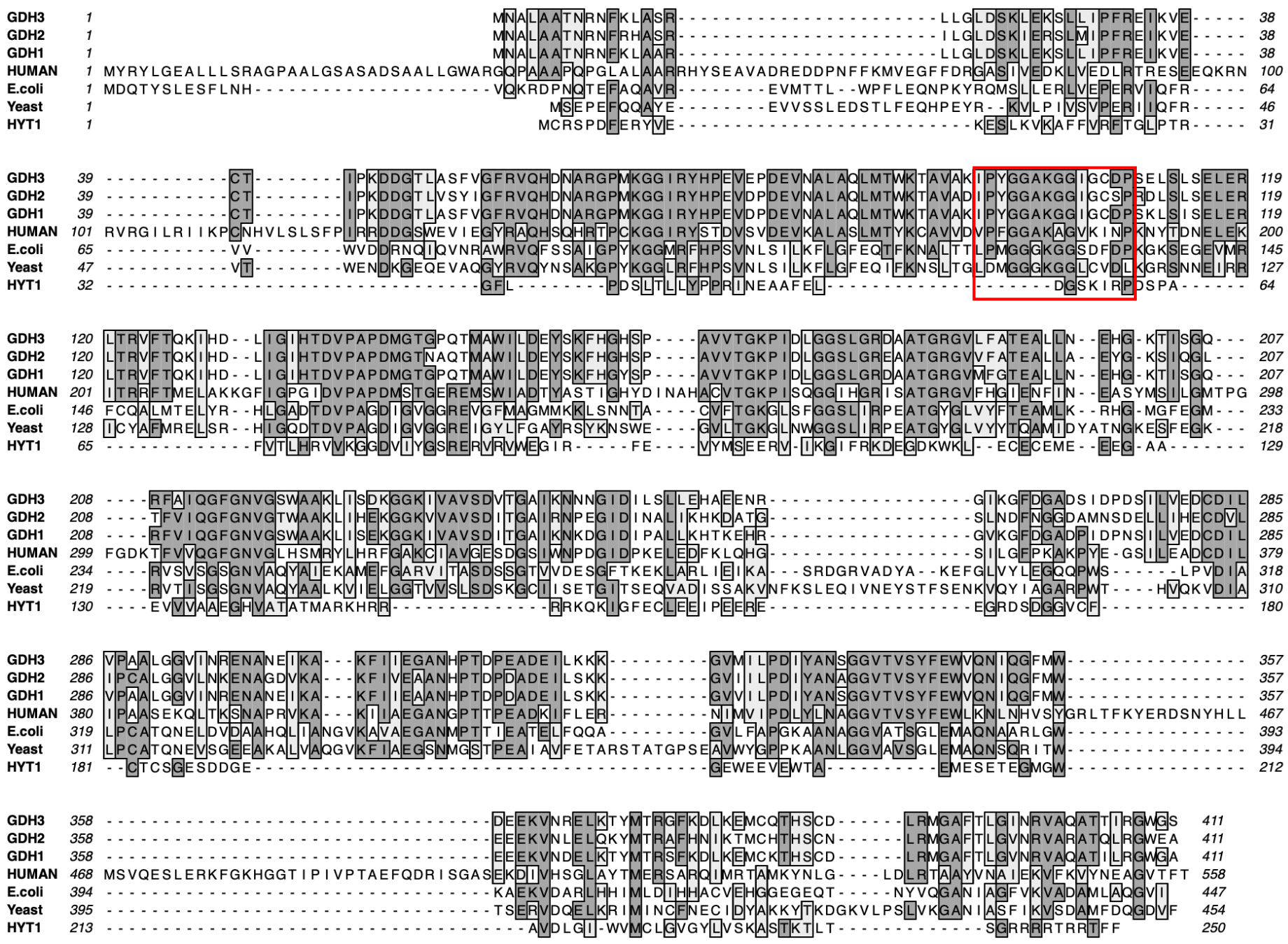

b

GLFV_DEHYDROGENASE, PROSITE PS00074; Glu / Leu / Phe / Val dehydrogenases active site consensus:

[LIV]-x(2)-G-G-[SAG]-K-x-[GV]-x(3)-[DNST]-[PL]

c

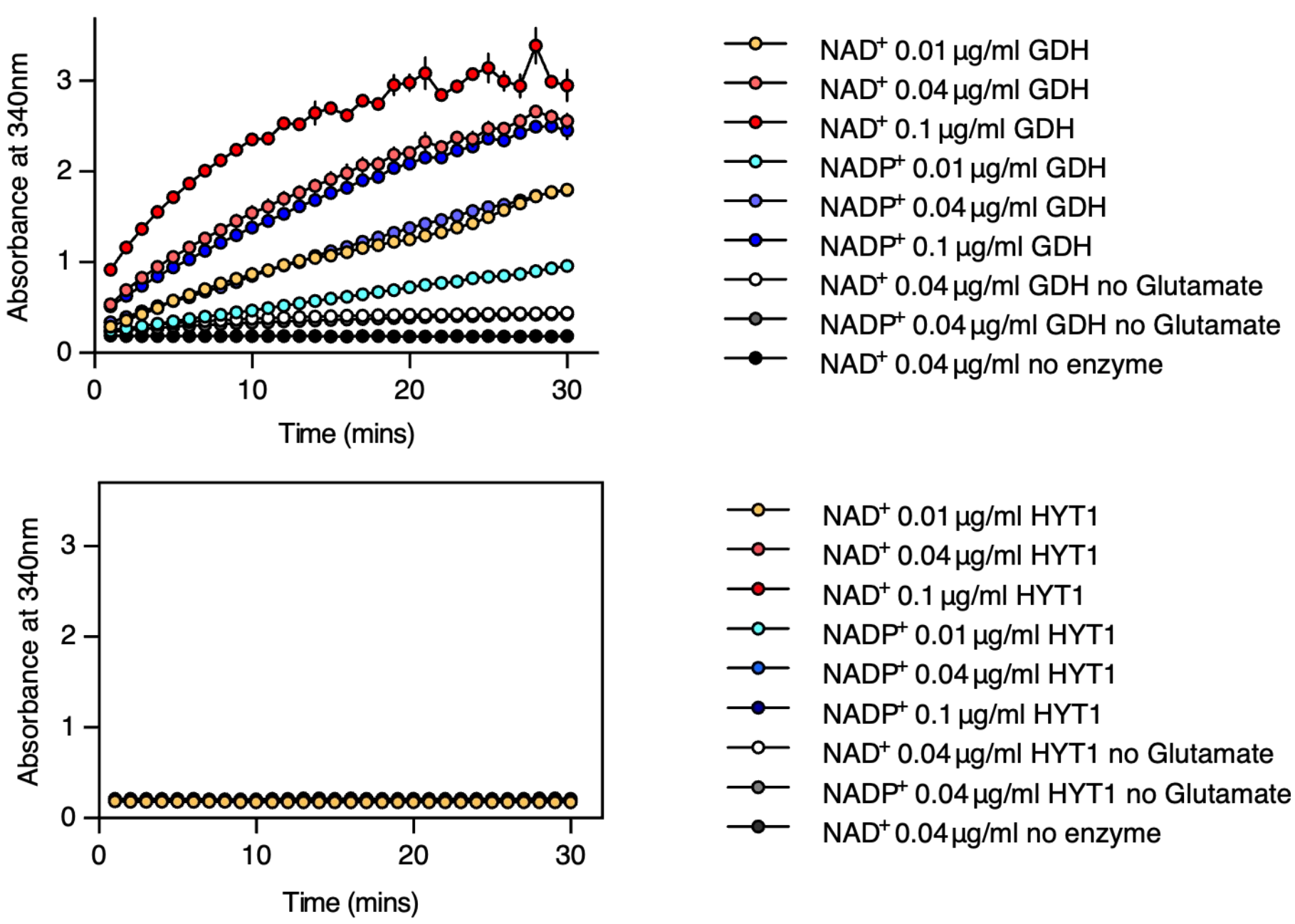

d

Aldehydes
Acetaldehyde
Glyoxylic acid monohydrate
Salicylaldehyde
Paraformaldehyde
Glutaraldehyde
α-Keto Acids
Sodium Pyruvate
α-Ketoglutaric acid
α-Keto Acids
Sodium Pyruvate
Alcohols
Ethanol
Butanol
Glycerol
Sugars & Sugar Acids
Glucose
L-(+)-Arabinose
Galacturonic acid
Ribose
Erythrose 4-phosphate
Amino Acids
L-Glutamic acid
L-Alanine
L-Proline
L-Isoleucine
L-Phenylalanine
Organic Acids
Malic acid
Sodium acetate
Formic acid
Disodium succinate
Lactic acid

Supplementary Fig. 8 I HYT1 is not an NADP-SPECIFIC GLUTAMATE DEHYDROGENASE.
a. MUSCLE alignment of HYT1 with NADP-SPECIFIC GLUTAMATE DEHYDROGENASEs from *A. thaliana* (GDH1-3), Human, *E. coli* and yeast (*Saccharomyces cerevisiae*). Identical residues in dark grey, similar residues in light grey. Active site region boxed in red.
b. Active site consensus for GLFV_DEHYDROGENASE, PROSITE PS00074
c. Assay for glutamate dehydrogenase activity for HYT1 via reduction of NAD/P (increased absorbance at 340nm), using Glutamate as a substrate. Glutamate Dehydrogenase (GDH) was used as a positive control, 0.1 µg = 0.0044units; Sigma G2626. Data presented as mean ± SD (n = 3).
d. List of simple organic chemicals assessed through NAD/P dehydrogenase assay for HYT1 activity. All tested potential substrates were negative.

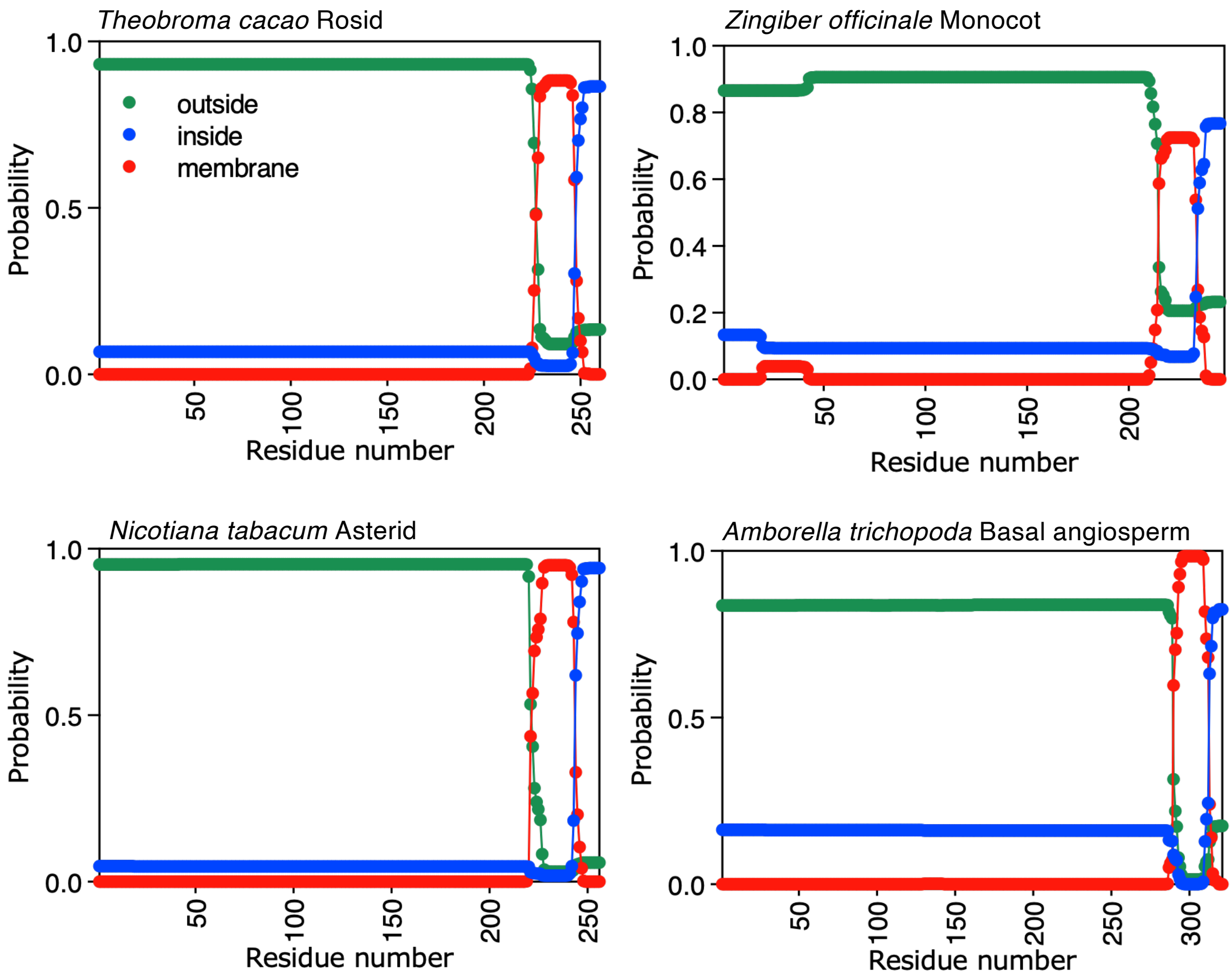

Supplementary Figure 9 I HYT1 like sequences contain a predicted Tail Anchored domain.
Exemplar angiosperm HYT1-like sequences are shown. Transmembrane helices were predicted and visualised using TMHMM - 2.0. The plot shows posterior possibilities of residue location inside (blue), outside (green) or within (red) a membrane. Sequences are in Supplementary Data 4.

A. Krogh, B. Larsson, G. von Heijne, and E. L. L. Sonnhammer. Predicting transmembrane protein topology with a hidden Markov model: Application to complete genomes. Journal of Molecular Biology, 305(3):567-580 (2001).

a

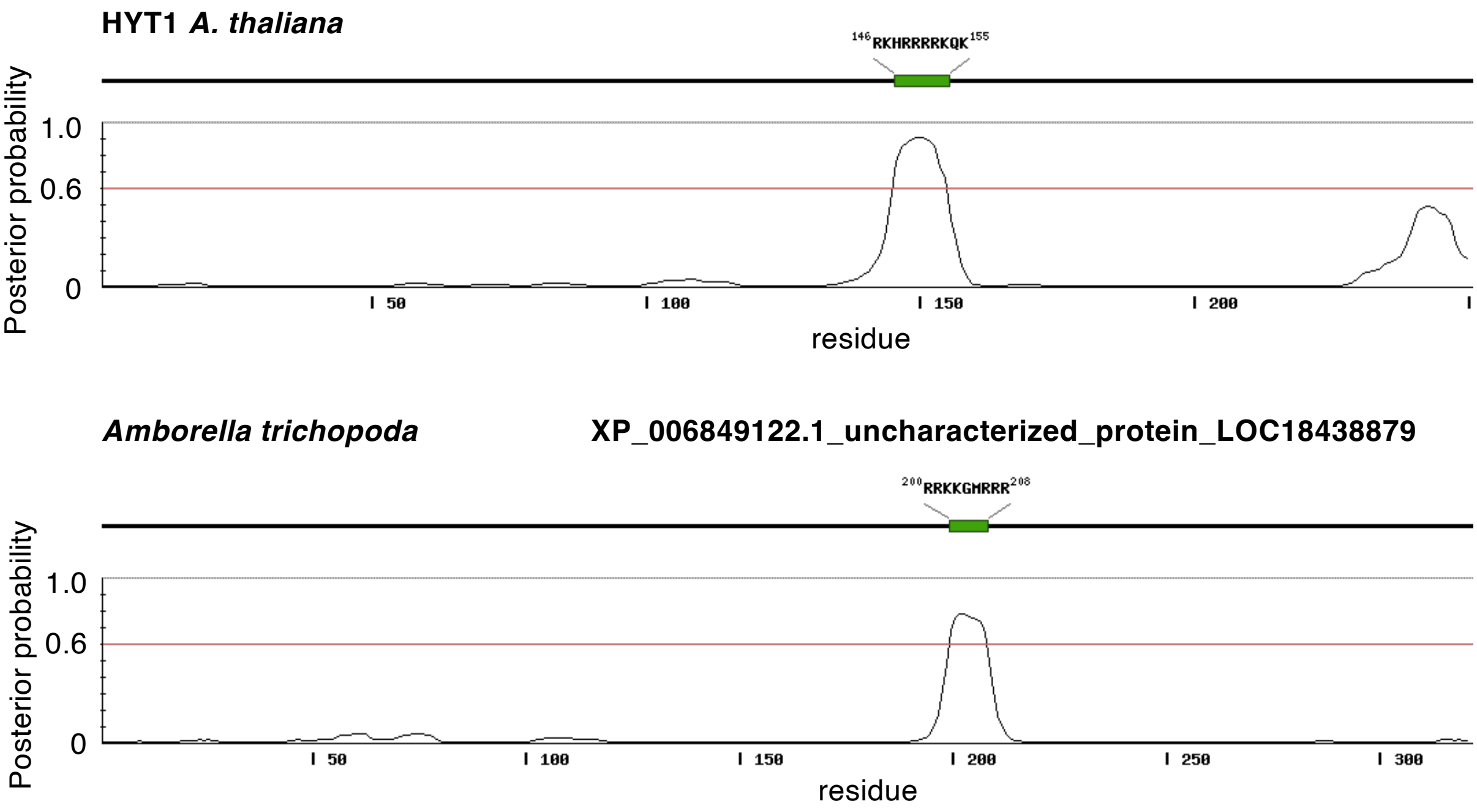

b

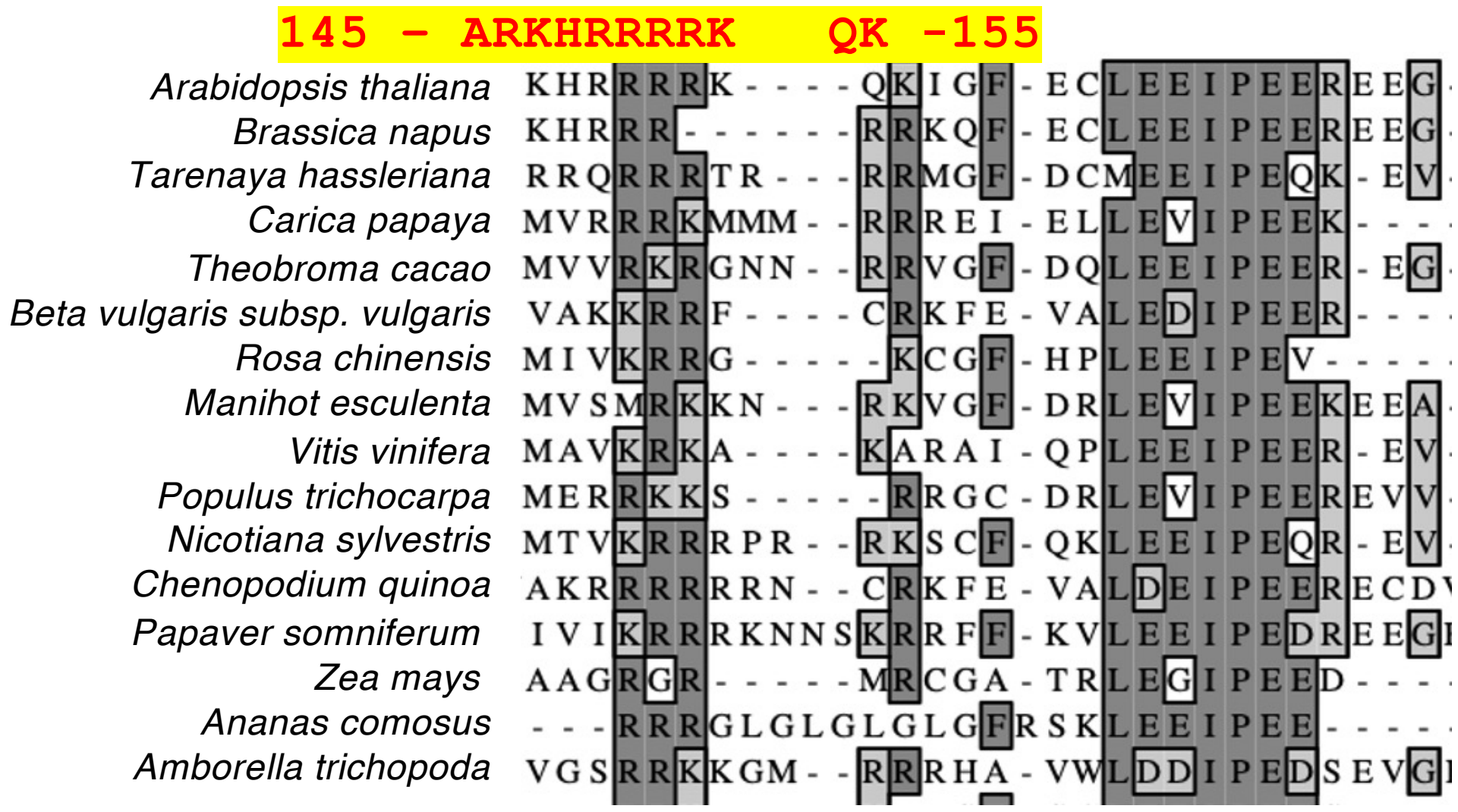

Supplementary Fig. 10 I Conserved nuclear localisation prediction for HYT1. Both HYT1, and HYT1-like protein from *A. trichopoda* have predicted nuclear localisation signals at similar positions.

a. The nuclear localisation signal predictor programme NLStradamus was used to determine the presence of a nuclear localisation signal in HYT1 protein sequences from *A. thaliana* and *A. trichopoda*. Pre-loaded model was 2 state HMM static (based on data modelled in the programme) with a posterior threshold prediction cut off of 0.6 (0,1).

This analysis identifies a nuclear-location signal sequence at positions 145-155 of HYT1. http://www.moseslab.csb.utoronto.ca/NLStradamus/

b. Sequence comparison of angiosperm HYT1-like sequences around the predicted location of a nuclear localisation signal.

a. RFP-HDEL GFP-HYT1 Merged c.

b.

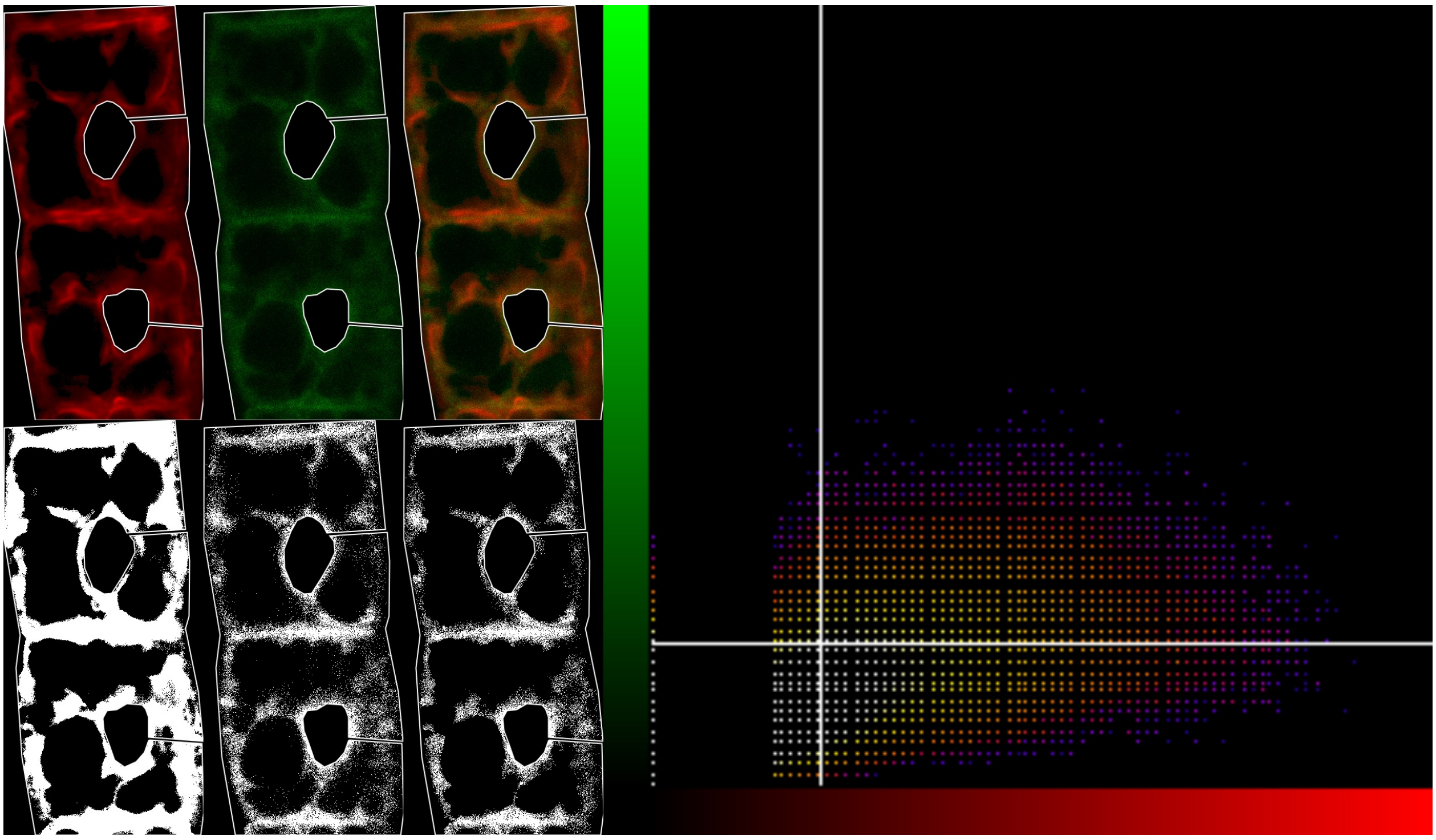

d.

Cell number analysed.	Manual Threshold A	Manual Threshold B	Thresholded Manders Coefficient 1 M1	Thresholded Manders Coefficient 2 M2	ROI Area (total)	Area in red channel with above threshold pixels in ROI	Area in green channel with above threshold pixels in ROI	Area Overlap channels	% green in area overlap
1	44	39	0.633	0.965	8.896	4.11	2.318	2.227	96.0742
2	44	39	0.747	0.963	7.122	3.489	2.446	2.338	95.58463
3	44	39	0.565	0.971	6.171	3.76	1.928	1.865	96.73237
4	44	39	0.609	0.932	9.128	3.787	2.18	2.015	92.43119
5	53	44	0.444	0.868	37.763	15.583	7.231	6.192	85.63131
6	53	47	0.382	0.92	20.844	8.3	3.047	2.781	91.2701
7	53	47	0.416	0.893	19.699	9.042	3.783	3.336	88.18398
		**Average Thresholded**	**0.5422**	**0.9302**				**Average**	**92.27254**
		**SD**	**0.1331**	**0.0393**				**SD**	**4.226206**

Supplementary Fig. 11 I Analysis of the co-occurrence of GFP-HYT1, RFP-HDEL and SUN1-RFP. Data output screenshots from JACoP BIOP, FIJI (ImageJ]

a. Example images of the ROI (region of interest) in epidermal cells from the rapid elongating zone in roots where co-occurrence was measured between green (GFP-HYT1) and red (RFP-HDEL) channels and the merged channel. Nuclei are excluded from ROI because these do not contain RFP-HDEL.

b. Output images from ROI with thresholded mask for each channel and colocalized pixels in white in the merged channel.

c. Fluorogram (scatter plot) of ROI, showing the correlation of pixel intensity over all pixels in the image. In the right quadrant appear pixels that correlate in the two channels.

d. Measurement of co-occurrence for seven cells:

Output from JACoP BIOP, FIJI. Including the thresholds manually selected for each cell measured and the corresponding output Thresholded Mander's coefficients M1 and M2 for all cells measured. The table also includes the area occupied by thresholded pixels in each channel and the area overlap (colocalized).

Co-occurance between GFP-HYT1 and RFP-HDEL was quantified using Manders' thresholded coefficients M1 and M2, calculated using BIOP's version of JACoP in FIJI.

A value of M2 0.9302+/-0.0393 indicates that 93% of the GFP co-occurs with the RFP suggesting that it is ER localised.

Fig. continued on second sheet

e. SUN1-RFP GFP-HYT1 Merged

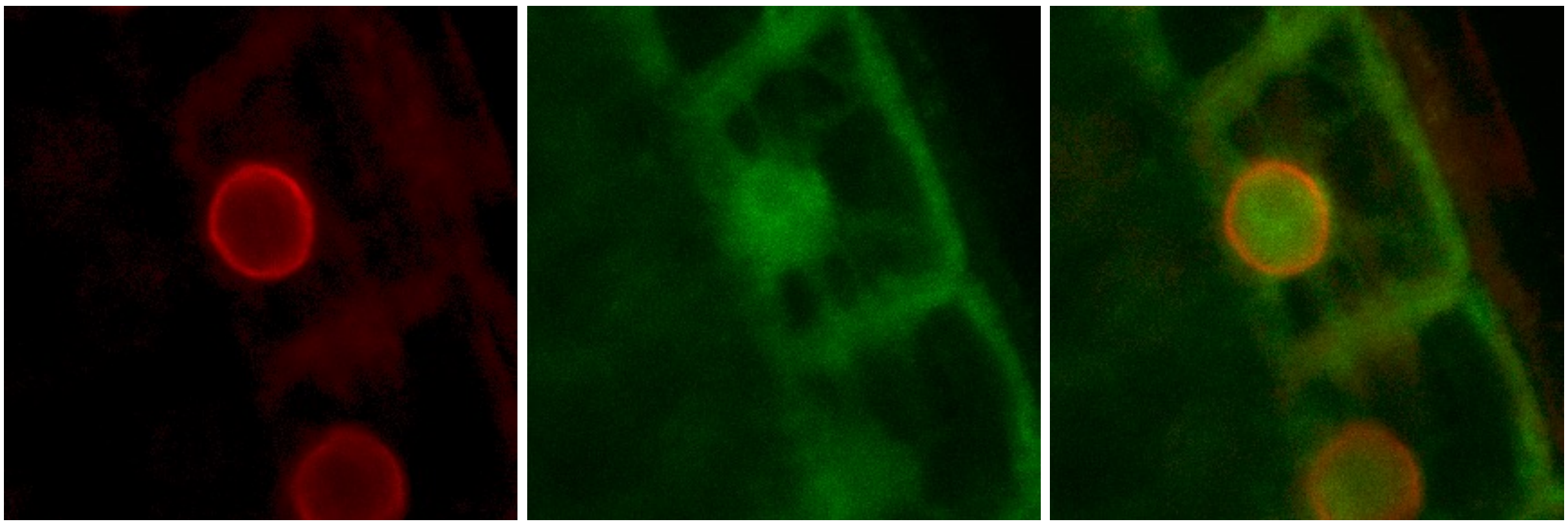

f. SUN1-RFP GFP-HYT1 Merged

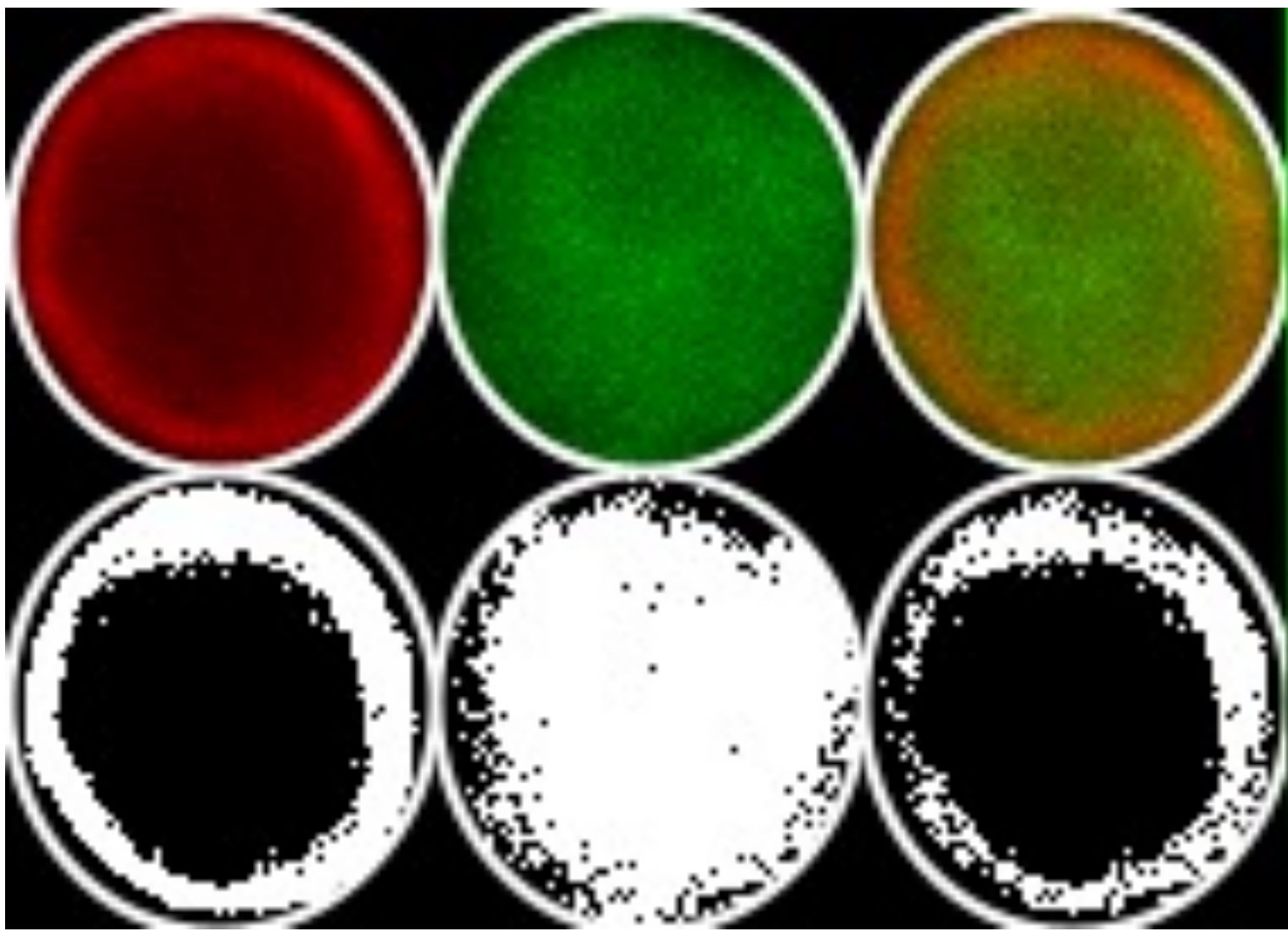

g.

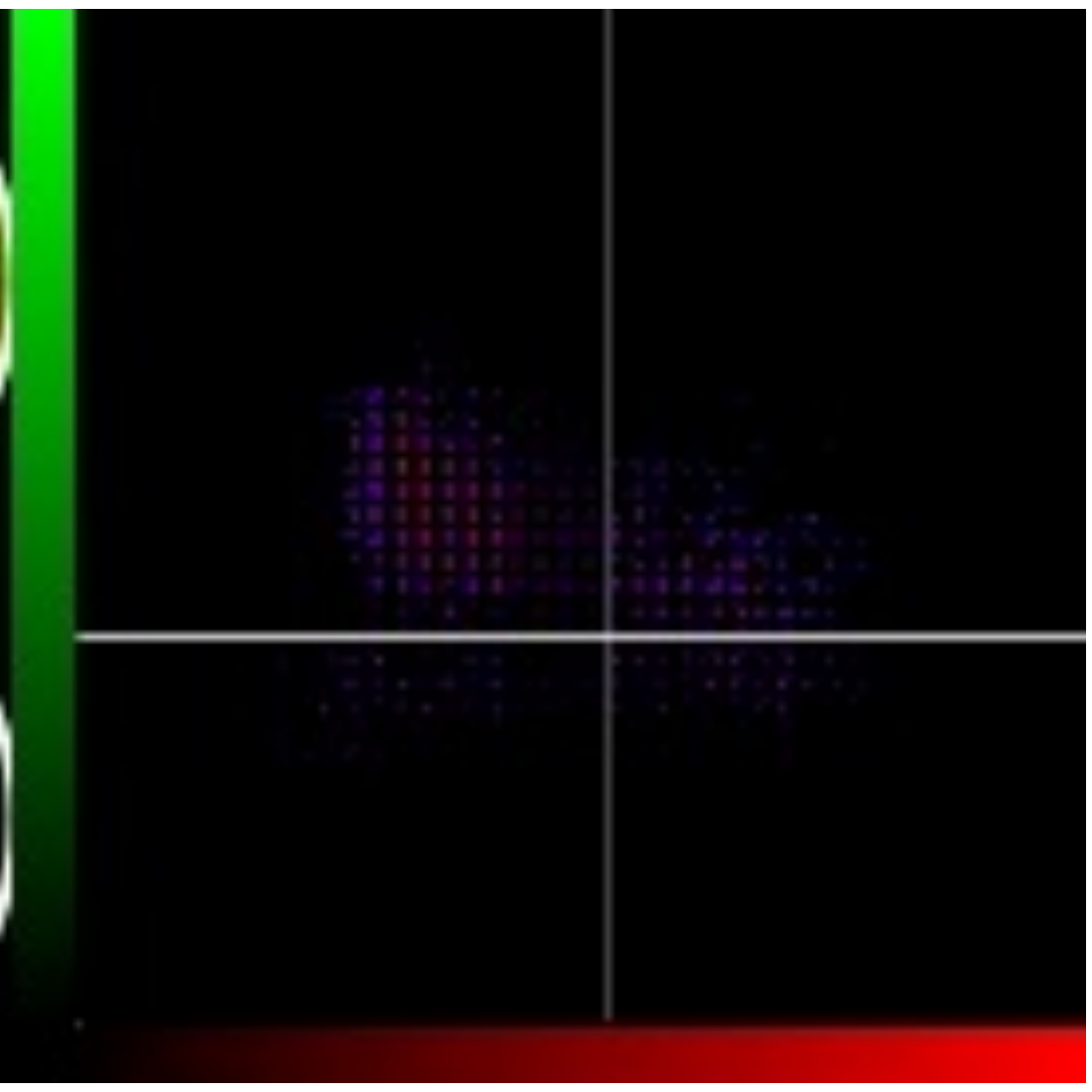

h.

Cell number analysed.	Manual Threshold A	Manual Threshold B	Thresholded Manders Coefficient 1 M1	Thresholded Manders Coefficient 2 M2	ROI Area (total)	Area in red channel with above threshold pixels in ROI	Area in green channel with above threshold pixels in ROI	Area Overlap channels	% green in area overlap	% red in area overlap
1	102	47	0.627	0.31	0.065	0.02	0.042	0.013	30.95238	65
2	102	47	0.845	0.317	0.065	0.021	0.055	0.018	32.72727	85.71429
3	102	47	0.964	0.271	0.068	0.018	0.06	0.017	28.33333	94.44444
4	102	47	0.826	0.302	0.071	0.02	0.057	0.017	29.82456	85
5	67	39	0.855	0.316	0.331	0.105	0.263	0.089	33.8403	84.7619
6	67	39	0.999	0.543	0.263	0.132	0.243	0.132	54.32099	100
7	67	39	0.855	0.312	0.358	0.105	0.266	0.089	33.45865	84.7619
8	67	39	0.996	0.598	0.228	0.123	0.211	0.122	34.77964	99.18699
9	67	39	0.977	0.784	0.225	0.174	0.217	0.169	35.32639	97.12644
10	67	39	0.808	0.806	0.247	0.176	0.164	0.135	35.6977	76.70455
		Average Thresholded	0.8752	0.4559				Average	34.92612	87.27
		SD	0.1147	0.2099				SD	7.229466	10.96

Supplementary Fig. 11 (cont) I Analysis of the co-occurrence of GFP-HYT1, HDEL-RFP and SUN1-RFP.

Data output screenshots from JACoP BIOP, FIJI (ImageJ]

e. Images of the ROIs (region of interest) where co-occurrence was measured between green (GFP-HYT1) and red (SUN1-RFP) channels and the merged channel. Only nuclei were analysed as the ROI because of the localisation of SUN1 to the inner nuclear membrane.

f. Output image with thresholded mask for each channel and colocalized pixels in white in the merged channel.

g. Fluorogram (scatter plot), showing the correlation of pixel intensity over all pixels in the image. In the right quadrant appear pixels that correlate in the two channels.

h. Measurement of co-occurrence for seven cells:

A value of M1 0.8752+/-0.1147 indicates that 88% of the RFP co-occurs with the GFP suggesting that a proportion of the GFP signal is localised to the inner nuclear membrane.

The experiment was repeated three times with similar results.

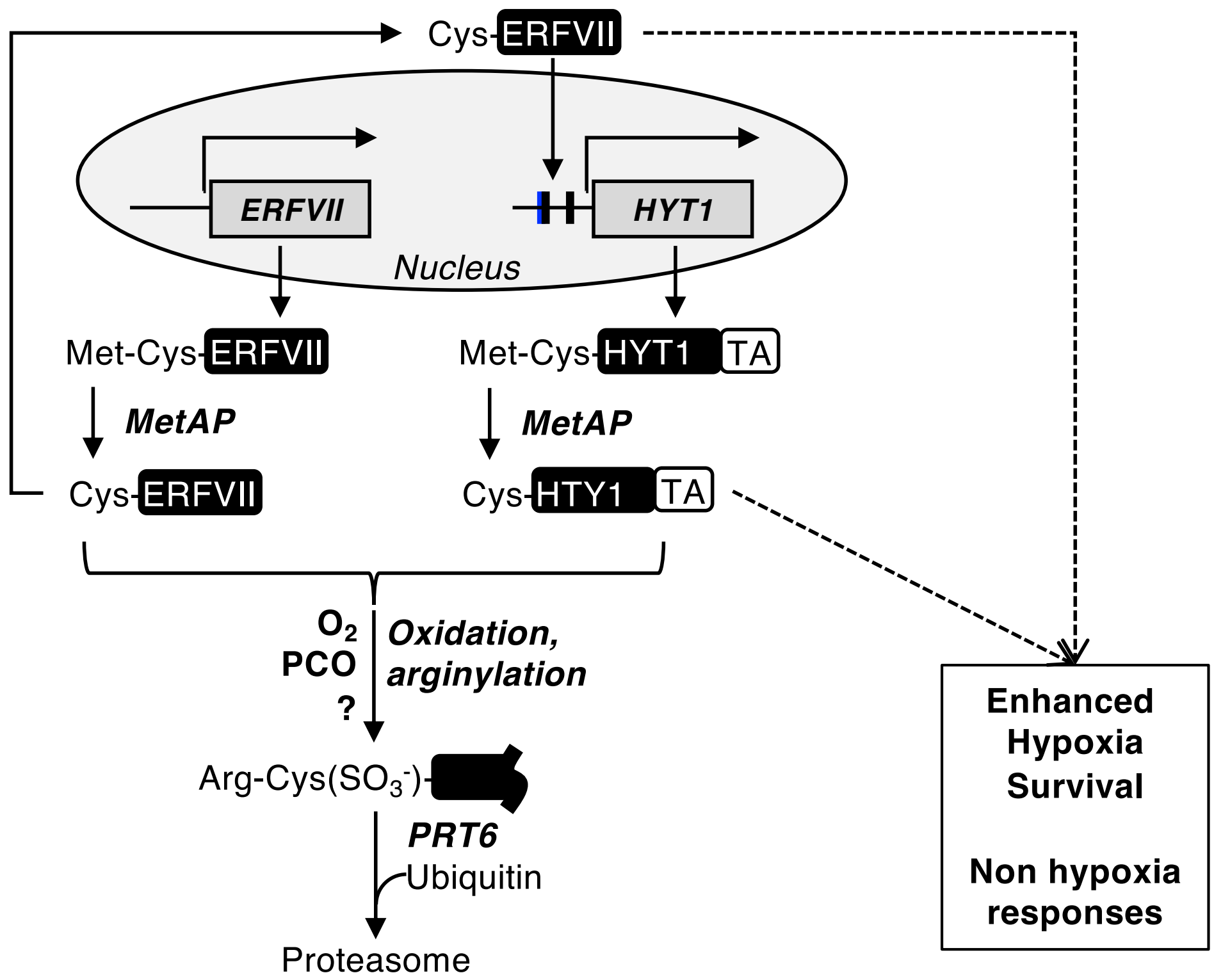

Supplementary Fig. 12 | Expanded model for ERFVII and HYT1 regulation and functions in oxygen sensing.

Genes are indicated as square boxes, proteins as rounded boxes. Solid lines with arrows indicate direct effect, broken lines indirect effect. Cis elements are indicated upstream of the *HYT1* gene. Three letter amino-acid codes are used. Refer to Fig. 1 for descriptions of enzymes.
