# Supplementary material for: Hierarchical regulation of oxygen-sensing through coupled transducers in Arabidopsis thaliana
Source: Nat Commun. 2026 Sep 16;17:10861. doi: 10.1038/s41467-026-77710-x (PMC13672779; doi:10.1038/s41467-026-77710-x)
Supplement: Supplementary file 2 [file 41467_2026_77710_MOESM2_ESM.pdf]

Description of Additional Supplementary Files

File Name: Supplementary Data 1

Description: List of Arabidopsis proteins initiating Met-Cys.

File Name: Supplementary Data 2

Description: Comparison of all genes differentially regulated in *prt6* vs *prt6 hyt1-1*.

File Name: Supplementary Data 3

Description: Comparison of all genes differentially regulated in *prt6 hyt1-1* vs *prt6* with those differentially regulated in *prt6 erfVII* vs *prt6* (Zubrycka et al Nat Comms 2023).

File Name: Supplementary Data 4

Description: List of angiosperm proteins with similarity to HYT1.

File Name: Supplementary Data 5

Description: List of oligonucleotide primers used for genotyping or QrtPCR.
