# Supplementary material for: Hierarchical regulation of oxygen-sensing through coupled transducers in Arabidopsis thaliana
Source: Nat Commun. 2026 Sep 16;17:10861. doi: 10.1038/s41467-026-77710-x (PMC13672779; doi:10.1038/s41467-026-77710-x)
Supplement: Supplementary file 3 [file 41467_2026_77710_MOESM8_ESM.pdf]

nature portfolio

Corresponding author(s): Holdsworth

Last updated by author(s): Aug 14, 2026

Reporting Summary

Nature Portfolio wishes to improve the reproducibility of the work that we publish. This form provides structure for consistency and transparency in reporting. For further information on Nature Portfolio policies, see our Editorial Policies and the Editorial Policy Checklist.

Statistics

For all statistical analyses, confirm that the following items are present in the figure legend, table legend, main text, or Methods section.

n/a	Confirmed	
[ ]	[x]	The exact sample size (*n*) for each experimental group/condition, given as a discrete number and unit of measurement
[ ]	[x]	A statement on whether measurements were taken from distinct samples or whether the same sample was measured repeatedly
[ ]	[x]	The statistical test(s) used AND whether they are one- or two-sided<br>*Only common tests should be described solely by name; describe more complex techniques in the Methods section.*
[x]	[ ]	A description of all covariates tested
[ ]	[x]	A description of any assumptions or corrections, such as tests of normality and adjustment for multiple comparisons
[ ]	[x]	A full description of the statistical parameters including central tendency (e.g. means) or other basic estimates (e.g. regression coefficient) AND variation (e.g. standard deviation) or associated estimates of uncertainty (e.g. confidence intervals)
[ ]	[x]	For null hypothesis testing, the test statistic (e.g. F, t, r) with confidence intervals, effect sizes, degrees of freedom and P value noted<br>*Give P values as exact values whenever suitable.*
[x]	[ ]	For Bayesian analysis, information on the choice of priors and Markov chain Monte Carlo settings
[x]	[ ]	For hierarchical and complex designs, identification of the appropriate level for tests and full reporting of outcomes
[x]	[ ]	Estimates of effect sizes (e.g. Cohen's *d*, Pearson's *r*), indicating how they were calculated

Our web collection on statistics for biologists contains articles on many of the points above.

Software and code

Policy information about availability of computer code

Data collection	KEGG: Kyoto Encyclopedia of Genes and Genomes (https://www.kegg.jp/kegg/)
Data analysis	Prism Graphpad (v10), Microsoft Excel for Mac (v16) , BioVenn

For manuscripts utilizing custom algorithms or software that are central to the research but not yet described in published literature, software must be made available to editors and reviewers. We strongly encourage code deposition in a community repository (e.g. GitHub). See the Nature Portfolio guidelines for submitting code & software for further information.

Data

Policy information about availability of data

All manuscripts must include a data availability statement. This statement should provide the following information, where applicable:

- Accession codes, unique identifiers, or web links for publicly available datasets
- A description of any restrictions on data availability
- For clinical datasets or third party data, please ensure that the statement adheres to our policy

Numerical data used to generate graphs are provided as Source Data. Unique identifiers for genes from all species analysed are listed in the text. Where appropriate seeds of accessions and transgenic lines are available from the corresponding author or NASC (https://arabidopsis.info)

Research involving human participants, their data, or biological material

Policy information about studies with human participants or human data. See also policy information about sex, gender (identity/presentation), and sexual orientation and race, ethnicity and racism.

Reporting on sex and gender	N/A
Reporting on race, ethnicity, or other socially relevant groupings	N/A
Population characteristics	N/A
Recruitment	N/A
Ethics oversight	N/A

Note that full information on the approval of the study protocol must also be provided in the manuscript.

Field-specific reporting

Please select the one below that is the best fit for your research. If you are not sure, read the appropriate sections before making your selection.

[x] Life sciences [ ] Behavioural & social sciences [ ] Ecological, evolutionary & environmental sciences

For a reference copy of the document with all sections, see nature.com/documents/nr-reporting-summary-flat.pdf

Life sciences study design

All studies must disclose on these points even when the disclosure is negative.

Sample size	We used three independent biological samples size following standard practice in the field.
Data exclusions	none
Replication	Data were collected from at least three independent replications for each experiment, and replicates showed comparable trends. Almost all experiments were also replicated at least three times (for a small number not enough seeds were available) with similar observations
Randomization	Randomization of positions for growing seedling agar plates was used were possible.
Blinding	Investigators were not blinded to group allocations because this was not deemed necessary

Reporting for specific materials, systems and methods

We require information from authors about some types of materials, experimental systems and methods used in many studies. Here, indicate whether each material, system or method listed is relevant to your study. If you are not sure if a list item applies to your research, read the appropriate section before selecting a response.

Materials & experimental systems

n/a	Involved in the study	
[ ]	[x]	Antibodies
[x]	[ ]	Eukaryotic cell lines
[x]	[ ]	Palaeontology and archaeology
[x]	[ ]	Animals and other organisms
[x]	[ ]	Clinical data
[x]	[ ]	Dual use research of concern
[ ]	[x]	Plants

Methods

n/a	Involved in the study	
[x]	[ ]	ChIP-seq
[x]	[ ]	Flow cytometry
[x]	[ ]	MRI-based neuroimaging

Antibodies

Antibodies used	For ChIP: Anti-HA (Sigma, H3663-200UL; 1:2500 dilution). For Western blots: Anti-HA (Sigma, H3663-200UL; 1:1000 dilution), secondary antibody, Goat anti-Mouse IgG1, HRP from Thermo Fisher Scientific, PA1 74421, 1:20000 dilution).
Validation	Validation of the antibodies has been reported by the producers and in many refereed publications.

Dual use research of concern

Policy information about dual use research of concern

Hazards

Could the accidental, deliberate or reckless misuse of agents or technologies generated in the work, or the application of information presented in the manuscript, pose a threat to:

No	Yes	
☒	☐	Public health
☒	☐	National security
☒	☐	Crops and/or livestock
☒	☐	Ecosystems
☒	☐	Any other significant area

Experiments of concern

Does the work involve any of these experiments of concern:

No	Yes	
☒	☐	Demonstrate how to render a vaccine ineffective
☒	☐	Confer resistance to therapeutically useful antibiotics or antiviral agents
☒	☐	Enhance the virulence of a pathogen or render a nonpathogen virulent
☒	☐	Increase transmissibility of a pathogen
☒	☐	Alter the host range of a pathogen
☒	☐	Enable evasion of diagnostic/detection modalities
☒	☐	Enable the weaponization of a biological agent or toxin
☒	☐	Any other potentially harmful combination of experiments and agents

Plants

Seed stocks	The mutant alleles hyt1-1 (SALK_052163) and hyt1-2 (GABI_809B11) were obtained from NASC (University of Nottingham, UK). Combination mutants and other transgenics were obtained by crossing with previously described mutants nia1 nia2 (https://doi.org:10.1104/pp.109.148023), prt6-1 (https://doi.org:10.1073/pnas.0810280106), prt6-1 erfVII https://doi.org:10.1038/s41586-022-04740-y, ate1 ate2 (https://doi.org:10.1073/pnas.0810280106).
Novel plant genotypes	Combination mutants and other transgenics were obtained by crossing. Novel constructs were transformed into Arabidopsis using standard protocols (Clough, S. J. & Bent, A. F. Floral dip: a simplified method for Agrobacterium-mediated transformation of Arabidopsis thaliana. Plant J. 16, 735-743 (1998).).At least three independent lines of each construct were initially shown to have similar phenotypes.All lines used were homozygous for all mutations/transgenes.
Authentication	All genetic resources were genotyped to confirm homozygosity. For novel gene analysis two homozygous mutant T-DNA alleles were assessed.
