# Supplementary material for: Hierarchical regulation of oxygen-sensing through coupled transducers in Arabidopsis thaliana
Source: Nat Commun. 2026 Sep 16;17:10861. doi: 10.1038/s41467-026-77710-x (PMC13672779; doi:10.1038/s41467-026-77710-x)
Supplement: Supplementary file 4 [file 41467_2026_77710_MOESM9_ESM.pdf]

nature portfolio

Peer Review File

Hierarchical regulation of oxygen-sensing through coupled transducers in Arabidopsis thaliana

Corresponding Author: Professor Michael Holdsworth

This file contains all reviewer reports in order by version, followed by all author rebuttals in order by version.

Version 0:

Reviewer comments:

Reviewer #1

(Remarks to the Author)
This study presents high-quality research with novel findings, identifying HYT1 as a new substrate of the ATE/PRT6 N-end rule pathway, providing an important contribution to our understanding of oxygen sensing in plants. The authors used complementary approaches, including mass spectrometry, immunoblot, gene expression analysis, confocal microscopy, and phenotypic characterization, which together provide a solid and comprehensive study.
Overall, I consider the manuscript is suitable for publication after minor revisions. While the study is well constructed, several aspects could be further improved. In particular, the figure legends are relatively brief, and more detailed descriptions should be added to better explain the experimental design and results.
Specific points:
1, Line 90-91: The authors state that 49 Arabidopsis proteins initiating with Met-Cys2 are upregulated under hypoxia. However, it is unclear which 49 genes are referred to in Supplementary Table 1. Please clarify this by adding a detailed legend or clearly indicating these genes in the table.1
2, Fig1d: It is unclear how Cys2A-HYT1-3×HA accumulation was specifically measured in WT and prt6-1 seedlings. Is the indicated Ala2-HYT band detected using a specific antibody? I assume the experiment was performed using Arabidopsis lines expressing 35S:UFT-Cys2Ala-HYT1-3×HA, but this is not explicitly stated. Please add methodological details in both the main text and figure legend.
3, Fig2a: The reported m/z difference shown in the figure corresponds to ~16 Da (consistent with a 2+ charged peptide) rather than 32 Da (also it is incorrectly labeled kDa in the figure) between modified and unmodified peptides. Please revise or clarify this point.
4, Fig2b, This panel is not cited in the main text. The charge state notation is inconsistent. For example, b2 is labeled as +1, whereas in other MS/MS spectra singly charged ions are not labeled. Please ensure consistency across all figures.
In addition, the notation $(M+2H)^{3+}$ is incorrect and should be $(M+3H)^{3+}$. The same for in Extended Data Figure 2d.
5, Line 182: The expressions “prt6-1 < prt6-1 hyt1-1” and “prt6-1 > prt6-1 hyt1-1” are not clearly defined. Please explicitly clarify what these comparisons represent This issue also applies to the legend of Extended Data Figure 5a, which is currently difficult to interpret.
6, Line 194-195: The statement regarding ADH1, PCO1, and SUS4 gene expression appears inconsistent with the data, at least no significant changes are observed between WT and hyt1-1 within the first 30 minutes of submergence. Please revise the statement to more accurately reflect the data.
7, Fig.3g: Please specify whether “Relative expression to Col-0” refers to Col-0 under control (non-submerged) conditions in the figure legend.
8, Fig4d: Please specify what PI staining represents in the legend. It is unclear why PI shows a strong signal in 35S:UFT-C2-HYT1-3×HA under control conditions, while almost no signal is observed in 35S:UFT-C2-RAP2.3-3×HA. Please provide an explanation.
9, Fig5d: The authors analyze HYT1 expression in Arabidopsis accessions from different altitudes, but no detailed information about these accessions is provided. Please include additional details in the figure legend and/or Materials and Methods. It would also be helpful to further elaborate on the insights derived from this result.
10, It may be worth considering replacing the current simple model in Figure 6 with the expanded model shown in Extended Data Figure 12.

Reviewer #2

(Remarks to the Author)
The manuscript NCOMMS-26-034890 entitled “Hierarchical regulation of oxygen-sensing through coupled transducers in Arabidopsis thaliana” investigates how molecular oxygen sensing is translated into downstream physiological and developmental plant responses. The study presents significant conceptual and biochemical advances by identifying HYT1 (formerly AT4G24110 / HUP40) as a novel, conserved, angiosperm-specific substrate of the PCO/PRT6 N-degron pathway. Unlike previously identified substrates, which are predominantly transcription factors like ERFVIIs, ZPR2, and VRN2, HYT1 is a tail-anchored (TA) protein localized to the ER/nuclear envelope and the nucleoplasm. The authors demonstrate that HYT1 stability is dynamically controlled by oxygen and nitric oxide via Cys oxidation and subsequent degradation. Furthermore, they establish that ERFVII transcription factors directly regulate HYT1 transcription, forming a coherent feed-forward loop that fine-tunes plant responses to persistent hypoxia.
The work substantially broadens our understanding of the plant N-degron pathway beyond transcriptional regulation. It also rectifies a long-standing mis-annotation in the literature by definitively disproving the classification of AT4G24110 as a glutamate dehydrogenase through robust structural, enzymatic, and sequence analyses. The findings represent a high-impact contribution to plant environmental stress signaling.
The experimental design is rigorous, combining state-of-the-art mass spectrometry (MS/MS) for oxidation state verification, molecular biology, structural modeling, genetic mutant analysis, confocal microscopy for subcellular localization, and transcriptomics (RNA-seq).
Accept with Minor Revisions. The study is methodologically rigorous, logically sound, and presents an exciting novelty for plant oxygen signaling. Addressing the minor mechanistic and localization points above will render the paper fully ready for publication in Nature Communications.

Minor Revisions

Lack of N-terminal Arginylation in Wheat Germ Extract: In line 145, the authors note that while Nt-Cys oxidation to Cys-sulfonate was detected for HYT1, N-terminal arginylation by ATE was not observed in the wheat germ extract, whereas it was detected for RAP2.3. The authors suggest HYT1 Nt-Cys might be a weaker ATE substrate. Could the authors comment on whether Arg-3 (which is highly conserved in HYT1-like proteins) might sterically or electrostatically influence ATE binding efficiency in vitro?

PCO Oxidation State: Recombinant PCO4 catalyzed addition of 2 oxygen atoms (sulfinic acid, +32" Da") on the synthetic peptide (Fig. 2a), whereas incubation in wheat germ extract yielded tri-oxidized Cys-sulfonic acid (+48" Da") (Fig. 2b). The authors hypothesize an additional monooxygenase might be involved in plant systems. A brief clarification or discussion on whether this could alternatively be an artifact of non-enzymatic oxidation in cell extracts would strengthen the text.

Dual ER / Nuclear Localisation: In Fig. 4d–e and Extended Data Fig. 11, GFP-HYT1 is shown to localize to both the ER/nuclear envelope (as expected for a TA protein) and inside the nucleoplasm. Since TA domains are typically integrated post-translationally into membranes via TRC40/GET pathways, how do the authors envision full-length HYT1 residing inside the nucleus? Is there a possibility of proteolytic processing, or does the predicted NLS (Extended Data Fig. 10) override/compete with TA targeting?

RNA-seq interpretation: RNA-seq comparing prt6-1 vs prt6-1 hyt1-1 revealed few major transcriptomic changes in normoxia, but hyt1-1 impaired core hypoxia gene induction during active submergence (Fig. 3g). The authors state that HYT1 regulates the kinetics of hypoxia gene accumulation rather than direct transcription. It would be helpful to expand briefly on how a TA protein localized at the ER/nuclear membrane could modulate transcript accumulation kinetics (e.g., mRNA transport, protein turnover of secondary regulators).

Reviewer #3

(Remarks to the Author)
This paper describes the identification of HYT1 as a PCO-regulatd N-degron target that is a modulator of the plant hypoxic response. It clearly and systematically demonstrates that HYT1 (previously known as HUP40) is stabilised under conditions of hypoxia and/or where the N-degron pathway is disrupted. They demonstrate, including through detailed MSMS analysis, that the N-terminal Cys is oxidised to Cys-sulfinate in a biochemical assay, and Cys-sulfonate in a wheat germ assay, but that arginylation was not detected in the latter (nor tested for in the former). They show that HYT1 contributes to some ERFVII-mediated stress responses and through a series of mutants, they determine that HYT1 impacts the expression of a small set of genes independently of ERFVIIs. They demonstrate convincingly that HYT1 is not an NADP+-specific glutamate dehydrogenase, as it has been annotated, and show that it localises to nuclear and ER membranes, likely via its TA domain. Finally they validate that HYT1 is ERF-VII induced, as previously predicted given it is a hypoxia-induced gene.

Overall, the experiments are rigorous and the data are robust. The paper will be of interest to a range of readers interested in plant low oxygen responses and the N-degron pathway, as it describes a novel hypoxia-sensitive target of this pathway. It is particularly interesting that it appears to be membrane tethered, and it is a shame that no further insight could be gained into its specific function. The authors might speculate a little more on this in the Discussion to stimulate ideas for follow-up work. In what I suspect is an effort to remain succinct, some important elements of the experimental design may be missed by a non-specialist, e.g. distinguishing between transgene-mediated constitutive and ERFVII-induced endogenous HYT1

expression and why this is important for interpretation of the results. The hypoxia/reoxygenation timecourse experiment was also difficult to interpret due to lack of experimental detail provided.

I have one additional experiment to suggest:

It is curious that HYT1 is not arginylated in the wheat germ IVTT system. As suggested by the authors, perhaps this is because the wheat ATE does not recognise (or only weakly recognises) some component of the HYT1 sequence that comes after the oxidised cysteine (maybe the conformation induced by the Pro at position 4?). Although many of the data in Figure 1 indicate that constitutively expressed HYT1 is indeed regulated by the N-degron pathway, it could be further secured by testing this with endogenously expressed HYT1. This is complicated by the induction of HYT1 expression by ERFVIIs which are themselves regulated by the N-degron pathway, but could perhaps be achieved by using a C2ARAP2.12 or C2ARAP2.3 under normoxia/hypoxia/BZ? This would seem important given HYT1 is proposed as a membrane-tethered protein and these are typically less likely to be degraded by the ubiquitin-proteasome system (this is also proposed in the Discussion lines 277-280, based on the time course of destabilisation however the time course data were difficult to interpret, as mentioned above and in more detail below).

I would also make the following suggestions for improved clarity/corrections:

1. I suggest including in the abstract that HUP40 has been renamed as HYT1 for widespread dissemination.

2. The experimental conditions for the time course experiments in Figure 1g/h are very difficult to establish – what are the timepoints referring to, what does ‘control’ mean, which is the RAP2.3 experiment? It is therefore very difficult to draw conclusions from these experiments. More information should be given in the Figure legend and/or the experimental protocol.

3. Figure 2a, mass increase is 32 Da, not 32 kDa.

4. Text at line 145 needs a reference to Figure 2b.

5. Should the MSMS ions in Fig 2b and Ext Data Fig 2d be $[M+3H]^{3+}$ rather than $[M+2H]^{3+}$?

6. Clarify the different conditions for data in Ext Data Fig. 4 compared to Figure 3A – is root tip survival measured immediately after hypoxia in ED Fig 4A?

7. Source data tab names need correcting – Extended Data Figure 4 data tab is labelled as Extended Data Figure 3, etc.

8. In the main text, line 198 onwards, NAD/NADP should be annotated in its positively charged form (NAD^+/$NADP^+$)

Open Access This Peer Review File is licensed under a Creative Commons Attribution 4.0 International License, which permits use, sharing, adaptation, distribution and reproduction in any medium or format, as long as you give appropriate credit to the original author(s) and the source, provide a link to the Creative Commons license, and indicate if changes were made.

In cases where reviewers are anonymous, credit should be given to 'Anonymous Referee' and the source.
The images or other third party material in this Peer Review File are included in the article's Creative Commons license, unless indicated otherwise in a credit line to the material. If material is not included in the article's Creative Commons license and your intended use is not permitted by statutory regulation or exceeds the permitted use, you will need to obtain permission directly from the copyright holder.
To view a copy of this license, visit https://creativecommons.org/licenses/by/4.0/

RESPONSE TO REVIEWER COMMENTS NCOMMS-26-034890

Responses are show in red text colour

Changes in the manuscript text are also highlighted in red

Reviewer #1 (Remarks to the Author):

This study presents high-quality research with novel findings, identifying HYT1 as a new substrate of the ATE/PRT6 N-end rule pathway, providing an important contribution to our understanding of oxygen sensing in plants. The authors used complementary approaches, including mass spectrometry, immunoblot, gene expression analysis, confocal microscopy, and phenotypic characterization, which together provide a solid and comprehensive study.

Overall, I consider the manuscript is suitable for publication after minor revisions. While the study is well constructed, several aspects could be further improved. In particular, the figure legends are relatively brief, and more detailed descriptions should be added to better explain the experimental design and results.

Specific points:

1, Line 90-91: The authors state that 49 Arabidopsis proteins initiating with Met-Cys2 are upregulated under hypoxia. However, it is unclear which 49 genes are referred to in Supplementary Table 1. Please clarify this by adding a detailed legend or clearly indicating these genes in the table.1

There is a misunderstanding of the sentence here, which has been rewritten to improve clarity. Only three of the core 49 hypoxia induced transcripts initiate Met Cys, one of which is HYT1.

2, Fig1d: It is unclear how Cys2A-HYT1-3×HA accumulation was specifically measured in WT and prt6-1 seedlings. Is the indicated Ala2-HYT band detected using a specific antibody? I assume the experiment was performed using Arabidopsis lines expressing 35S:UFT-Cys2Ala-HYT1-3×HA, but this is not explicitly stated. Please add methodological details in both the main text and figure legend.

Additional text has been added to clarify this:

"A Cys2Ala substitution in WT (transgene *35S:UFT-Cys2Ala-HYT1-3xHA*) also resulted in constitutive stabilisation (Fig. 1d)."

And the Figure legend is also updated for clarity

3, Fig2a: The reported m/z difference shown in the figure corresponds to ~16 Da (consistent with a 2+ charged peptide) rather than 32 Da (also it is incorrectly labeled

kDa in the figure) between modified and unmodified peptides. Please revise or clarify this point.

We thank the referee for this point. The (unitless) m/z difference is indeed +16 (z = 2), but the mass difference is 32 Da, as shown.

We thank the reviewer for finding the kDa mistake, that has been corrected to Da.

4, Fig2b, This panel is not cited in the main text. The charge state notation is inconsistent. For example, b2 is labeled as +1, whereas in other MS/MS spectra singly charged ions are not labeled. Please ensure consistency across all figures.

We apologise for this omission, Fig 2b is now correctly highlighted in the manuscript text.

We thank the referee for this point. For Fig. 2c all ions are singly charged, and this has been noted in the figure legend to state this explicitly for this figure part.

In addition, the notation $(M+2H)^{3+}$ is incorrect and should be $(M+3H)^{3+}$. The same for in Extended Data Figure 2d.

Thank you to the reviewer for finding this error, that has been corrected in all occurrences

5, Line 182: The expressions “prt6-1 < prt6-1 hyt1-1” and “prt6-1 > prt6-1 hyt1-1” are not clearly defined. Please explicitly clarify what these comparisons represent This issue also applies to the legend of Extended Data Figure 5a, which is currently difficult to interpret.

The text in the manuscript has been changed for increased clarity to:

“(only 25 transcripts are more than 2 fold up regulated in prt6-1 hyt1-1 compared to prt6-1, and 90 more than 2 fold up regulated in prt6-1 compared to prt6-1 hyt1-1)”

Extended Data Figure 5a now includes the text:

“All transcripts up-regulated in the described comparisons were included in the analysis. Hypoxia up-regulated transcripts were obtained from Gibbs *et al* (2014) Molecular Cell; 53 369-379. Core 49 hypoxia up-regulated genes were obtained from Mustroph et al. (2009) PNAS (USA); 106 18843-18848.“

6, Line 194-195: The statement regarding ADH1, PCO1, and SUS4 gene expression appears inconsistent with the data, at least no significant changes are observed between WT and hyt1-1 within the first 30 minutes of submergence. Please revise the statement to more accurately reflect the data.

The statement has been revised to more accurately describe the results obtained:

“Whereas there was no difference in expression of hypoxia-related transcripts by 30 minutes of submergence, following 60 minutes of submergence accumulation of *ADH1* and *PCO1*

were reduced in *erfVII* and in *hyt1-1* compared to WT and *35S:UFT-Cys2-RAP2.3-3xHA* (Fig. 3g)."

7, Fig.3g: Please specify whether "Relative expression to Col-0" refers to Col-0 under control (non-submerged) conditions in the figure legend.

This has been clarified in the figure legend

8, Fig4d: Please specify what PI staining represents in the legend. It is unclear why PI shows a strong signal in 35S:UFT-C2-HYT1-3×HA under control conditions, while almost no signal is observed in 35S:UFT-C2-RAP2.3-3×HA. Please provide an explanation.

PI is an abbreviation for Propidium Iodide and is commonly used counterstain. We are sorry for the oversight and this has now been corrected in Fig 4d, ext added: "Counterstaining was done using Propidium Iodide (PI)."

It is not that there is no signal in 35S:UFT-C2-RAP2.3-3×HA control but we agree that PI signal is weaker in this case. This is simply due to the nature of the PI staining which can be variable and time dependent. Also, you will notice that the two images are not in the same focal planes. For sake of clarity, we have chosen the surface view for the HYT images. Outermost cells will get stained first and faster with PI and as a result, the surface views show brighter fluorescence. As a direct correct comparison for the RAP images, we show below a comparable images of HYT going through the same focal plane.

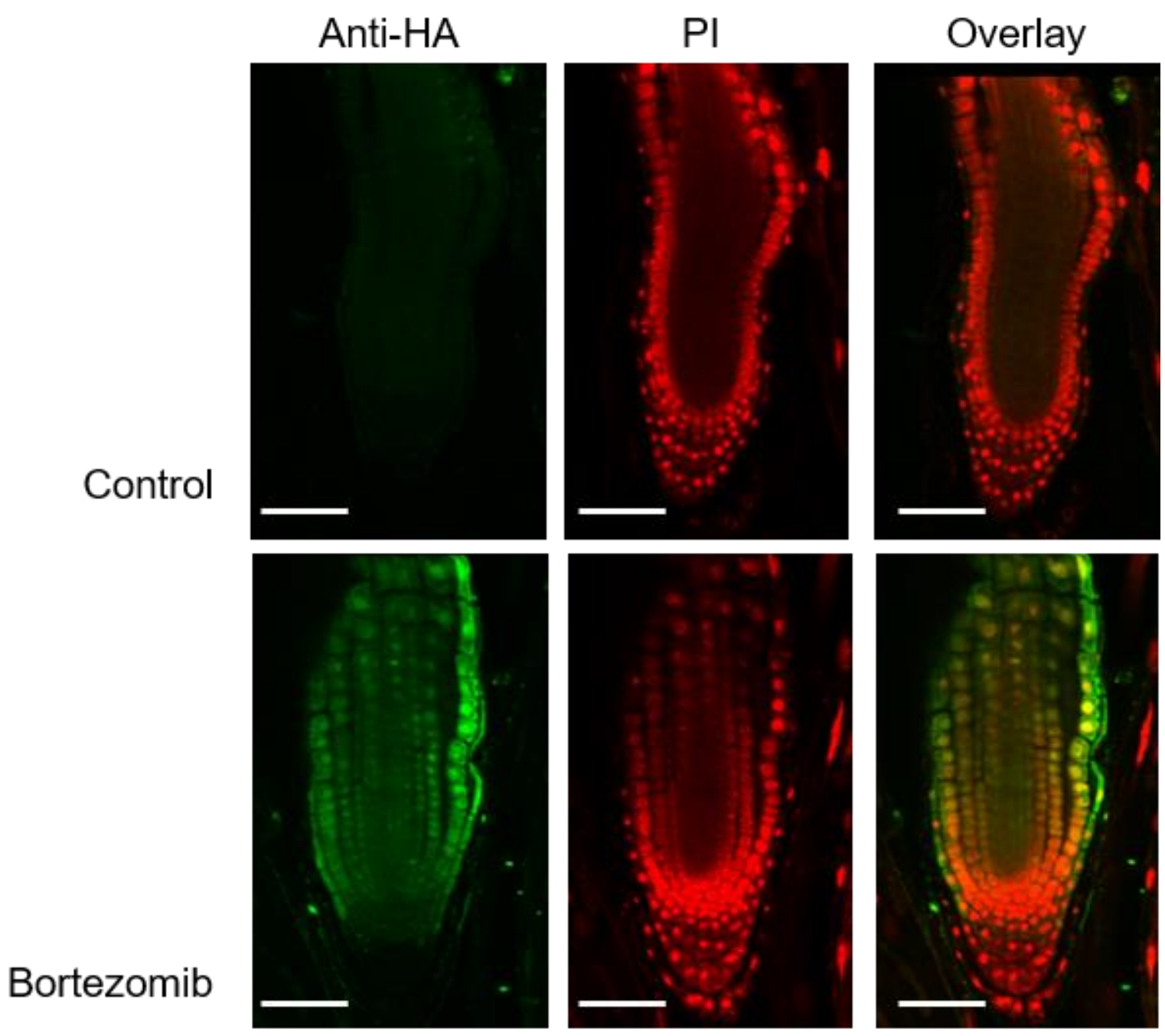

9, Fig5d: The authors analyze HYT1 expression in Arabidopsis accessions from different altitudes, but no detailed information about these accessions is provided.

Please include additional details in the figure legend and/or Materials and Methods. It would also be helpful to further elaborate on the insights derived from this result.

We thank the reviewer for this observation. Text is now added to the figure legend:

“Accessions used and altitudes are: Col-0 100; Bay-0 400; Sne 2662; Sha 3400 m a.s.l.”

The following text was added to the discussion in relation to this experiment:

“The abundance of ERFVII proteins was previously also shown to be regulated by altitude adaptation (relatively lower abundance in higher altitude accessions at higher ambient oxygen levels), and consistent with this here we observed lower expression of HYT1 at high oxygen in accessions from higher altitudes, that was increased at lower oxygen levels, indicating that HYT1 transcripts are controlled by ERFVIIs through altitude adaptation.”

10, It may be worth considering replacing the current simple model in Figure 6 with the expanded model shown in Extended Data Figure 12.

The current Figure 6 model is depicted as a mathematical representation of a coherent feed-forward loop, whereas Extended Data Figure 12 is simply a biological description of the system, therefore we would prefer to keep the simplified model in the main results section.

Reviewer #2 (Remarks to the Author):

The manuscript NCOMMS-26-034890 entitled “Hierarchical regulation of oxygen-sensing through coupled transducers in Arabidopsis thaliana” investigates how molecular oxygen sensing is translated into downstream physiological and developmental plant responses. The study presents significant conceptual and biochemical advances by identifying HYT1 (formerly AT4G24110 / HUP40) as a novel, conserved, angiosperm-specific substrate of the PCO/PRT6 N-degron pathway.

Unlike previously identified substrates, which are predominantly transcription factors like ERFVIIs, ZPR2, and VRN2, HYT1 is a tail-anchored (TA) protein localized to the ER/nuclear envelope and the nucleoplasm. The authors demonstrate that HYT1 stability is dynamically controlled by oxygen and nitric oxide via Cys oxidation and subsequent degradation. Furthermore, they establish that ERFVII transcription factors directly regulate HYT1 transcription, forming a coherent feed-forward loop that fine-tunes plant responses to persistent hypoxia.

The work substantially broadens our understanding of the plant N-degron pathway beyond transcriptional regulation. It also rectifies a long-standing mis-annotation in the literature by definitively disproving the classification of AT4G24110 as a

glutamate dehydrogenase through robust structural, enzymatic, and sequence analyses. The findings represent a high-impact contribution to plant environmental stress signaling.

The experimental design is rigorous, combining state-of-the-art mass spectrometry (MS/MS) for oxidation state verification, molecular biology, structural modeling, genetic mutant analysis, confocal microscopy for subcellular localization, and transcriptomics (RNA-seq).

Accept with Minor Revisions. The study is methodologically rigorous, logically sound, and presents an exciting novelty for plant oxygen signaling. Addressing the minor mechanistic and localization points above will render the paper fully ready for publication in Nature Communications.

Minor Revisions

Lack of N-terminal Arginylation in Wheat Germ Extract: In line 145, the authors note that while Nt-Cys oxidation to Cys-sulfonate was detected for HYT1, N-terminal arginylation by ATE was not observed in the wheat germ extract, whereas it was detected for RAP2.3. The authors suggest HYT1 Nt-Cys might be a weaker ATE substrate. Could the authors comment on whether Arg-3 (which is highly conserved in HYT1-like proteins) might sterically or electrostatically influence ATE binding efficiency in vitro?

We thanks the reviewer for this suggestion. It is true that the ERFVIIs do not have Arg3, whereas interestingly VRN2 does have Arg3, though its arginylation status in vitro has not been reported. Reviewer 3 also suggested that Pro5 might also have a steric effect. We note that Wadas et al (2016) J. Biol. Chem. 291, p20976-20992 showed that Arg at position 2 following N-terminal D had a positive effect on arginylation capacity compared to other residues at that position.

We added to the discussion:

“This may be due to steric effects of residues closely associated with N-terminal Cys other or structural features of the protein.“

PCO Oxidation State: Recombinant PCO4 catalyzed addition of 2 oxygen atoms (sulfinic acid, +32" Da") on the synthetic peptide (Fig. 2a), whereas incubation in wheat germ extract yielded tri-oxidized Cys-sulfonic acid (+48" Da") (Fig. 2b). The authors hypothesize an additional monooxygenase might be involved in plant systems. A brief clarification or discussion on whether this could alternatively be an artifact of non-enzymatic oxidation in cell extracts would strengthen the text.

We thank the referee for this suggestion. We note that Cys-sulfonic acid was also observed for human RGS4 from mouse L cells through a different methodology. We

investigated the origin of the third oxygen in detail in Zubrycka et al Nat Comms (2023) and showed that it was derived from molecular oxygen and not water.

We edited the following sentence in the discussion:

"Cys-oxidation to sulfonic acid was also shown to occur in RAP2.3 and RAP2.12[8], and also in the mammalian Cys-2 protein RGS4 purified from mouse L cells[9] indicating that in addition to PCO/ADO action (that adds two oxygen atoms to N-terminal Cys) it is possible that a monooxygenase is also involved in the pathway to add the third oxygen."

We previously investigated whether addition of an extra oxygen was an artifact related to post incubation MS workup for RAP2.3, and found that it was not, the following is stated in Zubrycka *et al* (2023) Nat. Comms:

"As a negative control for artefactual Cys oxidation, RAP2.3HIS was synthesised in E. coli, which does not possess the enzymatic machinery for Nt-Cys oxidative processing. As expected, no oxidation or subsequent arginylation was detected, and the unmodified Nt-tryptic peptide was found using the LC-MS/MS methodology with the Met1 removed (from the action of endogenous E. coli MetAP activity; Supplementary Fig. 5c)."

Dual ER / Nuclear Localisation: In Fig. 4d–e and Extended Data Fig. 11, GFP-HYT1 is shown to localize to both the ER/nuclear envelope (as expected for a TA protein) and inside the nucleoplasm. Since TA domains are typically integrated post-translationally into membranes via TRC40/GET pathways, how do the authors envision full-length HYT1 residing inside the nucleus? Is there a possibility of proteolytic processing, or does the predicted NLS (Extended Data Fig. 10) override/compete with TA targeting?

The referee raises an interesting point. We used the constructs GFP-HYT1 and HYT1-3xHA to analyse subcellular localisation, and in both cases observed HYT1 in the nucleus. Therefore, this is unlikely to be due to proteolytic processing, as observations were made based on N-terminal (GFP) or C-terminal (3xHA) tags. It appears possible therefore that as the referee suggests the predicted NLS overrides the TA targeting signal

RNA-seq interpretation: RNA-seq comparing prt6-1 vs prt6-1 hyt1-1 revealed few major transcriptomic changes in normoxia, but hyt1-1 impaired core hypoxia gene induction during active submergence (Fig. 3g). The authors state that HYT1 regulates the kinetics of hypoxia gene accumulation rather than direct transcription. It would be helpful to expand briefly on how a TA protein localized at the ER/nuclear membrane could modulate transcript accumulation kinetics (e.g., mRNA transport, protein turnover of secondary regulators).

We thank the referee for this interesting observation and have included the following text in the discussion:

“perhaps by influencing mRNA transport from the nucleus, or other processes related to transcript stability”

Reviewer #3 (Remarks to the Author):

This paper describes the identification of HYT1 as a PCO-regulatd N-degron target that is a modulator of the plant hypoxic response. It clearly and systematically demonstrates that HYT1 (previously known as HUP40) is stabilised under conditions of hypoxia and/or where the N-degron pathway is disrupted. They demonstrate, including through detailed MSMS analysis, that the N-terminal Cys is oxidised to Cys-sulfinate in a biochemical assay, and Cys-sulfonate in a wheat germ assay, but that arginylation was not detected in the latter (nor tested for in the former). They show that HYT1 contributes to some ERFVII-mediated stress responses and through a series of mutants, they determine that HYT1 impacts the expression of a small set of genes independently of ERFVIIs. They demonstrate convincingly that HYT1 is not an NADP+-specific glutamate dehydrogenase, as it has been annotated, and show that it localises to nuclear and ER membranes, likely via its TA domain. Finally they validate that HYT1 is ERF-VII induced, as previously predicted given it is a hypoxia-induced gene.

Overall, the experiments are rigorous and the data are robust. The paper will be of interest to a range of readers interested in plant low oxygen responses and the N-degron pathway, as it describes a novel hypoxia-sensitive target of this pathway. It is particularly interesting that it appears to be membrane tethered, and it is a shame that no further insight could be gained into its specific function. The authors might speculate a little more on this in the Discussion to stimulate ideas for follow-up work. In what I suspect is an effort to remain succinct, some important elements of the experimental design may be missed by a non-specialist, e.g. distinguishing between transgene-mediated constitutive and ERFVII-induced endogenous HYT1 expression and why this is important for interpretation of the results. The hypoxia/reoxygenation timecourse experiment was also difficult to interpret due to lack of experimental detail provided.

We thank the referee for raising these points, please see response to point 2 below in relation to hypoxia/reoxygenation time course.

I have one additional experiment to suggest:

It is curious that HYT1 is not arginylated in the wheat germ IVTT system. As suggested by the authors, perhaps this is because the wheat ATE does not

recognise (or only weakly recognises) some component of the HYT1 sequence that comes after the oxidised cysteine (maybe the conformation induced by the Pro at position 4?). Although many of the data in Figure 1 indicate that constitutively expressed HYT1 is indeed regulated by the N-degron pathway, it could be further secured by testing this with endogenously expressed HYT1. This is complicated by the induction of HYT1 expression by ERFVIIs which are themselves regulated by the N-degron pathway, but could perhaps be achieved by using a C2ARAP2.12 or C2ARAP2.3 under normoxia/hypoxia/BZ? This would seem important given HYT1 is proposed as a membrane-tethered protein and these are typically less likely to be degraded by the ubiquitin-proteasome system (this is also proposed in the Discussion lines 277-280, based on the time course of destabilisation however the time course data were difficult to interpret, as mentioned above and in more detail below).

We thank the referee for this suggested potential additional experiment. We would politely state that to attempt to recover HYT1 in vivo and then carry out MS/MS analysis of the N-terminus is not necessary for the conclusions of the paper. The biochemical genetics that we have carried out clearly show that HYT1 degradation is triggered by both ATE and PRT6, demonstrating that arginylation is a required step in its degradation under ambient oxygen levels in vivo. To attempt to assess the N-terminus derived from in vivo material has never been published for any substrate of the PCO pathway, including the ERFVIIs. Such an experiment would be a significant undertaking in both expenditure and time not warranted because it would not affect the conclusions of the study.

The important observation from the wheat germ experiments is that the N-terminus is oxidised by a plant extract to sulfonic acid, as we previously showed for RAP2.3 and RAP2.12, and that was also shown for mouse RGS4, and that all three added oxygen atoms are derived from molecular oxygen (see Zubrycka et al Nat Comms 2023).

I would also make the following suggestions for improved clarity/corrections:

1. I suggest including in the abstract that HUP40 has been renamed as HYT1 for widespread dissemination.

We thank the referee for this helpful suggestion: The following text was added to the abstract:

“Here we show that HYPOXIA TRANSDUCER1 (HYT1, previously named HUP40),”

2. The experimental conditions for the time course experiments in Figure 1g/h are very difficult to establish – what are the timepoints referring to, what does ‘control’ mean, which is the RAP2.3 experiment? It is therefore very difficult to draw

conclusions from these experiments. More information should be given in the Figure legend and/or the experimental protocol.

The reference to the similarity to the time course of stabilisation of RAP2.3 refers to that published in Zubrycka et al Nat Comms 2023, denoted by reference 8 in the sentence

"The time course of accumulation of HYT1-3xHA driven from the 35S promoter in response to submergence and hypoxia was similar to that previously observed for RAP2.3[8] (Fig. 1g,h) when driven from the 35S promoter."

The figure legend has been improved to clarify these points:

"g. Time-course analysis of HYT1-3xHA (from the transgene *35S:UFT-Cys2-HYT1-3xHA*) accumulation in WT seedlings in response to ambient dark hypoxia (<0.5 % O_2) and re-oxygenation. Times are from beginning of hypoxia treatment or release from the hypoxia chamber.

h. Time-course analysis of HYT1-3xHA (from the transgene *35S:UFT-Cys2-HYT1-3xHA*) accumulation in WT seedlings in response to dark submergence. Seedlings were submerged in the dark, or left only in the dark (control), for the indicated times."

3. Figure 2a, mass increase is 32 Da, not 32 kDa.

We thank the referee for finding this mistake, now rectified in the figure.

4. Text at line 145 needs a reference to Figure 2b.

We thank the referee for finding this mistake, now rectified in the text.

5. Should the MSMS ions in Fig 2b and Ext Data Fig 2d be [M+3H]3+ rather than [M+2H]3+?

Thank you to the reviewer for finding this error, that has been corrected in all occurrences

6. Clarify the different conditions for data in Ext Data Fig. 4 compared to Figure 3A – is root tip survival measured immediately after hypoxia in ED Fig 4A?

Experiments shown in Fig. 3A and Ex Data Fig. 4 were carried out at different times. They both measure root tip survival in response to a short acute hypoxia treatment, after 3 days recovery in normoxia. This is stated in the figure legend for figure 3a, and has been added to the legend of Ex Data Fig 4 to improve clarity.

7. Source data tab names need correcting – Extended Data Figure 4 data tab is labelled as Extended Data Figure 3, etc.

We thank the referee for finding this mistake, now rectified in the Source Data tabs.

8. In the main text, line 198 onwards, NAD/NADP should be annotated in its positively charged form (NAD^+/$NADP^+$)

We thank the referee for finding this mistake, now rectified in all instances the text.
