# Supplementary material for: Hierarchical regulation of oxygen-sensing through coupled transducers in Arabidopsis thaliana
Source: Nat Commun. 2026 Sep 16;17:10861. doi: 10.1038/s41467-026-77710-x (PMC13672779; doi:10.1038/s41467-026-77710-x)
Supplement: Supplementary file 5 [file 41467_2026_77710_MOESM10_ESM.zip › Original full size Westerns.pdf]

Manrique Gil *et al* Original full size Westerns, all mouse anti HA primary antibody (Sigma-Aldrich #H3663)

Fig. 1b

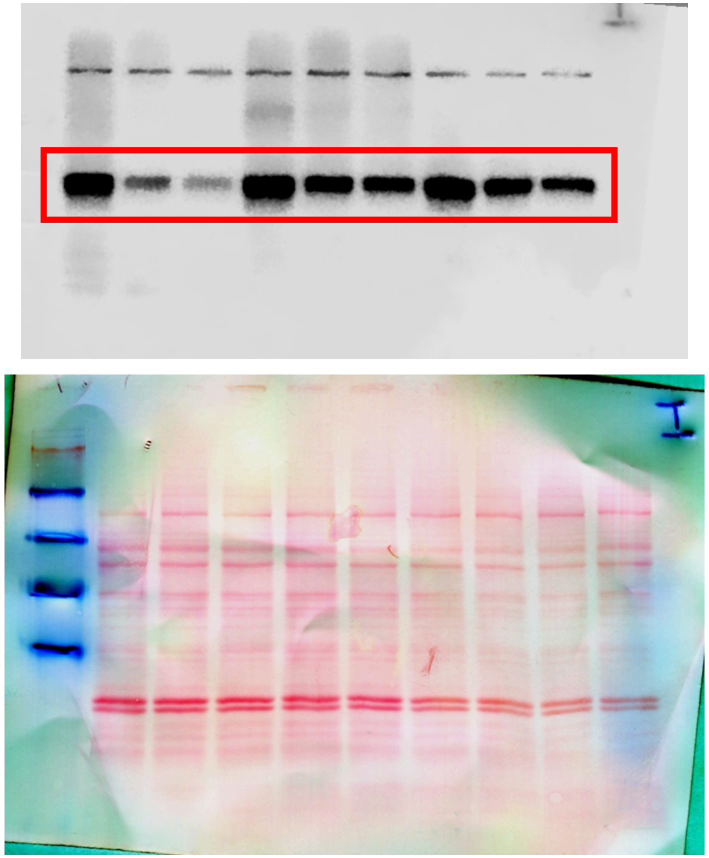

Fig 1g

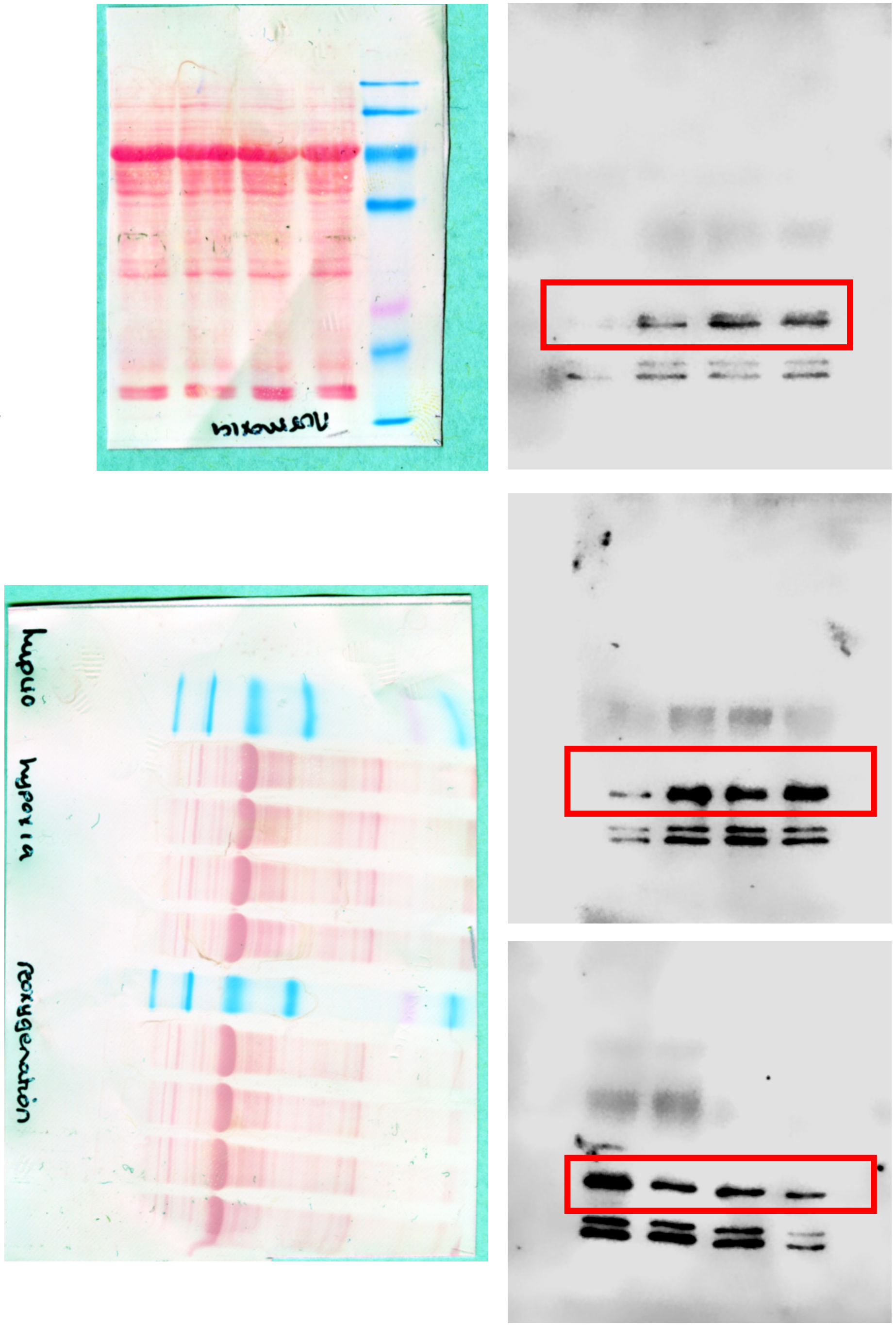

Cropped region

Fig 1f

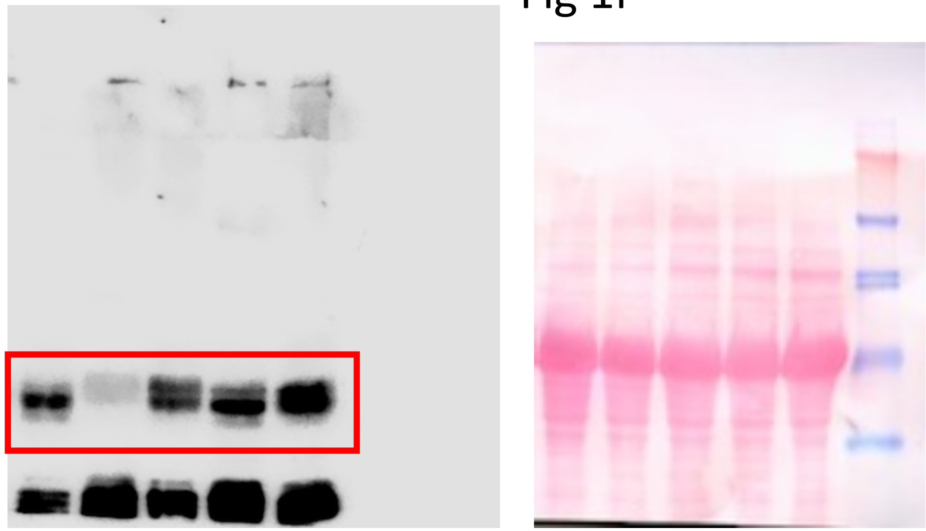

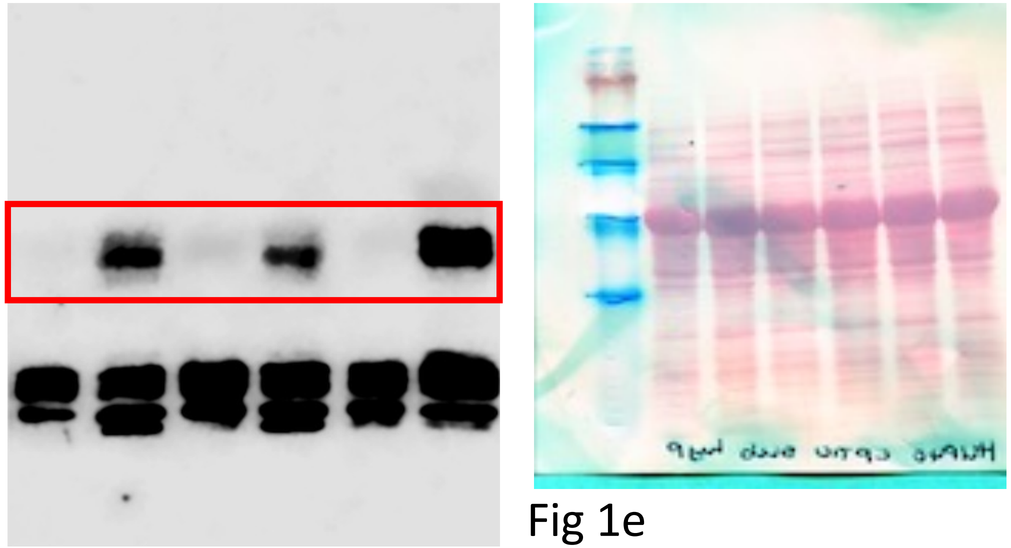

Fig 1e

Fig 1h

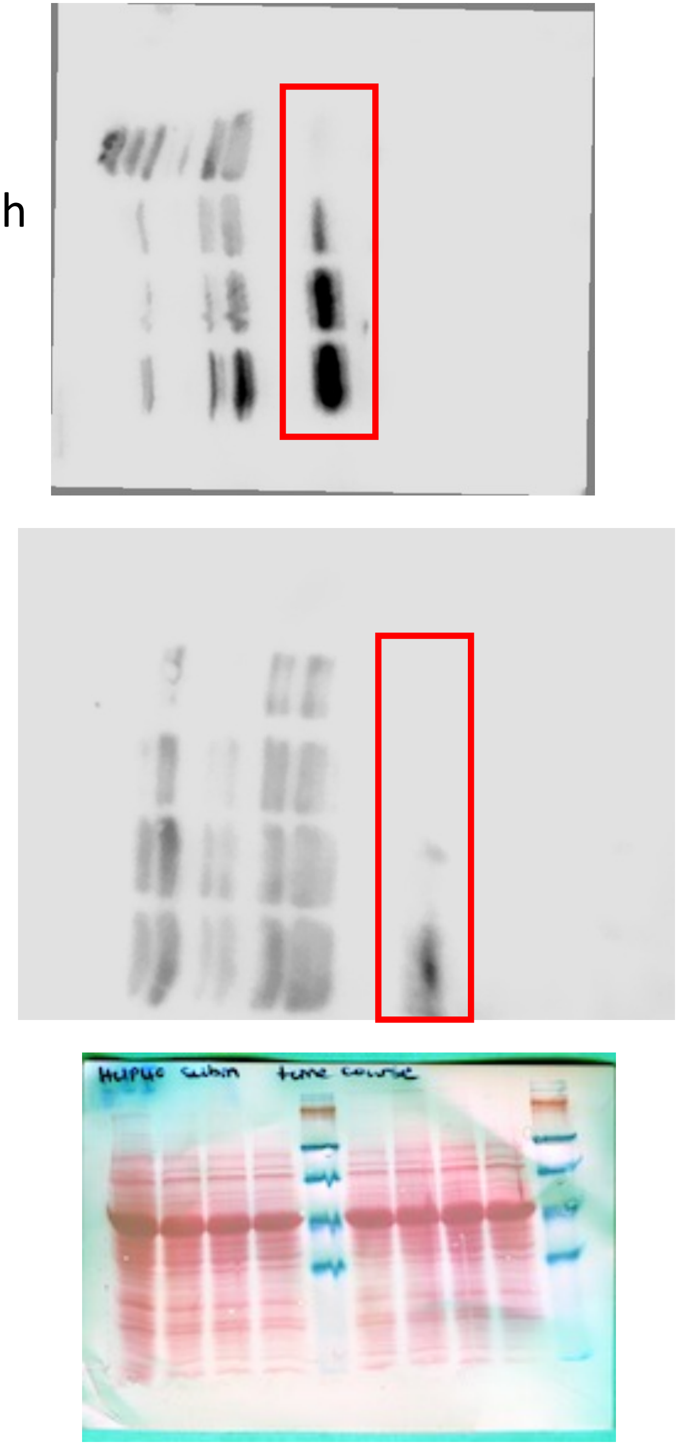

Cropped region

Fig 1d

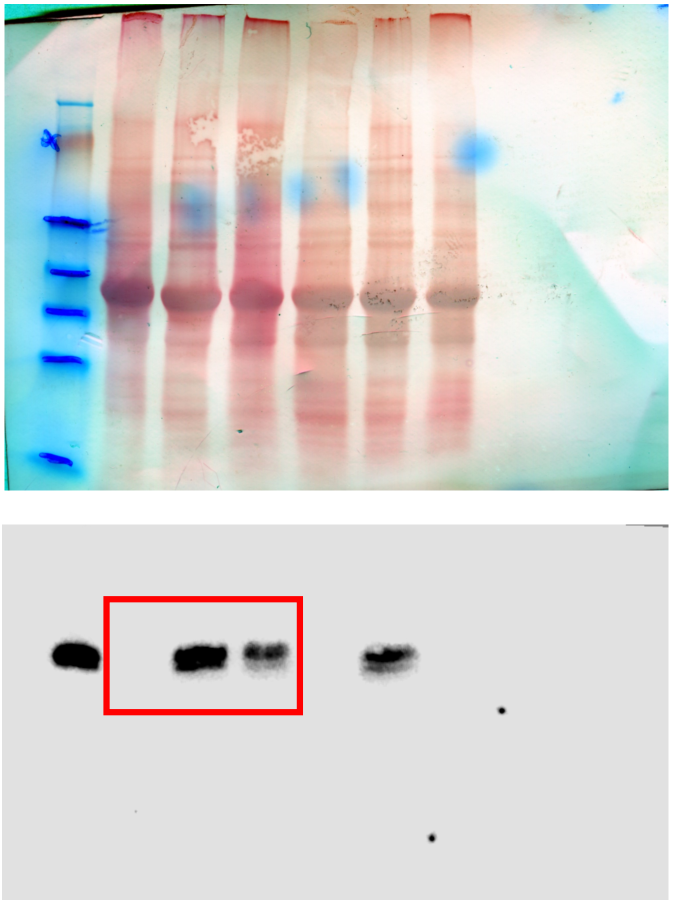

Fig 1c

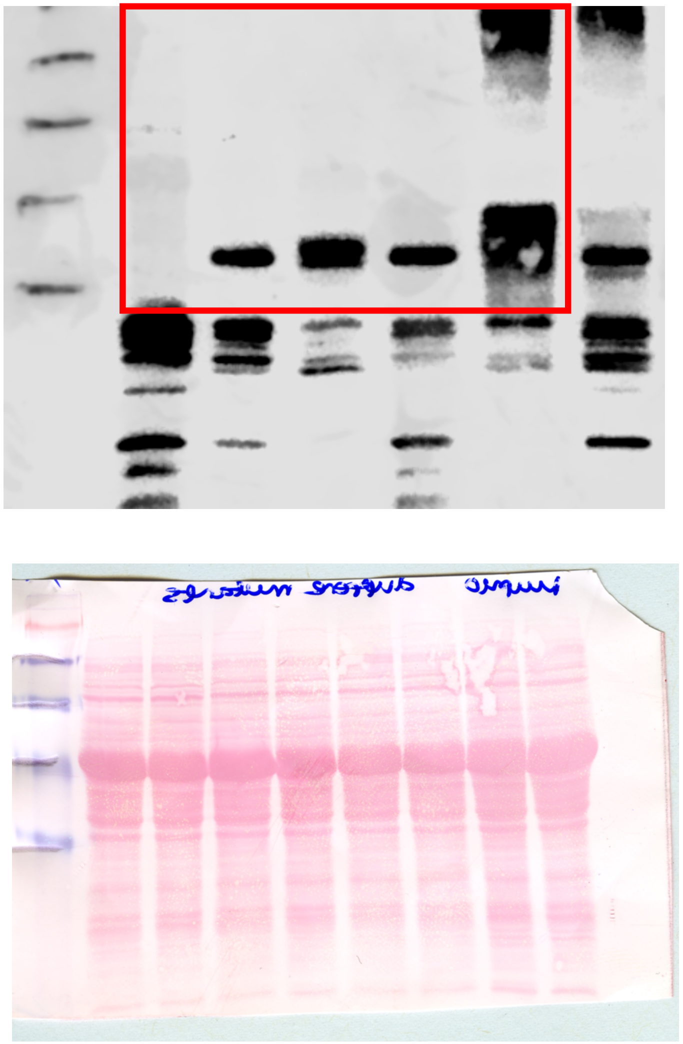

Cropped region
